# Access to 2,6-Dipropargylated BODIPYs as “Clickable” Congeners of Pyrromethene-567 Dye: Photostability and Synthetic Versatility

**DOI:** 10.1021/acs.orglett.1c02380

**Published:** 2021-08-17

**Authors:** Clara Uriel, Ana M. Gómez, Enrique García Martínez de la Hidalga, Jorge Bañuelos, Inmaculada Garcia-Moreno, J. Cristobal López

**Affiliations:** † Instituto de Química Orgánica General, IQOG-CSIC, Juan de la Cierva 3, 28006, Madrid, Spain; ‡ Departamento de Química Física. Universidad del Pais Vasco-EHU, Apartado 644, 48080, Bilbao, Spain; § Instituto de Química-Física “Rocasolano”, CSIC, Serrano 119, 28006, Madrid, Spain

## Abstract

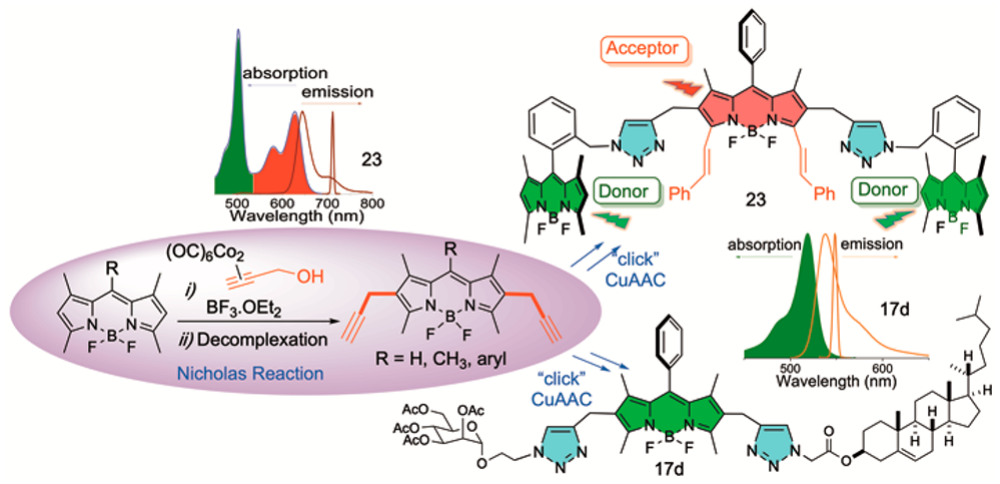

Hitherto unreported 2,6-dipropargyl-1,3,5,7-tetramethyl BODIPYs can be efficiently prepared by a Nicholas reaction/decomplexation protocol from 1,3,5,7-tetramethyl BODIPYs. The title compounds, which improve the BODIPY photostability by retaining their inherent photophysical and photochemical properties, can be engaged in efficient copper(I)-catalyzed azide–alkyne cycloaddition (CuAAC) “click-type” reactions with azido derivatives to provide all-BODIPY-triads or conjugated BODIPYs.

BODIPY (4,4′-difluoro-4-bora-3a,4a-diaza-s-indacene) dyes, e.g., **1** (Figure 1),^1^ have recently established themselves as one of the most appealing families among the arsenal of small-molecule fluorophores.^2^ BODIPYs have found ample applications in diverse areas ranging from biology to material sciences, e.g., photodynamic therapy,^3^ labeling of biomolecules,^4^ tunable laser dyes,^5^ organic photovoltaics, photosensitizers, and components in organic light-emitting diodes (OLEDs)^6^ and light harvesting systems.^7^ The reasons for their success can be credited to their remarkable photophysical properties, which include high fluorescence quantum yields and photostability, and large molar absorptivity. However, it is probably their chemical flexibility and stability that makes them the fluorophores of choice in a variety of applications. Thus, it has been shown that structure modifications can fine-tune their photophysical, physical, and chemical properties, and this has converted the pursuit of synthetic methods to incorporate a variety of functionalities in the BODIPY core in an active area of research. For instance, selected functionalization of the skeleton can induce bathochromic^8^ or hypsochromic^9^ shifts in their absorption and emission bands, water-solubility,^10^ and modification of their photostability.^11^ Regarding the latter, a variety of structural modifications aimed at improving the photochemical stability of commercially available pyrromethene 567 (PM567) laser dye **2** (Figure 1),^12^ frequently used as an internal reference for fluorescence quantum yields,^13^ have been examined, particularly at positions C-8, C-2, C-6, and at boron (Figure 1).^14^

**Figure 1:**
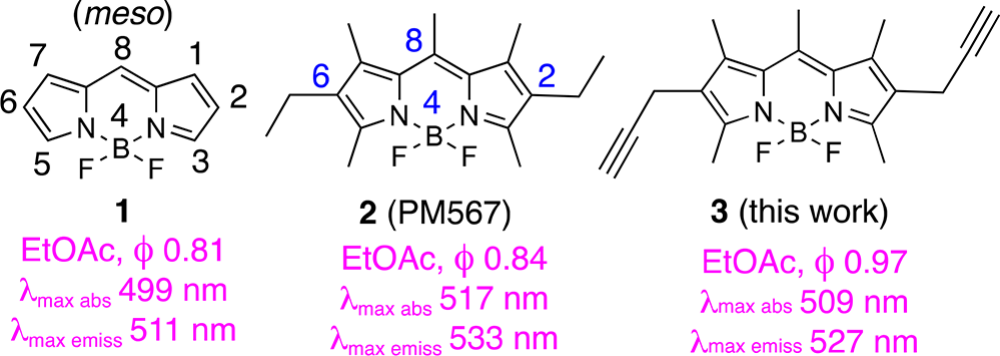
BODIPY **1**, pyrromethene 567 (PM567) **2**, and 2,6-dipropargyl BODIPY **3**, and spectroscopic data.

With these considerations in mind, we envisioned that hitherto unreported BODIPY **3** (Figure 1), a “clickable” analogue of PM567 (vide infra), could be an attractive laser-dye building-block with potential implications in some of the aforementioned applications. In this manuscript, we describe the synthesis, and some photophysical and photostability studies, of 2,6-dipropargyl BODIPY **3**, and some of its congeners. In addition, we have investigated their use as fluorescent tags and as components in light-harvesting BODIPY triads.

Initial propargylation studies were performed on 1,3,5,7-tetramethyl BODIPYs with *O*-propargyl trichloroacetimidate and related agents under a variety of reaction conditions.^15^ However, although in some instances, the desired 2,6-dipropargyl derivatives could be obtained, the transformation—in our hands—proved to be unreliable. Consequently, as a method to incorporate the propargyl substituent(s) to the BODIPY core, we selected the Nicholas reaction (promoted by a Lewis acid) in which an electrophilic propargylic cation, stabilized by a cobalt complex, reacts with a nucleophile.^16,17^ An additional decomplexation step is then necessary to unveil the desired alkyne moiety. Thus, overall the Nicholas reaction occurs with high regioselectivity at the propargylic position and has a wide scope in terms of reactive nucleophiles.

As the initial BODIPY to test the Nicholas reaction, we selected 1,3,5,7,8-pentamethyl BODIPY **4** (see Scheme 1). Accordingly, treatment of **4** with the dicobalthexacarbonyl complex of propargyl alcohol (**5**) in CH_2_Cl_2_, at −15 °C, in the presence of BF_3_**·**OEt_2_ (0.5 equiv), yielded alkynyl-cobalt intermediate **6**, which was decobaltated upon treatment with iodine in CH_2_Cl_2_ to yield 2,6-dipropargyl BODIPY **3** (see Scheme 1). Thus, according to the well-known reactivity of 1,3,5,7-tetramethyl BODIPYs,^18^ an electrophilic aromatic substitution (S_E_Ar) had taken place at positions C-2 and C-6 of the BODIPY core with the cobalt-stabilized propargyl cation arising from **5**.

**Scheme 1:**
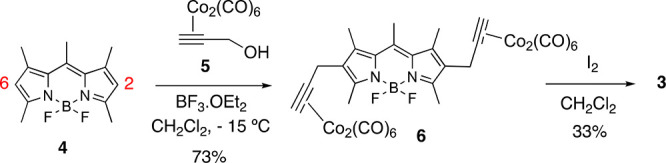
Nicholas Reaction of BODIPY **4** Leading to 2,6-Dipropargyl BODIPY **3**, by Iodine-Promoted Decobaltation of Synthetic Intermediate **6**

To evaluate the scope of the transformation, we next extended our studies to 1,3,5,7-tetramethyl BODIPYs **7a**–**7c**, differing in the substituents at the *meso*-position (Figure 2).

**Figure 2:**
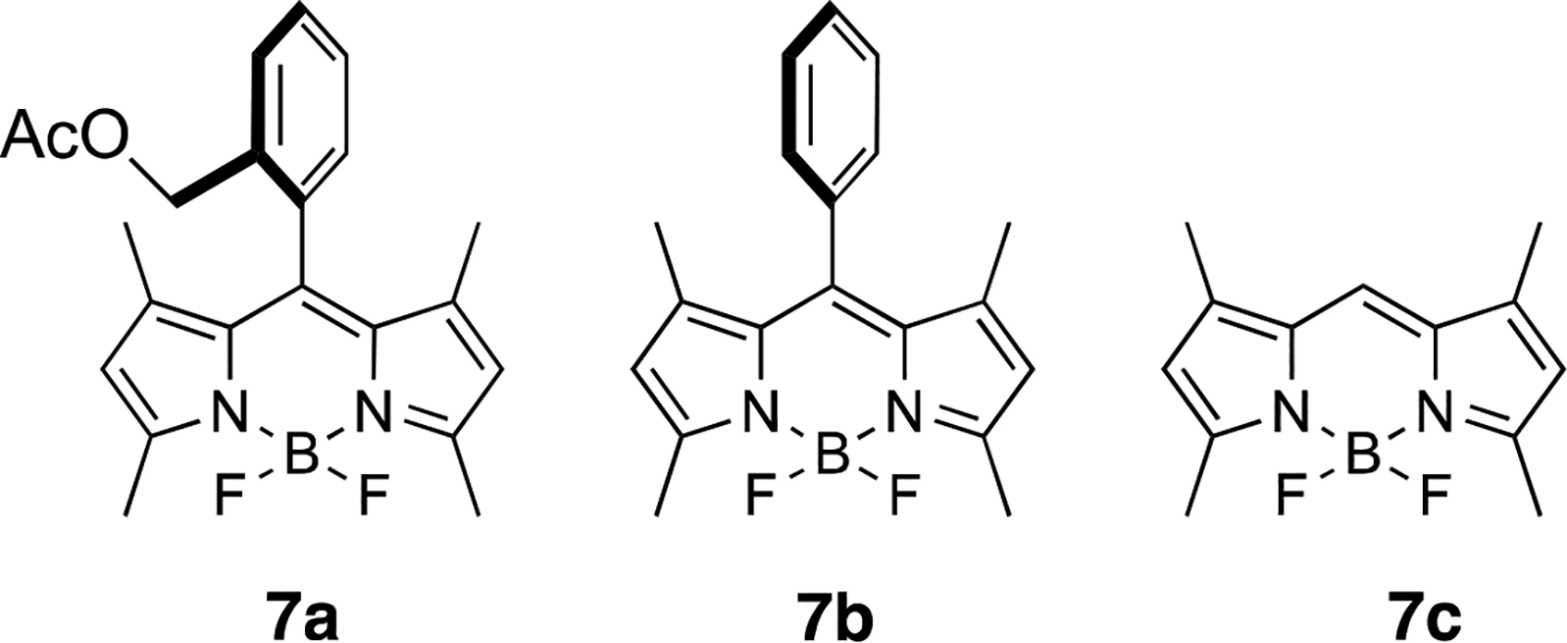
BODIPY derivatives **7a**–**7c**.

In keeping with the reaction conditions employed with BODIPY **4** (Scheme 1), tetramethyl-BODIPYs **7a**–**7c** were treated with **5** under the agency of BF_3_**·**OEt_2_ (0.5 equiv) in CH_2_Cl_2_, to furnish (after decobaltation) 2,6-dipropargyl BODIPYs **10a**–**10c** (see Scheme 2). Thus, upon treatment of BODIPYs **7a**–**7c** with 2.2 equiv of **5**, the corresponding dipropargylcobalt-BODIPY intermediates **8a**–**8c** could be obtained in good to excellent yields (Scheme 2 and Figure 3). Decomplexation of the latter to lead to 2,6-dipropargyl BODIPYs **10a**–**10c**, however, demanded some optimization of the reaction conditions. Thus, whereas iodine in THF or CH_2_Cl_2_ worked well with *meso*-aryl derivatives **8a** and **8b**, to furnish dipropargyl-BODIPYs **10a** and **10b** (Figure 3), 1,2-ethylenediamine in THF,^19^ had to be used with the more labile BODIPY derivative **8c**, to produce BODIPY **10c** (Figure 3). In addition, in order to assess the possibility of obtaining synthetically valuable monosubstituted propargyl BODIPYs, i.e., **11a**–**11c** (Scheme 2), the use of limited amounts of **5** (1.1 equiv) in the Nicholas reaction was also explored. Under these conditions, moderate amounts of monosubstituted propargyl-dicobalt-BODIPY intermediates **9a**–**9c** could be obtained, from which access to monopropargylated BODIPYs **11a**–**11c** could be obtained upon decobaltation (see Scheme 2 and Figure 3).

**Scheme 2:**
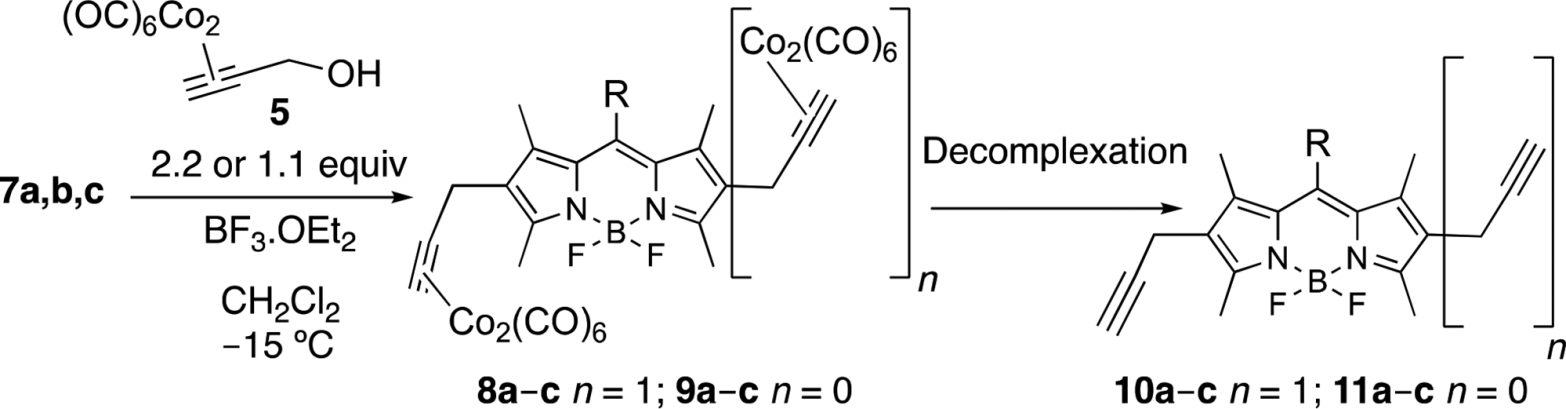
Synthesis of 2,6-Dipropargyl and 2-Propargyl BODIPY Derivatives **10a**–**10c** and **11a**–**11c**, Respectively, by Nicholas Reaction/Decomplexation of BODIPYs **7a**–**7c**

**Figure 3:**
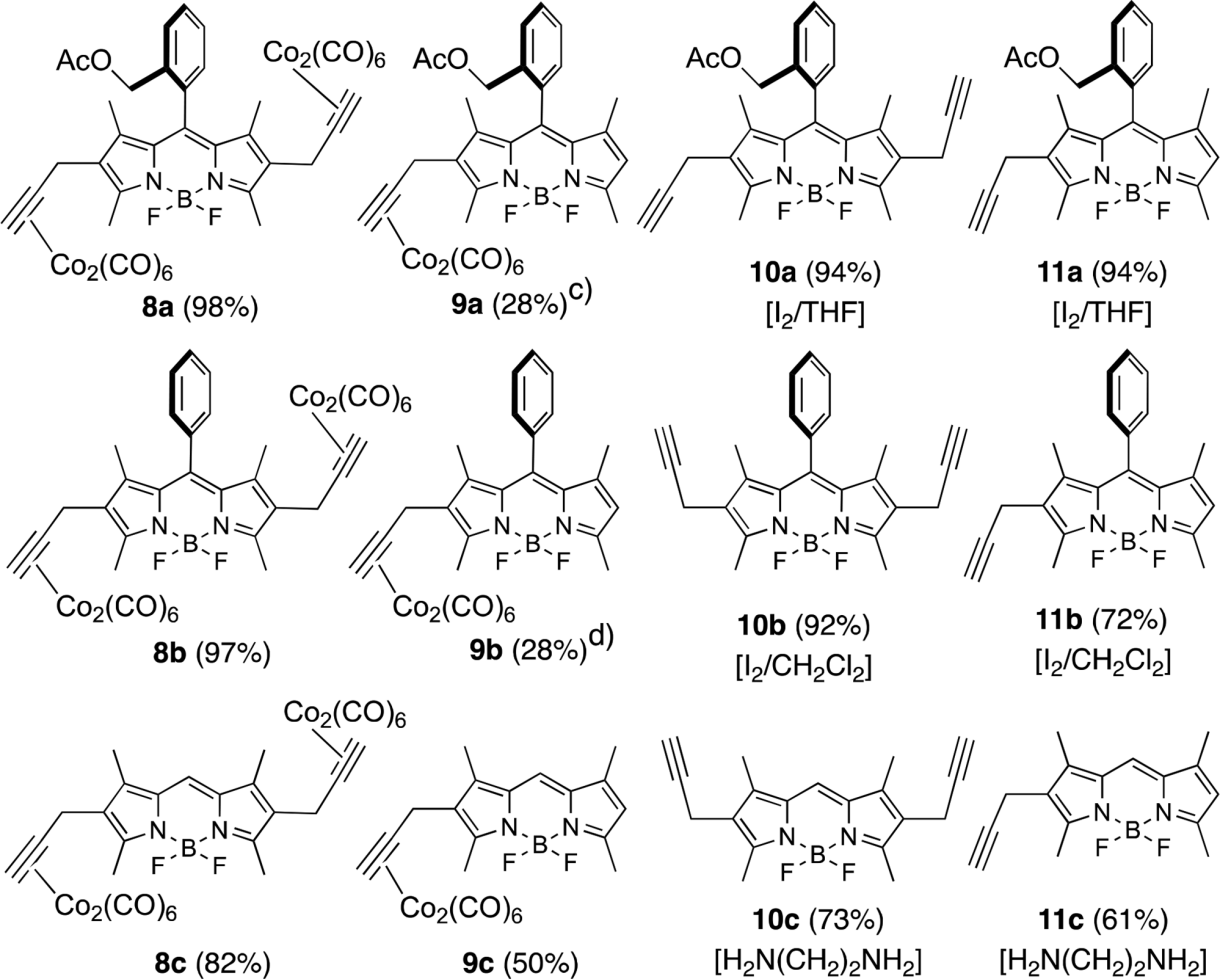
Monopropargyl and dipropargyl BODIPYs **11a**–**11c** and **9a**–**9c**, respectively, along with their intermediate dicobalt-propargyl derivatives **8a**–**8c** and **10a**–**10c**, obtained from BODIPYs **7a**–**7c**. Yields and decomplexation methods are included.

To obtain 2,6-dipropargyl-BODIPYs with improved photophysical properties, BODIPYs **3** and **10b** were transformed to B(CN)_2_-dipropargyl BODIPYs **12** and **13**, respectively, by treatment with TMSCN in CH_2_Cl_2_, in good to excellent yields (see Figure 4).^20^

**Figure 4:**
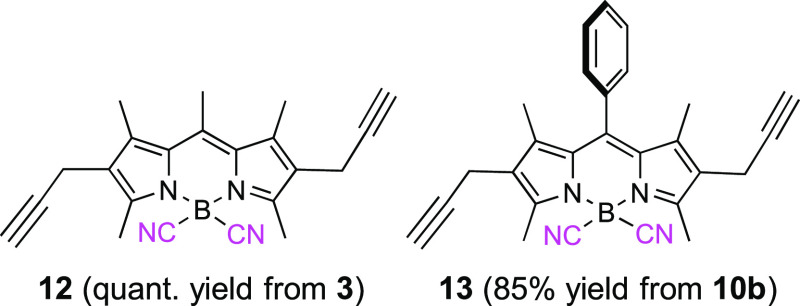
B(CN)_2_-BODIPYs **12** and **13**.

The propargylation of the methylene units grafted at position 2 and/or 6 of the BODIPY core does not alter its inherent photophysical properties (see Table S1 in the Supporting Information). Thus, all of the propargylated BODIPY derivatives displayed strong absorption (505–515 nm with ε_max_ > 6 × 10^4^ M^–1^ cm^–1^) and fluorescence (520–530 nm with ϕ > 60%) bands (see Figure S1 in the Supporting Information). Nevertheless, some structural factors exerting control on the emission behavior under soft (fluorescence) and hard (laser) irradiation conditions could be drawn out (see Table S1): (i) 2,6-dipropargylated BODIPYs emit more efficiently than their monopropargylated counterparts, e.g., **10b** vs **11b**; I the presence of an 8-alkyl group improves the emission efficiency when compared to that of BODIPYs with 8-phenyl groups, e.g. **3** vs **10b**; (iii) B(CN)_2_-BODIPYs emit more efficiently than BF_2_–BODIPYs with the same substitution pattern, e.g., **13** vs **10b**. Consequently, dye **12** fulfilling all these structural constraints achieves a fluorescence quantum yield of 100% and a laser efficiency of 61% (Table S1). The significant enhancement of the emission efficiency correlates with a lowering of the nonradiative probability by reducing (or avoiding) processes such as charge separation within the pyrroles in the asymmetric BODIPYs, small rotational motion of the 8-phenyl moiety and electronic rearrangement within the dipyrrin framework in cyano-BODIPYs (Table S1). In good agreement, the photostability toward prolonged laser irradiation matches the aforementioned photophysical trends becoming as the most photostable those derivatives based on B(CN)_2_-dipropargyl BODIPYs (**12** and **13**) (Figure 5).

**Figure 5:**
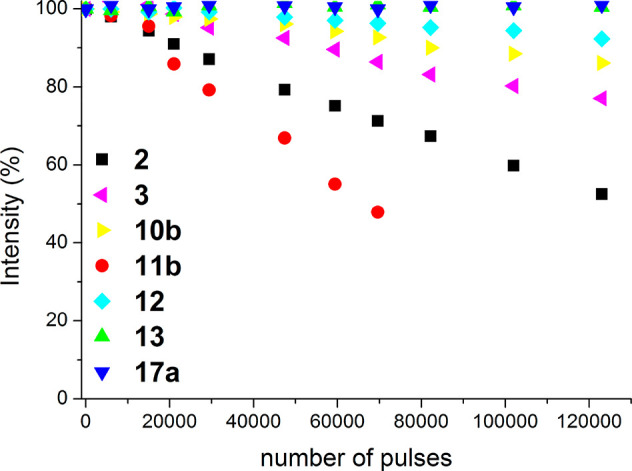
Normalized laser-induced photostability of commercial PM567 (**2**) and its congeners **3**, **10b**, **11b**, **12**, **13**, and **17a** (vide infra). Optically matched solutions were used.

In order to further illustrate the usefulness of the “clickable”^21^ 2,6-dipropargyl BODIPYs, we have conducted two additional studies with implications in, at least, two of the aforementioned relevant research areas involving BODIPYs: (i) the conjugation to biomolecules and (ii) the preparation of light harvesting systems.

Regarding the first topic, we have investigated the click, copper(I)-catalyzed azide–alkyne cycloaddition (CuAAC),^22^ reaction of dialkynyl BODIPY **10b**, with some biologically relevant compounds. Our exploratory experiments on the CuAAC reaction of **10b** were performed with benzyl azide (**14**) in the presence of CuSO_4_ and sodium ascorbate in a glass seal tube (65 °C), leading to bis-1,2,3-triazolyl BODIPY derivative **17a** in 95% yield (3 equiv azido derivatives, 65 °C, Figure 6). We next tested the reactions of **10b** with azido-cholesteryl derivative **15** and 1-ethylene-2-azido α-d-mannopyranosyl glycoside **16**. To our satisfaction, both reacted well with **10b** under the aforementioned experimental conditions, and yielded bis-1,2,3-triazolyl-cholesteryl and bis-1,2,3-triazolyl-d-mannopyranosyl derivatives **17b** and **17c**, in yields of 75% and 92%, respectively.

**Figure 6:**
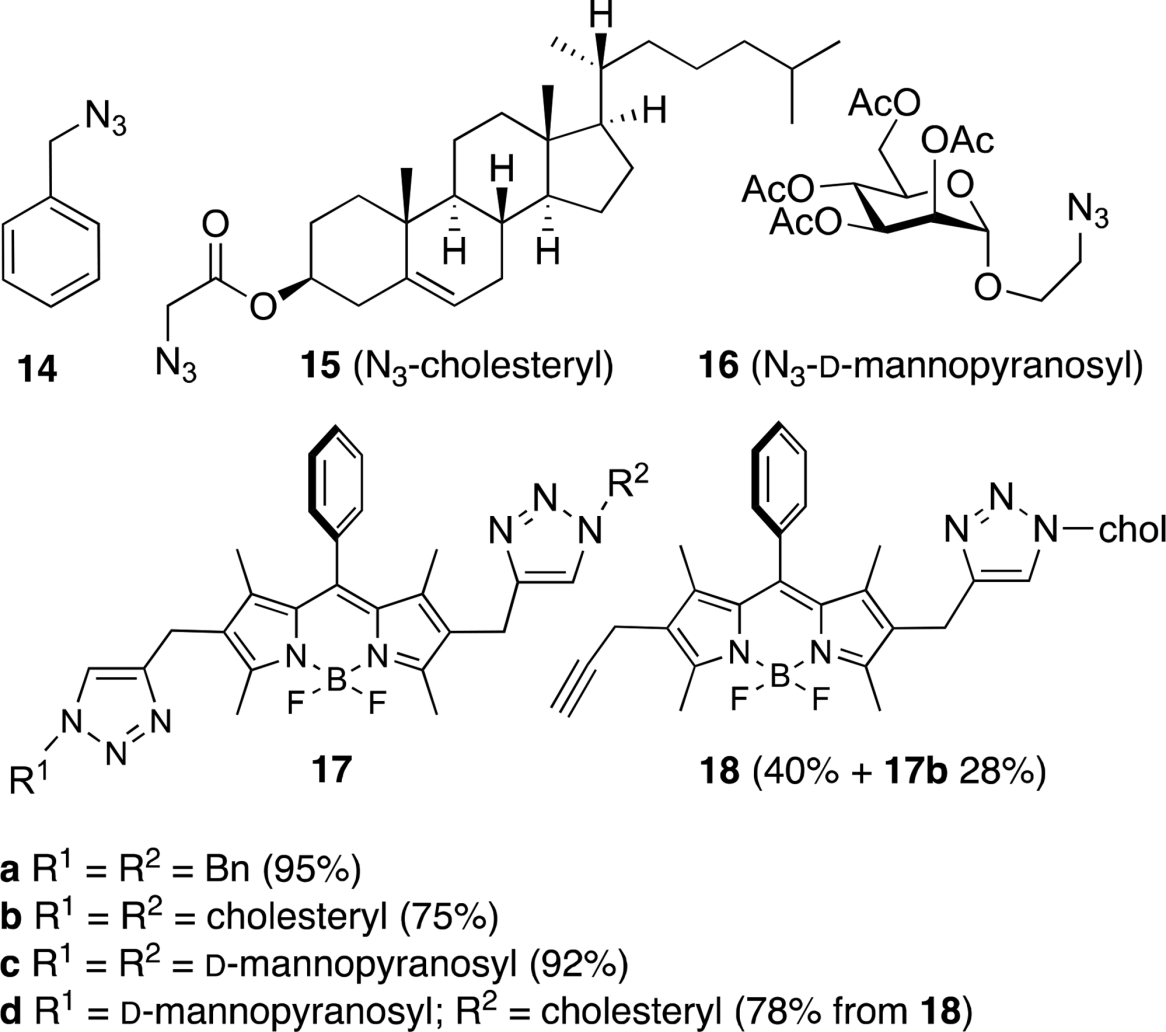
CuAAC-mediated “click” conjugation of BODIPY **10b**. Access to BODIPY-cholesteryl and -dmannopyranosyl adducts. Sodium ascorbate, CuSO_4_, glass seal tube, 65 °C.

The click reaction of **10b** with a limited amount of **15** (0.9 equiv) allowed the preparation monocholesteryl derivative **18** [40% yield, along with **17b** (28% yield)], which, upon a second CuAAC reaction with the azido-mannoside derivative **16** led to the cholesteryl-sugar-BODIPY derivative **17d** in 78% yield (Figure 6). This strategy leads to highly photostable BODIPY-tagged biomolecules with highly efficient emission (fluorescence and laser efficiency of ∼80% and 57%, respectively, for **17b**–**17d** in Table S2 in the Supporting Information), retaining the BODIPY fine spectroscopic signatures after grafting carbohydrate and/or cholesteryl moieties on its core (see Figure S2). In fact, the presence of the triazolyl moieties, rather than the propargyl groups, confers additional photostability to the ensuing BODIPYs (**17a** vs **10b** in Figure 5).

Finally, the modular use of dipropargyl BODIPYs **10a** and **10b**, in conjunction with azido-BODIPYs **20**(23) and **21**, made possible the CuAAC-mediated assembly of isomeric all-BODIPY triads **22a,b** and **23**,^24,25^ where the donor (D) and acceptor (A) roles occupy alternate locations (i.e., A-D-A and D-A-D, respectively, Figure 7). Distyryl BODIPYs **19** and **21** were uneventfully obtained by Knoevenagel condensation of propargyl BODIPY **10b** and azido-BODIPY **20**, with benzaldehyde in DMF. According to that, the CuAAC reactions of dipropargyl BODIPYs **10a** and **10b** with azido-BODIPY **21**, under the reaction conditions depicted in Figure 7, produced all-BODIPY triads **22a** and **22b**, in 89% and 60% yield, respectively, where the donor-role concurs with the central BODIPY unit. Conversely, the CuAAC reaction of dipropargyl-BODIPY **19** with azido-BODIPY **20** led to BODIPY triad **23** (56% yield), where the central BODIPY-unit plays the role of energy acceptor.

**Figure 7:**
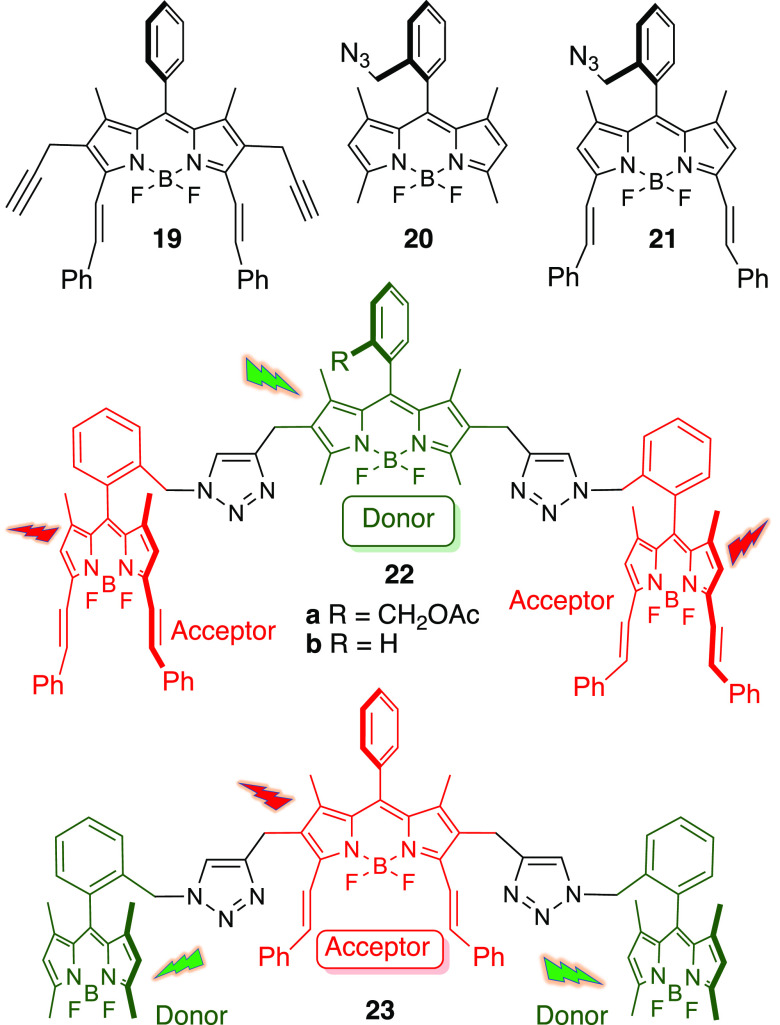
All-BODIPY triads **22a,b** and **23**, obtained by CuAAC mediated modular assembly of 2,6-dipropargyl-BODIPYs **10a**, **10b**, and **19**, and BODIPY azido counterparts **20** and **21**.

This synthetic methodology enables the assessment of the energy donor/acceptor ratio (1/2 in **22b** and 2/1 in **23**) dependence on the photophysical properties of these all-BODIPY multichromophores. The absorption profile features two clearly distinguishable visible bands assigned to each building block (see also the theoretically predicted spectra in Figure S3 in the Supporting Information and the corresponding molecular orbitals, which are placed alternatively in each chromophoric fragment; seee Figure S4 in the Supporting Information); one at shorter wavelengths (500–520 nm), from the alkylated BODIPY acting as energy donor, and other at longer wavelengths (625 nm), from the styryl BODIPY acting as energy acceptor (Figure 8). As it was expected, the short/long wavelength intensity ratio depends on the number of appended units. Moreover, the trademark absorption at 355 nm of BODIPYs bearing 3,5-styryl groups is also recorded. In contrast, the fluorescence profile just displays a single long-wavelength emission (635–645 nm), as result of the ongoing efficient intramolecular excitation energy transfer (EET), regardless of both the excitation wavelength and the architecture of the molecular assembly (Figure 8). The triad **23** bearing two donors and one acceptor displays more efficient emission (up to 65%) than that engaging two acceptor units (**22b**, up to 46%) (see Table S3 in the Supporting Information). Accordingly, under laser radiation at both standard pumping wavelengths of 355 and 532 nm, triad **23** exhibits more efficient red laser emission (centered at 710 nm) than **22b** (25% and 12%, respectively). Nevertheless, they both behave as highly photostable red-emitters retaining their initial laser-induced emission after 2 × 10^5^ UV or visible pump laser pulses (see the Experimental Section in the Supporting Information). Then, the rational design of these triads meets the required criteria to sustain efficient energy transfer in molecular cassettes, such as broadband absorption, because of electronic isolation between the building blocks, and efficient EET (almost no sign of emission from the energy donors), which is attributable to the short donor/acceptor distance imposed by their covalent linkage. These structural factors allow one to achieve bright and long-lasting emission at red spectral window, even under drastic pumping conditions.

**Figure 8:**
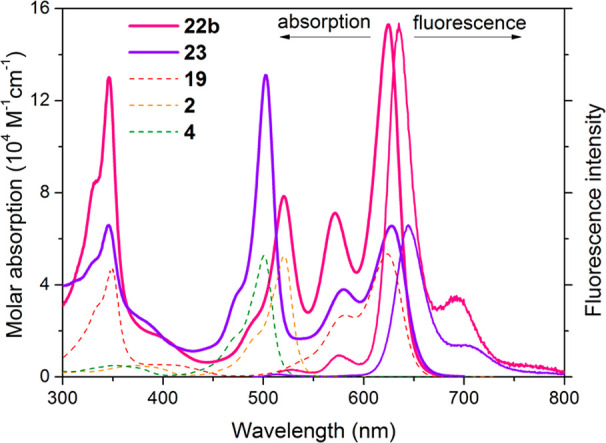
Absorption and normalized fluorescence (dashed, upon excitation 490 nm) spectra of the all-BODIPY based cassettes **22b** and **23** in diluted ethyl acetate solutions (μM). The corresponding absorption spectra (filled) of the free building blocks (**2** in yellow, **4** in green, and **19** in red) are also added.

In summary, we have developed a concise entry to previously unreported 2,6-dipropargyl BODIPYs, which makes use of the Nicholas propargylation reaction. This transformation occurs as an electrophilic aromatic substitution (S_E_Ar) at the dipyrrin framework by the Nicholas’ stabilized dicobalthexacarbonyl propargyl cation. The ensuing 2,6-dipropargyl BODIPYs, which displayed improved photostability compared with the parent PM567 dye, can be engaged in highly efficient click azido-alkyne cycloadditions with either two units of the same (bio)molecule or, in a sequential manner, with two different azido-containing (bio)molecules.^26^ The usefulness of these new derivatives has been demonstrated as fluorescent tags, and as modular components in the assembly of all-BODIPY triads. In addition, the application of the Nicholas propargylation reaction to different BODIPY substrates devoid of methyl substituents is under consideration in our laboratory, and the results will be reported in due course.

## Supporting Information Available

Experimental and computational procedures, copies of NMR spectra, photophysical data, absorption and fluorescence spectra, simulated absorption spectra and molecular orbitals (PDF)
