# Supplementary material for: Access to 2,6-Dipropargylated BODIPYs as “Clickable” Congeners of Pyrromethene-567 Dye: Photostability and Synthetic Versatility
Source: Org Lett. 2021 Aug 17;23(17):6801–6. doi: 10.1021/acs.orglett.1c02380 (PMC8419863; doi:10.1021/acs.orglett.1c02380)
Supplement: Supplementary file 1 — ol1c02380_si_001.pdf [file ol1c02380_si_001.pdf]

Supporting Information

Access to 2,6-Dipropargylated BODIPYs as "Clickable" Congeners of Pyrromethene-567 dye: Photostability and Synthetic Versatility

Clara Uriel,[$] Ana M. Gómez,[$]* Enrique García Martínez de la Hidalga,[§] Jorge Bañuelos,[§]* Inmaculada Garcia-Moreno,[¶] J. Cristobal López,[$]*

[$] *Instituto de Química Orgánica General,* IQOG-CSIC*, Juan de la Cierva 3, 28006, Madrid, Spain.*

[§] *Departamento de Química Física. Universidad del Pais Vasco-EHU, Apartado 644, 48080, Bilbao, Spain.*

[¶] *Instituto de Química-Física "Rocasolano", CSIC, Serrano 119, 28006, Madrid, Spain.*

Table of Contents

1. General Information 2

2. General Procedures 2

3. Compound Characterization 4

4. Photophysical properties 18

5. Lasing properties 19

6. Computational chemistry 20

7. Table S1. Photophysical and lasing properties of representative propargylated BODIPYs at positions 2 and/or 6. 21

8. Table S2. Photophysical and lasing properties of BODIPYs after click conjugation with biomolecules. 22

9. Table S3. Photophysical and lasing properties of the all-BODIPYs based cassettes. 23

10. Figure S1. Absorption and fluorescence spectra of the propargylated BODIPYs 24

11. Figure S2. Absorption and fluorescence spectra of clickable BODIPYs at **2** and **6** positions. 24

12. Figure S3. Theoretically predicted absorption spectra (td b3lyp/6-31g*) of triads **22b** and **23**. 25

13. Figure S4. Theoretically calculated (b3lyp/6-31g*) molecular orbitals involved in the main visible electronic transitions from ground state optimized geometries of the all-BODIPY based cassettes. 26

14. Copies of ^{1}H, ^{13}C {1H}, ^{19}F, ^{11}B NMR Spectra 27

1. General Information

All solvents and reagents were comercial grade and used as received unless stated otherwise. All anhydrous reactions were run under a positive pressure of argon. Reactions were monitored by TLC analysis on Kieselgel 60 F254 (Merk) with UV detection were applicable. Flash column chromatography was carried out using 230–400 mesh silica gel. Melting points were determined with a Stuart SMP20 instrument. ^{1}H, ^{13}C, ^{11}B and ^{19}F NMR spectra were recorded on a BRUKER AVANCE III HD-400 or a JEOL JNM-ECZ400R. Chemical shifts were recorded in parts per million (ppm, δ) relative to the residual solvent peak as internal standard. Coupling constants (*J*) are give in Hz. ^{13}C NMR spectra were proton-decoupled. Optical rotations were measured on a Jasco P2000 polarimeter with $[\alpha]_D^{25}$ values reported in degrees with concentrations expressed in grams per 100 mL.The HRMS experiments were carried out on an Agilent 6500 Accurate Mass Q-TOF LC-MS mass spectrometer and the HRMS data accurate within 5 ppm. 1,3,5,7,8-pentamethyl-4,4-difluoro-4-bora-3a,4a-diaza-*s*-indacene **4**[1], 8-phenyl-1,3,5,7-tetramethyl-4,4-difluoro-4-bora-3a,4a-diaza-*s*-indacene **7b**[2], 1,3,5,7-tetramethyl-4,4-difluoro-4-bora-3a,4a-diaza-*s*-indacene **7c**[3], 2-azidoethyl 2,3,4,6-tetra-*O*-acetyl-α-D-mannopyranoside **16**[4] and 8-(2-azidomethylphenyl)-1,3,5,7-tetramethyl-4,4-difluoro-4-bora-3a,4a-diaza-*s*-indacene **20**[5] were prepared according to previously reported methods.

2. General Procedures

General procedure A. Nicholas reaction of BODIPYs with dicobalt hexacarbonyl propargyl alcohol complex 5

A solution of the corresponding BODIPY (**4**, **7a-c**) and dicobalt hexacarbonyl propargyl alcohol complex **5** (1.1 or 2.2 equiv.) in dry CH_2Cl_2 was cooled at −15 °C, then BF_3OEt_2 (0.5 equiv.) was added. After stirring at −15 °C (1–3 h) the solution was diluted with CH_2Cl_2, washed twice with $NaHCO_3$, dried over $MgSO_4$ and concentrated. The resulting crude was purified by flash chromatography.

---

[1] Shah, M.; Thangaraj, K.; Soong, M.-L.; Wolford, L. T.; Boyer, J. H.; Politzer, I. R.; Pavlopoulos, T. G. *Heteroat.Chem*. **1990**, *1*, 389–399.
[2] Hoogendoorn, S; Blom, A. E. M.; Willems, L. I.; Van der Marel, G. A.; Overkleeft, H. S. *Org. Lett.* **2011**, 13, 20, 5656–5659.
[3] Lundrigan, T.; Baker, A. E. G.; Longobardi, L. E.; Wood, T. E.; Smithen, D. A.; Crawford, S. M.; Cameron, T. S.; Thompson. A. *Org. Lett.* **2012**, *14*, 2158–2161.
[4] Kovalová, A.; Pohl, R.; Vrabel, M. *Organic and Biomolecular Chemistry*, **2018**, *16*, 5960–5964.
[5] del Río, M.; Lobo, F.; López, J. C.; Oliden, A.; Bañuelos, J.; López-Arbeloa, I.; Garcia-Moreno, I.; Gómez, A. M. *J. Org. Chem.,* **2017**, *82*, 1240−1247.

General procedure B. Oxidative iodine-mediated decobaltation

A solution of the corresponding propargylcobalt-BODIPY (1 equiv.) in THF or CH_2Cl_2 was cooled to 0 °C, then solid iodine (3 equiv.) was added. The mixture was stirred under these conditions for 30-90 min, then poured into saturated aqueous $NaHCO_3$ + 10% $Na_2S_2O_3$ solution and partitioned twice with ether or CH_2Cl_2. The combined organic layers were washed once with brine, dried over $MgSO_4$, and filtered, and the solvent was removed in vacuo. The crude material was purified through silicagel column chromatography.

General procedure C. Non-oxidative 1,2-ethylenediamine mediated decobaltation

A solution of the corresponding propargylcobalt-BODIPY (1 equiv.) in THF was treated with 1,2-ethylenediamine (3 equiv.). The reaction mixture was stirred at room temperature until TLC showed complete disparition of the complex, then it was diluted with Et_2O and washed with a 3% HCl solution and brine. The organic layer was dried over $MgSO_4$ and concentrated. The residue was purified by silica column chromatography.

General procedure D. Copper(I)-catalyzed azide-alkyne cycloaddition reaction (CuAAC)

The corresponding azide (**14-16**, **20-21**, 0.9–1.5 equiv./alkyne) and the appropriate alkyne-BODIPY (**10a-b**, **19**, 1 equiv.) in CH_2Cl_2 were added to a solution of sodium ascorbate (3 equiv.) and $CuSO_4$ (1.5 equiv.) in H_2O (CH_2Cl_2/H_2O, 3:1, v/v). The solution was placed in a glass seal tube (65 °C) until no starting materials were left. The reaction mixture was then diluted with CH_2Cl_2 and washed with brine. The organic layer was dried over $MgSO_4$ and concentrated. The residue was purified by silica column chromatography.

General procedure E. Attempted direct propargylation with propargyl trichloroacetimidate.

In a flame dried flask BODIPY **7b**[2] (1.0 equiv.) was dissolved in anhydrous CH_2Cl_2 (0.2 M) followed by the addition of the propargyl trichloroacetimidate[6] (2.0 equiv.). To this solution the corresponding Lewis acid (BF_3Et_2O, $Yb(OTf)_3$, TMSOTf; acid-washed molecular sieves) was added. The resulting mixture was stirred and the reaction was followed by t.l.c.. The reaction mixture was then quenched with the addition of 1M NaOH. The combined organic extracts were dried over $MgSO_4$, filtered and concentrated. The residue was then purified, if necessary, by silical gel chromatography. In most instances the starting BODIPY was recovered unchanged. In one of the ocassions (using $BF_3.OEt_2$ as Lewis acid) the dipropargyl derivative **10b** could be isolated (37% yield).

[6] Wong, V. H. L.; Hor, T. S.; Hii, K. K. *Chem. Commun.* **2013**, *49*, 9272–9274.

3. Compound Characterization

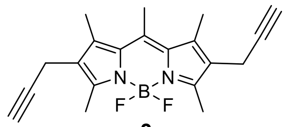

3

1,3,5,7,8-pentamethyl-2,6-di(prop-2-in-1-yl)-4,4-difluoro-4-bora-3a,4a-diaza-s-indacene (3). This compound was prepared following the general procedure B starting from 2,6-dipropargylcobalt-BODIPY **6** (100 mg, 0.11 mmol) in THF (5 mL) and I_2 (42 mg, 0.33 mmol) for 1 h. After work-up, the residue was purified on a silica gel column chromatography (hexane/ethyl acetate, 95:5 to 9:1) to afford **3** as an orange solid (12.3 mg, 33%). Mp >300 °C; ^{1}H NMR ($CDCl_3$, 400 MHz): δ 3.30 (s, 4H), 2.62 (s, 3H), 2.56 (s, 6H), 2.41 (s, 6H), 2.01 (s, 2H). NMR ^{13}C {^{1}H} ($CDCl_3$, 125 MHz): δ 152.3, 141.5, 137.5, 131.9, 125.2, 81.4, 68.6, 17.2, 14.8, 13.8, 12.7; HRMS (ESI/Q-TOF) m/z: $[M+H]^+$ Calcd for $C_{20}H_{22}BF_2N_2$ 339.1842; Found: 339.1833.

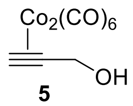

5

Dicobalt hexacarbonyl propargyl alcohol complex (5)

To a solution of propargylic alcohol (0.2 mL, 3.6 mmol, 1 equiv.) in dry CH_2Cl_2 (25 mL) was added dicobalt octacarbonyl (1.47 g, 4.32 mmol, 1.2 equiv.). After stirring 1 h, the crude was concentrated on a rotary evaporator and the residue was purified by flash chromatography (hexane/ethyl acetate, 9:1) to give **5**[7] as a brown solid (1.17g, 95%); Mp 50-52 °C. ^{1}H NMR ($CDCl_3$, 300 MHz): δ 6.07 (s, 1H), 4.80 (d, *J* = 6.0 Hz, 2H), 1.79 (t, *J* = 6.0 Hz, 1H).

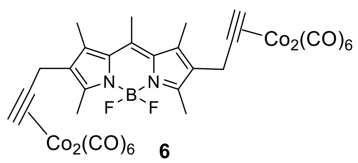

6

Bis $Co_2(CO)_6$ 1,3,5,7,8-pentamethyl-2,6-di(prop-2-in-1-yl)-4,4-difluoro-4-bora-3a,4a-diaza-s-indacene (6). BODIPY **4** (200 mg, 0.76 mmol) was reacted with dicobalt hexacarbonyl propargyl alcohol complex **5** (571 mg, 1.67 mmol) and BF_3OEt_2 (47 μL, 0.38 mmol) following the general

[7] Wells, S. M.; Widen, J. C.; Harki, D. A.; Brummond, K. M. *Org. Lett.* **2016,** *18*, 4566–4569.

procedure A (30 min). The residue was purified by flash silicagel chromatography (toluene) to give derivative **6** as a brown solid (692 mg, 73%). Mp >300 °C; ^{1}H NMR ($CDCl_3$, 400 MHz): δ 5.99 (s, 2H), 3.94 (s, 4H), 2.63 (s, 3H), 2.57 (s, 6H), 2.43 (s, 6H). ^{13}C {^{1}H} NMR ($CDCl_3$, 125 MHz): δ 199.6, 152.4, 141.5, 137.1, 132.0, 129.9, 95.7, 73.3, 28.9, 17.2, 15.3, 12.8. HRMS (ESI/Q-TOF) m/z: $[M+H]^+$ Calcd for $C_{32}H_{22}BCo_4F_2N_2O_{12}$ 910.8562; Found 910.8591.

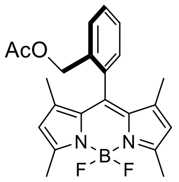

7a

8-(2-acetoxymethyl-phenyl)-1,3,5,7-tetramethyl-4,4-difluoro-4-bora- 3a,4a-diaza-s-indacene (7a). To a stirred solution of 8-(2-hydroxymethyl-phenyl)-1,3,5,7-tetramethyl-4,4-difluoro-4-bora- 3a,4a-diaza-*s*-indacene[5] (200 mg, 0.56 mmol) in pyridine (8 mL) was added acetic anhydride (2 mL). After stirring 1 h, the crude was concentrated and the residue was purified by flash chromatography (hexane/ethyl acetate, 9:1) to give **7a** (219 mg, 98%).^{1}H NMR ($CDCl_3$, 400 MHz): δ 7.50-7.40 (m, 3H), 7.23-7.20 (m, 4H), 5.96 (s, 2H), 5.00 (s, 2H), 2.53 (s, 6H), 1.91 (s, 3H), 1.34 (s, 6H). ^{13}C {^{1}H} NMR ($CDCl_3$, 125MHz): δ 170.4, 155.8, 142.8, 139.1, 134.1, 133.7, 131.0, 129.5, 129.2, 129.1, 128.5, 121.3, 63.7, 20.4, 14.6, 13.8. HRMS (ESI/Q-TOF) m/z: $[M+H]^+$ Calcd for $C_{22}H_{24}BF_2N_2O_2$ 397.1898; Found 397.1873.

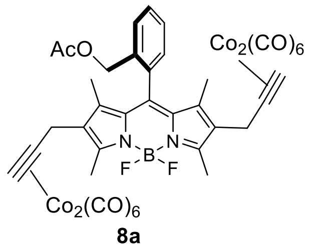

8a

Bis-$Co_2(CO)_6$ 8-(2-acetoxymethyl-phenyl)-1,3,5,7-tetramethyl-2,6-di(prop-2-in-1-yl)-4,4-difluoro-4-bora- 3a,4a-diaza-s-indacene (8a). Following the general procedure A, BODIPY **7a** (100 mg, 0.25 mmol) was reacted with dicobalt hexacarbonyl propargyl alcohol complex **5** (190 mg, 0.55 mmol) and BF_3OEt_2 (15.4 μL, 0.125 mmol) (1 h). The residue was purified by flash silicagel chromatography (hexane/ethyl acetate, 98 :2 to 95 :5) to give derivative **8a** as brown solid (256 mg, 98%). Mp 136-140 °C; ^{1}H NMR ($CDCl_3$, 400 MHz): δ 7.51-7-46 (m, 3H), 7.12-7.10 (m, 1H), 5.96 (s, 2H), 4.96 (s, 2H), 3.84 (s, 4H), 2.62 (s, 6H), 2.03 (s, 3H), 1.37 (s, 6H). ^{13}C {^{1}H} NMR

(CDCl$_3$, 125MHz): δ 199.6, 170.5, 154.7, 139.3, 139.0, 134.2, 133.9, 131.1, 130.7, 130.4, 129.8, 129.2, 129.0, 128.5, 95.3, 73.3, 63.2, 28.8, 20.8, 13.0, 11.9; HRMS (ESI/Q-TOF) m/z: $[M+NH_4]^+$ Calcd for $C_{40}H_{31}BCo_4F_2N_3O_{14}$ 1061.9197; Found: 1061.9191.

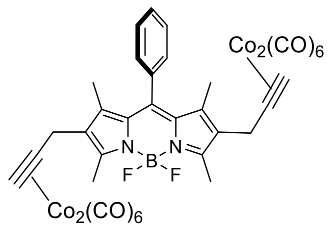

8b

Bis-$Co_2(CO)_6$ 8-phenyl-1,3,5,7-tetramethyl-2,6-di(prop-2-in-1-yl)-4,4-difluoro-4-bora-3a,4a-diaza-s-indacene (8b) BODIPY **7b**[2] (250 mg, 0.77 mmol) was reacted with dicobalt hexacarbonyl propargyl alcohol complex **5** (579 mg, 1.70 mmol) and BF_3OEt_2 (47 μL, 0.38 mmol) following the general procedure A (90 min). The residue was purified by flash chromatography (hexane/ethyl acetate 95:5) to give derivative **8b** as a brown solid (726 mg, 97%). Mp >300 °C; ^{1}H NMR (CDCl$_3$, 400 MHz): δ 7.51-7.50 (m, 3H), 7.19-7.17 (m, 2H), 5.95 (s, 2H), 3.83 (s, 4H), 2.62 (s, 6H), 1.37 (s, 6H). ^{13}C {^{1}H} NMR (CDCl$_3$, 125 MHz): δ 199.6, 154.2, 142.0, 139.3, 135.4, 131.1, 130.2, 129.5, 129.2, 128.0, 95.5, 73.3, 28.8, 13.0, 12.4. HRMS (ESI/Q-TOF) m/z: $[M+H]^+$ Calcd for $C_{37}H_{23}BCo_4FN_2O_{12}$ 952.8657; Found 952.8682.

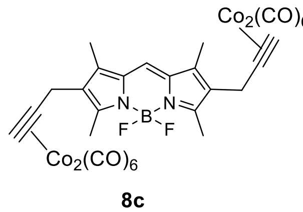

8c

***Bis $Co_2(CO)_6$ 1,3,5,7-tetramethyl-2-(prop-2,6-in-1-yl)-4,4-difluoro-4-bora-3a,4a-diaza-s-indacene* (8c).** Following the general procedure A, BODIPY **7c**[3] (50 mg, 0.2 mmol) was reacted with dicobalt hexacarbonyl propargyl alcohol complex **5** (152 mg, 0.44 mmol) and BF_3OEt_2 (12.3 μL, 0.1 mmol) (45 min). The residue was purified by flash silicagel chromatography (hexane/ethyl acetate 95 :5) to give derivative **8c** as a brown solid (147 mg, 82%). Mp >300°C; ^{1}H NMR (CDCl$_3$, 400 MHz): δ 7.03 (s, 1H), 6.01 (s, 2H), 3.92 (s, 4H), 2.58 (s, 6H), 2.27 (s, 6H). ^{13}C {^{1}H} NMR (CDCl$_3$, 125 MHz): δ 199.6, 155.3, 137.5, 132.5, 129.1, 120.2, 95.3, 73.3, 29.1, 13.0, 9.9. HRMS (ESI/Q-TOF) m/z: $[M+H]^+$ Calcd for $C_{31}H_{20}BCo_4F_2N_2O_{12}$ 896.8405; Found 896.8424.

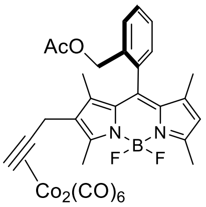

9a

Co₂(CO)₆ 8-(2-acetoxymethyl-phenyl)-1,3,5,7-tetramethyl-2-(prop-2-in-1-yl)-4,4-difluoro-4-bora- 3a,4a-diaza-s-indacene (9a). This compound was prepared following de general procedure A starting from BODIPY **7a** (198 mg, 0.5 mmol), dicobalt hexacarbonyl propargyl alcohol complex **5** (188 mg, 0.55 mmol) and BF_3OEt_2 (31 µL, 0.25 mmol) (1 h). After work-up, the residue was purified on a silica gel column chromatography (hexane/ethyl acetate, 98:2 to 95:5) to afford BODIPY **9a** (101 mg, 28%) along with BODIPY **8a** (100mg, 19%). Compound **9a**: ^{1}H NMR ($CDCl_3$, 400 MHz): δ 7.52-7.44 (m, 3H), 7.19-7.18 (m, 1H), 5.98 (s, 1H), 5,97 (s, 1H), 5.01 (d, *J* = 13.4Hz, 1H), 4.97 (d, *J* = 13.3 Hz, 1H), 3.85 (s, 2H), 2.62 (s, 3H), 2.56 (s, 3H), 1.98 (s, 3H), 1.38 (s, 3H), 1.35 (s, 3H). ^{13}C {^{1}H} NMR ($CDCl_3$, 125MHz): δ 199.6, 170.6, 156.7, 154.0, 143.5, 139.2, 138.6, 134.1, 134.04, 131.5, 130.3, 130.1, 129.7, 129.3, 129.0, 128.6, 121.7, 95.5, 73.4, 63.5, 28.8, 20.7, 14.8, 14.0, 13.0, 11.9. HRMS (ESI/Q-TOF) m/z: $[M+H]^+$ Calcd for $C_{31}H_{26}BCo_2F_2N_2O_8$ 721.0414; Found 721.0403; $[M+Na]^+$ Calcd for $C_{31}H_{25}BCo_2F_2N_2NaO_8$ 743.0234; Found 743.0219.

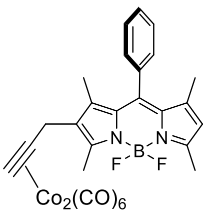

9b

$Co_2(CO)_6$ 8-phenyl-1,3,5,7-tetramethyl-2-prop-2-in-1-yl-4,4-difluoro-4-bora-3a,4a-diaza-s-indacene (9b). Following the general procedure A, BODIPY **7b**[2] (250 mg, 0.77 mmol) was reacted with dicobalt hexacarbonyl propargyl alcohol complex **5** (289 mg, 0.85 mmol) and BF_3OEt_2 (47 µL, 0.38 mmol) (1 h). The residue was purified by flash silicagel chromatography (toluene) to give BODIPY **9b** as orange solid (140 mg, 28%) along with BODIPY **8b** (150 mg, 20%). Compound **9b**: Mp 151-154 °C; ^{1}H NMR ($CDCl_3$, 400 MHz): δ 7.50-7.49 (m, 3H), 7.24-7.22 (m, 2H), 5.98 (s, 1H), 5.96 (s, 1H), 3.84 (s, 2H), 2.62 (s, 3H), 2.55 (s, 3H), 1.38 (s, 3H), 1.36 (s, 3H). ^{13}C {^{1}H} NMR ($CDCl_3$, 125 MHz): δ 199.6, 156.2, 153.6, 143.7, 141.9, 139.0, 135.3, 131.8, 130.9, 129.9, 129.4,

129.2, 128.0, 121.6, 95.6, 73.4, 28.8, 14.7, 14.5, 13.0, 12.4. HRMS (ESI/Q-TOF) m/z: $[M+H]^+$ Calcd for $C_{28}H_{22}BCo_2F_2N_2O_6$ 649.0202; Found 649.0216.

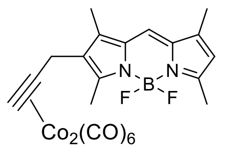

9c

$Co_2(CO)_6$ 1,3,5,7-tetramethyl-2-(prop-2-in-1-yl)-4,4-difluoro-4-bora-3a,4a-diazo-s-indaceno (9c). BODIPY **7c**[3] (50 mg, 0.2 mmol) was reacted with dicobalt hexacarbonyl propargyl alcohol complex **5** (74 mg, 0.22 mmol) and BF_3OEt_2 (12.3 μL, 0.1 mmol) following the general procedure A (45 min). The residue was purified by flash chromatography (toluene) to give derivative **9c** as a brown solid (57.2 mg, 50%). Mp >300 °C; ^1H NMR ($CDCl_3$, 400 MHz): δ 7.04 (s, 1H), 6.05 (s, 1H), 6.02 (s, 1H), 3.93 (s, 2H), 2.58 (s, 3H), 2.53 (s, 3H), 2.27 (s, 3H), 2.24 (s, 3H). $^{13}C\{^1H\}$ NMR ($CDCl_3$, 125 MHz): δ 199.6, 157.7, 154.5, 141.5, 137.2, 133.9, 132.4, 128.8, 120.2, 119.4, 95.5, 73.3, 29.2, 14.8, 13.0, 11.4, 9.9. HRMS (ESI/Q-TOF) m/z: $[M+H]^+$ Calcd for $C_{22}H_{18}BCo_2F_2N_2O_6$ 572.9888; Found 572.9891.

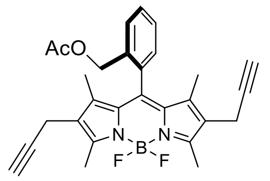

10a

8-(2-acetoxymethyl-phenyl)-1,3,5,7-tetramethyl-2,6-di(prop-2-in-1-yl)-4,4-difluoro)-4-bora-3a,4a-diaza-s-indacene *(10a)*. This compound was prepared following the general procedure B starting from cobalt-complex **8a** (115 mg, 0.11 mmol) in THF (5 mL) and I_2 (42 mg, 0.33 mmol) (90 min). After work-up, the residue was purified on a silica gel column chromatography (hexane/ethyl acetate, 9:1) to afford **10a** as an orange solid (48.8 mg, 94%). Mp 135-138 °C; ^1H NMR ($CDCl_3$, 400 MHz): δ 7.52-7.47 (m, 3H), 7.26-7.24 (m, 1H), 5.00 (s, 2H), 3.20 (d, J = 2.8 Hz, 4H), 2,60 (s, 6H), 1.95 (t, J = 2.7 Hz, 2H), 1.91 (s, 3H), 1.33 (s, 6H). $^{13}C\{^1H\}$ NMR ($CDCl_3$, 125MHz): δ 170.7, 154.6, 139.3, 139.1, 134.6, 133.9, 130.6, 129.8, 129.6, 129.4, 128.9, 125.7, 81.1, 68.6, 64.0, 20.7, 13.7, 12.9, 11.6. HRMS (ESI/Q-TOF) m/z: $[M+H]^+$ Calcd for $C_{28}H_{28}BF_2N_2O_2$ 473.2211; Found 473.2235; $[M+NH_4]^+$ Calcd for $C_{28}H_{31}BF_2N_3O_2$ 491.2506; Found: 491.2527.

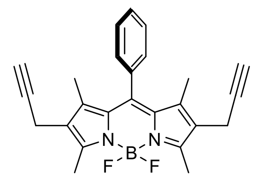

10b

8-phenyl-1,3,5,7-tetramethyl-2,6-di(prop-2-in-1-yl)-4,4-difluoro-4-bora-3a,4a-diaza-s-indacene (10b). This compound was prepared following the general procedure B starting from cobalt-complex **8b** (150 mg, 0.15 mmol) in CH_2Cl_2 (5 mL) and I_2 (57 mg, 0.45 mmol) (1 h). After work-up, the residue was purified on a silica gel column chromatography (hexane/ethyl acetate, 95:5) to afford **10b** as an orange solid (56 mg, 94%). Mp 178-181 °C; ^{1}H NMR ($CDCl_3$, 400 MHz): δ 7.50-7.48 (m, 3H), 7.29-7.26 (m, 2H), 3.20 (d, *J* = 2,8 Hz, 4H), 2.60 (s, 6H), 1.96 (t, *J* = 2,7 Hz, 2H), 1.35 (s, 6H).^{13}C {1H} NMR ($CDCl_3$, 125MHz): δ 154.1, 141.7, 139.6, 135.3, 130.9, 129.3, 129.2, 128.2, 125.5, 81.1, 77.4, 68.5, 13.7, 12.8, 12.0. HRMS (ESI/Q-TOF) m/z: $[M+H]^+$ Calcd for $C_{25}H_{24}BF_2N_2$ 401.2000; Found 401.2008.

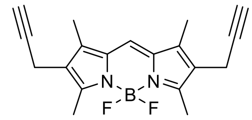

10c

1,3,5,7-tetramethyl-2,6-di(prop-2-in-1-yl)-4,4-difluoro-4-bora-3a,4a-diaza-s-indacene (10c). BODIPY **8c** (100 mg, 0.11 mmol) was reacted with 1,2-ethylenediamine (22 μL, 0.33 mmol) in THF (5 mL) following the general procedure C (45 min). The residue was purified by flash silicagel chromatography (hexane-ethyl acetate 9:1) to give derivative **10c** (26 mg, 73%). Mp 205-208 °C; ^{1}H NMR ($CDCl_3$, 400 MHz): δ 7.03 (s, 1H), 3.29 (d, *J* = 2.8 Hz, 4H), 2.56 (s, 6H), 2.24 (s, 6H), 2.02 (t, *J* = 2.7 Hz, 2H); ^{13}C {^{1}H} NMR ($CDCl_3$, 125 MHz): δ 155.2, 137.9, 132.6, 124.2, 120.0, 81.0, 68.8, 13.9, 12.9, 9.8. HRMS (ESI/Q-TOF) m/z: $[M+H]^+$ Calcd for $C_{19}H_{20}BF_2N_2$ 325.1686; Found 325.1693.

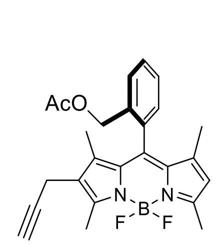

11a

8-(2-acetoxymethyl-phenyl)-1,3,5,7-tetramethyl-2-(prop-2-in-1-yl)-4,4-difluoro-4-bora- 3a,4a-diaza-s-indacene (11a). This compound was prepared according to the general procedure B from BODIPY **9a** (100 mg, 0.14 mmol) and I_2 (53 mg, 0.42 mmol) in THF (4 mL). The residue was purified by flash silicagel chromatography (hexane-ethyl acetate 9:1) to give derivative **11a** (57.1 mg, 94%). Mp 134-137 °C; ^{1}H NMR ($CDCl_3$, 400 MHz): δ 7.51-7.44 (m, 3H), 7.26-7.24 (m, 1H), 5.98 (s, 1H), 5.01 (s, 2H), 3.20 (d, *J* = 2.8 Hz, 2H), 2.60 (s, 3H), 2.55 (s, 3H), 1.95 (t, *J* = 2.8 Hz, 1H), 1.92 (s, 3H), 1.34 (s, 6H). ^{13}C {^{1}H} ($CDCl_3$, 125 MHz): δ 170.6, 156.2, 154.4, 143.1, 139.2, 134.5, 133.9, 131.2, 130.5, 129.7, 129.4, 129.3, 128.8, 125.6, 121.6, 81.1, 68.6, 63.9, 20.6, 14.8, 14.0, 13.7, 12.9, 11.5. HRMS (ESI/Q-TOF) m/z: $[M+H]^+$ Calcd for $C_{25}H_{26}BF_2N_2O_2$ 435.2054; Found 435.2054.

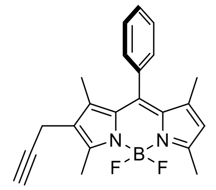

11b

8-phenyl-1,3,5,7-tetramethyl-2-(prop-2-in-1-yl)-4,4-difluoro-4-bora-3a,4a-diaza-s-indacene (11b). BODIPY **9b** (100 mg, 0.15 mmol) was reacted with I_2 (57 mg, 0.45 mmol) in CH_2Cl_2 (5 mL) following the general procedure B (30 min). The residue was purified by flash silicagel chromatography (hexane-ethyl acetate 95:5) to give derivative **11b** as an orange solid (39 mg, 72%). Mp 175-180 °C; ^{1}H NMR ($CDCl_3$, 400 MHz): δ 7.50-7.48 (m, 3H), 7.27-7.18 (m, 2H), 5.96 (s, 1H), 3.18 (d, *J* = 2.7 Hz, 1H), 2.58 (s, 3H), 2.54 (s, 3H), 1.94 (t, *J* = 2.8 Hz, 1H), 1.34 (s, 6H). ^{13}C {^{1}H} NMR ($CDCl_3$, 125MHz): δ 155.8, 154.0, 143.4, 141.8, 139.5, 135.2, 131.6, 130.9, 129.3, 129.1, 128.2, 125.4, 121.4, 81.2, 68.5, 14.7, 14.5, 13.7, 12.8, 12.0. HRMS (ESI/Q-TOF) m/z: $[M+H]^+$ Calcd for $C_{22}H_{22}BF_2N_2$ 363.1843; Found 363.1852. $[M+Na]^+$ Calcd for C_{22} H_{21} B F_2 N_2 Na 385.1662; Found 385.1667.

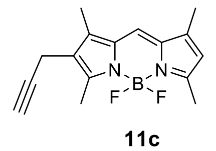

11c

1,3,5,7-tetramethyl-2-(prop-2-in-1-yl)-4,4-difluoro-4-bora-3a,4a-diaza-s-indacene (11c). BODIPY **9c** (100 mg, 0.175 mmol) was reacted with 1,2-ethylenediamine (35 μL, 0.52 mmol) in THF (5 mL) following the general procedure C (30 min). The residue was purified by flash silicagel chromatography (hexane-ethyl acetate 95:5) to give derivative **9c** as an orange solid (30.5 mg, 61%). Mp 163-166°C; ^{1}H NMR ($CDCl_3$, 400 MHz): δ 7.02 (s, 1H), 6.03 (s, 1H), 3.27 (d, *J* = 2.7 Hz,

2H), 2.55 (s, 3H), 2.52 (s, 3H), 2.22 (s 3H), 2.21 (s, 3H), 2.02 (t, *J* = 2.7 Hz, 1H). ^{13}C {^{1}H} NMR ($CDCl_3$, 125MHz): δ 156.9, 154.9, 141.4, 137.8, 133.6, 132.4, 124.1, 120.1, 119.1, 81.0, 68.8, 14.8, 13.9, 12.8, 11.3, 9.7. HRMS (ESI/Q-TOF) m/z: $[M+H]^+$ Calcd for $C_{16}H_{18}BF_2N_2$ 287.1529; Found 287.1520.

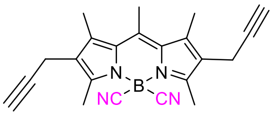

12

1,3,5,7,8-pentamethyl-2,6-di(prop-2-in-1-yl)-4,4-dicyan-4-bora-3a,4a-diaza-s-indacene (12). To a stirred solution of **3** (100 mg, 0.296 mmol) in CH_2Cl_2 (5 mL) at 0 °C was added BF_3OEt_2 (8 μL, 0.06 mmol) and trimethylsilyl cyanide TMSCN (91 μL, 0.89 mmol). The mixture was stirred for 1 h, then poured into saturated aqueous $NaHCO_3$ solution and extracted twice with CH_2Cl_2. The combined organic layers were dried over $MgSO_4$, and filtered, and the solvent was removed in vacuo. The crude material was purified through silicagel column chromatography (hexane-ethyl acetate 9:1 to 8:2) to give derivative **12** as orange solid (104 mg, quant.); ^{1}H NMR ($CDCl_3$, 400 MHz): 3.36 (d, *J* = 2.7 Hz, 4H), 2.73 (s, 6H), 2.69 (s, 3H), 2.47 (s, 6H), 2.04 (t, *J* = 2.7 Hz, 2H). ^{13}C {^{1}H} NMR (125 MHz, $CDCl_3$) δ 152.8, 142.6, 138.9, 130.4, 126.9, 126.6 (q, *J* = 74.0 Hz), 80.5, 69.3, 17.5, 15.1, 14.0, 13.8, 13.7. HRMS (ESI/Q-TOF) m/z: $[M+H]^+$ Calcd for $C_{22}H_{21}BN_4$ 353.1936; Found 353.1925.

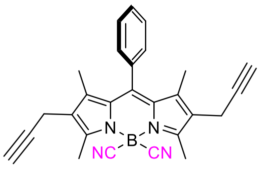

13

8-phenyl-1,3,5,7-tetramethyl-2,6-di(prop-2-in-1-yl)-4,4-dicyan-4-bora-3a,4a-diaza-s-indacene (13). To a stirred solution of **10b** (55 mg, 0.137 mmol) in CH_2Cl_2 (5 mL) at 0 °C was added BF_3OEt_2 (4 μL, 0.03 mmol) and trimethylsilyl cyanide TMSCN (42 μL, 0.41 mmol). The mixture was stirred for 90 min, then poured into saturated aqueous $NaHCO_3$ solution and extracted twice with CH_2Cl_2. The combined organic layers were dried over $MgSO_4$, and filtered, and the solvent was removed in vacuo. The crude material was purified through silicagel column chromatography (hexane-ethyl acetate 9:1) to give derivative **13** as an orange solid (48.2 mg, 85%). Mp 175-180 °C; ^{1}H NMR ($CDCl_3$, 400 MHz): ^{1}H NMR ($CDCl_3$, 400 MHz): 7.55-7.52 (m, 3H), 7.29-7.27 (m, 2H), 3.25 (d, *J* = 2.7 Hz, 4H), 2.77 (s, 6H), 2.00 (t, *J* = 2.7 Hz, 2H), 1.39 (s, 6H). **^{13}**C {^{1}H} NMR ($CDCl_3$,

125MHz): 154.6, 142.6, 141.0, 134.3, 129.7, 129.6, 129.4, 127.9, 127.7, 127.2, 127.1, 127.0, 126.3, 125.5, 80.4, 69.2, 13.9, 13.8, 12.3. ^{11}B NMR (128 MHz, $CDCl_3$) δ-16.80. HRMS (ESI/Q-TOF) m/z: $[M+H]^+$ Calcd for $C_{27}H_{24}BN_4$ 415.2093; Found 415.2104; $[M+Na]^+$ Calcd for $C_{27}H_{23}B$ N_4Na 437.1913; Found 437.1916.

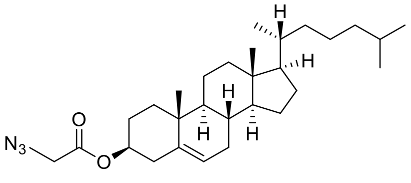

15

Cholester-3β-yl 2-Azidoacetate (15). To a stirred solution of Cholesterol (300 mg, 0.78 mmol), 2-azidoacetic acid (63 μL, 0.85 mmol) and N- (3-dimethylaminopropyl)-N'-ethylcarbodiimide EDC (301 mg, 1.01 mmol) in dry CH_2Cl_2 (5 mL) was added 4-dimethylaminopyridine (9.5 mg, 0.078 mmol). The mixture was stirred at room temperature for 1 h, then diluted with CH_2Cl_2 (25 mL) and poured into brine (15 mL). The organic layer was dried over $MgSO_4$, filtered and the solvent was removed in vacuo. The crude material was purified through silicagel column chromatography (hexane-ethyl acetate 9:1) to give derivative **15** (335 mg, 91%), the data are in agreement with literature values[8]. Mp = 122-125 °C; ^{1}H NMR ($CDCl_3$, 300 MHz): δ 5.38 (d, *J* = 6.7 Hz, 2H), 4.74-4.65 (m, 1H), 3.82 (s, 2H), 2.35 (m, 2H), 2.02-1.07 (m, 25H), 1.01 (s, 3H), 0.90 (d, *J* = 6.7 Hz, 2H), 0.85 (d, *J* = 6.7 Hz, 2H), 0.67 (s, 3H); ^{13}C {^{1}H} NMR ($CDCl_3$, 125MHz): 167.7, 139.2, 123.2, 75.9, 56.7, 56.2, 50.5, 50.1, 42.4, 39.8, 39.6, 38.1, 37.0, 36.6, 36.3, 35.9, 32.0, 31.9, 28.3, 28.1, 27.8, 24.4, 24.0, 22.9, 22.7, 21.1, 19.3, 18.8, 11.9.

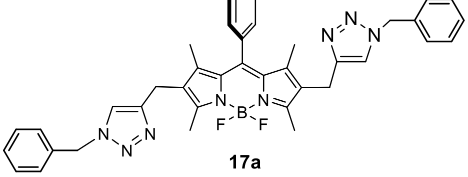

17a

Compound 17a. Following the general procedure D, BODIPY **10b** (52 mg, 0.13 mmol) and benzyl azide (38 μL, 0.31 mmol) in CH_2Cl_2 (6 mL) were added to a solution of sodium ascorbate (78 mg, 0.39 mmol) and $CuSO_4$ (48 mg, 0.19 mmol) in H_2O (2 mL). The solution was placed in a glass seal tube (65 °C) for 1 h. The residue was purified by silica column chromatography (hexane/ethyl acetate: 6:4) to afford BODIPY **17a** (82 mg, 95%). Mp 118-121 °C; ^{1}H NMR ($CDCl_3$, 400 MHz): δ

[8] Pospieszny, T. *Helv. Chim. Acta*, **2015**, *98*, 1337–1350

7.46-7.34 (m, 9H), 7.22-7.20 (m, 6H), 6.99 (s, 2H), 5.44 (s, 4H), 3.75 (s, 4H), 2.48 (s, 6H), 1.26 (s, 6H). ^{13}C {^1H} NMR ($CDCl_3$, 125MHz): δ 154.4, 147.0, 141.5, 140.1, 135.2, 134.7, 131.1, 129.1, 129.2, 128.9, 128.0, 127.7, 121.2, 54.4, 20.8, 12.9, 12.2. HRMS (ESI/Q-TOF) m/z: $[M+Na]^+$ Calcd for $C_{39}H_{37}BF_2N_8Na$ 689.3101; Found 689.3111.

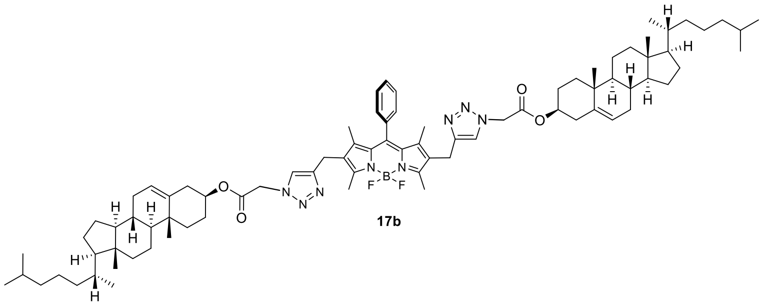

17b

Compound 17b. Following the general procedure D, BODIPY **10b** (30 mg, 0.075 mmol) and azido-cholesteryl derivative **15** (105 mg, 0.22 mmol) in CH_2Cl_2 (6 mL) were added to a solution of sodium ascorbate (45 mg, 0.22 mmol) and $CuSO_4$ (28 mg, 0.11 mmol) in H_2O (2 mL). The solution was placed in a glass seal tube (65 °C) for 6 h. The residue was purified by silica column chromatography (hexane/ethyl acetate: 7 :3) to afford BODIPY **17b** (42 mg, 42%). Mp 206-209 °C ; $[\alpha]_D^{21} = -108.6$ (*c* 0.05, $CHCl_3$); ^1H NMR ($CDCl_3$, 400 MHz): δ 7.47-7.45 (m, 3H), 7.27-7.25 (m, 2H), 7.20 (m, 2H), 5.37-5.36 (m, 2H), 5.04 (s, 4H), 4.71-4.64 (m, 2H), 3.79 (s, 4H), 2.53 (s, 6H), 2.31-2.30 (m, 4H), 2.03-1.94 (m, 4H), 1.88- 1.80 (m, 6H), 1.59-1.43 (m, 14H), 1.36-1.29 (m, 12H), 1.24-1.06 (m, 16H), 1.02-0.95 (m, 12H), 0.91 (d, *J* = 6.7 Hz, 1H), 0.86 (d, *J* = 6.7 Hz, 12H) 0.67 (s, 6H). ^{13}C {^1H} NMR ($CDCl_3$, 125MHz): 165.8, 154.5, 147.2, 141.5, 140.0, 139.1, 135.3, 131.1, 129.3, 129.2, 128.2, 127.8, 123.4, 122.5, 76.5, 56.8, 56.3, 51.2, 50.1, 42.4, 39.8, 39.6, 38.0, 36.9, 36.6, 36.3, 35.9, 32.0, 31.9, 28.3, 28.1, 27.7, 24.4, 24.0, 23.0, 22.7, 21.1, 20.9, 19.4, 18.8, 12.9, 12.2, 12.0. HRMS (ESI/Q-TOF) m/z: $[M+Na]^+$ Calcd for $C_{83}H_{117}BF_2N_8NaO_4$ 1361.9157; Found 1361.9172.

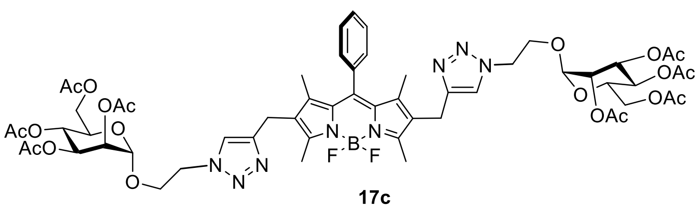

17c

Compound 17c. Following the general procedure D, BODIPY **10b** (30 mg, 0.075 mmol) and 1-ethylene-2-azido-α-D-mannopyrannosyl glycoside (94 mg, 0.22 mmol) in CH_2Cl_2 (6 mL) were added to a solution of sodium ascorbate (45 mg, 0.22 mmol) and CuSO4 (28 mg, 0.11 mmol) in H_2O (2 mL). The solution was placed in a glass seal tube (65 °C) for 24 h. The residue was purified

by silica column chromatography (hexane/ethyl acetate: 1/1 to ethyl acetate) to afford BODIPY **17c** (85 mg, 92%). $[\alpha]_D^{21}$ = −66.2 (*c* 0.08, $CHCl_3$); ^1H NMR ($CDCl_3$, 400 MHz): δ 7.44-7.42 (m, 3H), 7.28-7.26 (m, 4H), 5.21 (t, *J* = 9.6 Hz, 2H), 5.14-5.10 (m, 4H), 4.75 (bs, 2H), 4.49 (bs, 4H), 4.17 (dd, *J* = 12,4, 5,0 Hz, 2H), 4.09-4.04 (m, 2H), 4.01-3.98 (m, 2H), 3.84-3.79 (m, 2H), 3.74 (m, 2H), 3.57-3.51 (m, 2H), 2.49 (s, 6H), 2.08 (s, 6H), 2.04 (s, 6H), 2.00 (s, 6H), 1.91 (s, 6H), 1.31 (s, 6H). $^{13}C\{^1H\}$ NMR ($CDCl_3$, 125 MHz): 170.6, 170.1, 169.8, 169.7, 154.2, 141.4, 139.8, 135.4, 131.0, 129.2, 129.0, 128.2, 127.8, 97.5, 69.2, 69.1, 68.8, 66.3, 65.6, 62.2, 49. 7, 20.8, 20.7, 20.6, 12.8, 12.1. HRMS (ESI/Q-TOF) m/z: $[M+H]^+$ Calcd for $C_{57}H_{70}BF_2N_8O_{20}$ 1235.4771; Found 1235.4791; $[M+Na]^+$ Calcd for $C_{57}H_{69}$ $BF_2N_8NaO_{20}$ 1257.4591; Found 1257.4621.

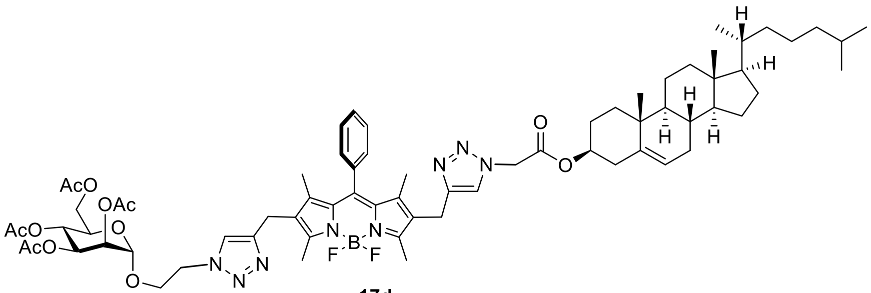

17d

Compound 17d. Following the general procedure D, compound **18** (35 mg, 0.04 mmol) and 1-ethylene-2-azido α-D-mannopyrannosyl glycoside (33.5 mg, 0.08 mmol) in CH_2Cl_2 (6 mL) were added to a solution of sodium ascorbate (24 mg, 0.12 mmol) and $CuSO_4$ (15 mg, 0.06 mmol) in H_2O (2 mL). The solution was placed in a glass seal tube (65 °C) for 12 h. The residue was purified by silica column chromatography (hexane/ethyl acetate: 4/6 to ethyl acetate) to afford BODIPY **17d** (40 mg, 78%). $[\alpha]_D^{21}$ = −205.5 (*c* 0.03, $CHCl_3$); ^1H NMR ($CDCl_3$, 400 MHz): δ 7.47-7.45 (m, 3H), 7.28-7.26 (m, 4H), 5.36-5.35 (m, 1H), 5.23 (t, *J* = 9.7 Hz, 1H), 5.17-5.12 (m, 2H), 5.04 (m, 2H), 4.78 (s, 1H), 4.70-4.62 (m, 1H), 4.51 (m, 2H), 4,20 (dd, *J* = 12.3, 5.1 Hz, 1H), 4.12-4.06 (m, 1H), 4.03-4.01 (m, 1H), 3.85-3.77 (m, 5H), 3.59 (m, 1H), 2.53 (s, 3H), 2.50 (s, 3H), 2.31-2.29 (m, 2H), 2.11 (s, 3H), 2.07 (s, 3H), 2.02 (s, 3H), 1.98 (m, 2H), 1.94 (s, 3H), 1.89-1.77 (m, 3H), 1.58-1.41 (m, 8H), 1.36-1.24 (m, 10H), 1.17-1.06 (m, 6H), 0.99 (s, 3H), 0.91 (d, *J* = 6.5 Hz, 3H), 0.85 (d, *J* = 6.6 Hz, 6H), 0.67 (s, 3H), $^{13}C\{^1H\}$ NMR (125 MHz, $CDCl_3$) δ 170.7, 170.1, 169.9, 169.7, 165.8, 154.7, 154.1, 141.5, 140.1, 139.8, 139.1, 135.4, 131.2, 131.0, 129.3, 129.1, 128.2, 128.0, 127.6, 123.4, 97.6, 77.5, 77.4, 77.2, 76.8, 76.5, 69.3, 69.2, 68.9, 66.4, 65.7, 62.3, 56.8, 56.2, 51.2, 50.1, 42.4, 39.8, 39.6, 38.0, 36.9, 36.6, 36.3, 35.9, 32.0, 31.9, 28.3, 28.1, 27.7, 24.4, 23.9, 22.9, 22.7, 21.1, 20.9, 20.8, 20.8, 20.7, 19.4, 18.8, 12.9, 12.2, 12.1, 12.0. HRMS (ESI/Q-TOF) m/z: $[M+H]^+$ Calcd for $C_{70}H_{94}BF_2N_8O_{12}$ 1287.7058; Found 1287.7065.

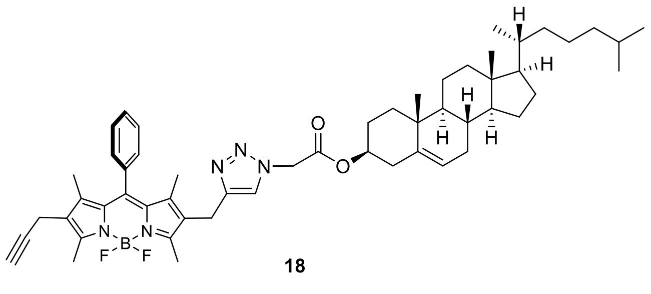

18

Compound 18. Following the general procedure D, BODIPY **10b** (40 mg, 0.1 mmol) and azido-cholesteryl derivative **15** (42.2 mg, 0.09 mmol) in CH_2Cl_2 (6 mL) were added to a solution of sodium ascorbate (30 mg, 0.15 mmol) and $CuSO_4$ (75 mg, 0.3 mmol) in H_2O (2 mL). The solution was placed in a glass seal tube (65 °C) for 12 h. The residue was purified by silica column chromatography (hexane/ethyl acetate: 7 :3) to afford derivative **18** (35 mg, 40%) along with **17b** (37 mg, 28%) . **Compound 18 :** $[\alpha]_D^{21}$ = −67.3 (*c* 0.04, $CHCl_3$); ^{1}H NMR ($CDCl_3$, 400 MHz): δ 7.49-7.47 (m, 3H), 7.28-7.25 (m, 2H), 7.21 (s, 1H), 5.38-5.37 (m, 1H), 5.05 (s, 2H), 4.72-4.64 (m, 1H), 3.81 (m, 2H), 3.20 (d, *J* = 2.8 Hz, 2H), 2.60 (s, 3H), 2.53 (s, 3H), 2.32-2.30 (m, 2H), 2.03-1.93 (m, 3H), 1.89-1.78 (m, 6H), 1.60-1.46 (m, 6H), 1.38-1.29 (m, 9H), 1.16-1.09 (m, 6H), 1.03-0.96 (m, 6H), 0.91 (d, *J* = 6.5 Hz, 3H), 0.86 (d, *J* = 6.6 Hz, 6H), 0.67 (s, 3H). ^{13}C {^{1}H} NMR ($CDCl_3$, 125 MHz) δ 165.7, 154.6, 154.1, 147.1, 141.7, 140.1, 139.6, 139.1, 135.3, 131.1, 131.0, 129.5, 129.3, 129.2, 128.2, 127.9, 127.7, 125.5, 123.5, 122.7, 81.2, 68.5, 56.8, 56.2, 51.2, 50.1, 42.4, 39.8, 39.6, 38.0, 36.9, 36.6, 36.1, 35.9, 32.0, 31.9, 28.4, 28.2, 27.7, 24.4, 24.0, 23.0, 22.7, 21.1, 20.8, 19.4, 18.8, 13.7, 13.0, 12.9, 12.2, 12.1, 12.0. HRMS (ESI/Q-TOF) m/z: $[M-F]^+$ Calcd for $C_{54}H_{70}BFN_5O_2$: 850.5607; Found 850.5603.

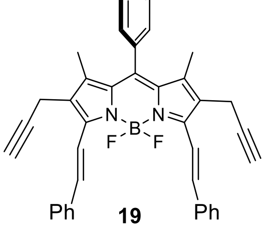

19

8-phenyl-1,7-distyryl-,3,5-dimethyl-2,6-di(prop-2-in-1-yl)-4,4-difluor-4-bora-3a,4a-diaza-s-indacene (19). To a solution of BODIPY **10b** (111 mg, 0.28 mmol) and benzaldehyde (141 μL, 1.39 mmol) in dry DMF (10 mL) were added piperidine (164 μL, 1.66 mmol) and acetic acid (95 μL, 1.66 mmol). The condensation reaction was performedunder MW irradiation for 1 h at 80 °C. The resulting crude mixture was then partitioned between a éter/toluene (9/1) solution and water, the aqueous layer was re-extracted twice. The organic phase was dried over $MgSO_4$,

concentrated. The residue was purified by silica column chromatography (hexane/ethyl acetate: 9:1) to afford derivative **19** (126 mg, 79%). Mp 279-281 °C; ^{1}H NMR (CDCl$_3$, 400 MHz): 7.79-7.75 (m, 2H), 7.68-7.66 (m, 4H), 7.53-7.32 (m, 13H), 3.44 (s, 4H), 2.09 (t, J = 2.7 Hz, 2H), 1.42 (s, 6H). ^{13}C {^{1}H} NMR (CDCl$_3$, 125 MHz) δ 151.3, 140.5, 140.4, 138.1, 137.1, 135.47, 132.9, 129.4, 129.3, 129.0, 128.9, 128.5, 127.7, 126.2, 119.2, 81.3, 69.3, 15.6, 12.0. HRMS (ESI/Q-TOF) m/z: [M+H]$^+$ Calcd for $C_{39}H_{31}BF_2N_2$ 576.2548; Found 576.2552.

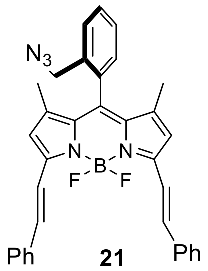

21

8-(2-azidomethylphenyl)-1,7-distyryl-,3,5-dimethyl-4,4-difluor-4-bora-3a,4a-diaza-s-indacene (21). To a solution of azido-BODIPY **20** (300 mg, 0.79 mmol) and benzaldehyde (404 μL, 3.9 mmol) in dry DMF (10 mL) were added piperidine (468 μL, 4.7 mmol) and acetic acid (267 μL, 4.7 mmol). The condensation reaction was performed under MW irradiation for 1 h at 80 °C. The resulting crude mixture was then partitioned between a ether/toluene (9/1) solution and water, the aqueous layer was re-extracted twice. The organic phase was dried over MgSO$_4$, concentrated. The residue was purified by silica column chromatography (hexane/ethyl acetate: 9:1) to afford derivative **21** (400 mg, 91%). Mp 212-215 °C; ^{1}H NMR (CDCl$_3$, 400 MHz): δ 7.79-7.75 (m, 2H), 7.65-7.64 (m, 4H), 7.60-7.25 (m, 12H), 6.66 (s, 2H), 4.37 (s, 2H), 1.39 (s, 6H). ^{13}C {^{1}H} NMR (CDCl$_3$, 125 MHz) δ 153.1, 141.9, 136.8, 136.6, 136.1, 134.3, 133.1, 132.9, 130.0, 129.2, 129.2, 129.1, 128.9, 127.7, 119.2, 118.2, 52.2, 14.3. HRMS (ESI/Q-TOF) m/z: [M+H]$^+$ Calcd for $C_{34}H_{28}BF_2N_5$ 556.2485; Found 556.2474.

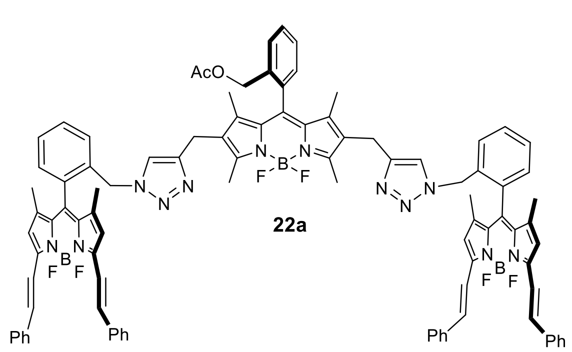

22a

Compound 22a. Following the general procedure D, compound **10a** (35 mg, 0.075 mmol) and azido-BODIPY **21** (108 mg, 0.19 mmol) in CH_2Cl_2 (6 mL) were added to a solution of sodium ascorbate (45 mg, 0.22 mmol) and $CuSO_4$ (28 mg, 0.11 mmol) in H_2O (2 mL). The solution was placed in a glass seal tube (65 °C) for 4 h. The residue was purified by silica column chromatography (hexane/ethyl acetate: 9/1 to 2 /8) to afford BODIPY triad **22a** (105 mg, 89%). Mp 230-235 °C; ^{1}H NMR ($CDCl_3$, 400 MHz): δ 7.72-7.67 (m, 4H), 7.59-7.23 (m, 36H), 7.05-7.03 (m, 2H), 6.76 (m, 2H), 6.65-6.64 (m, 4H), 5.34-5.25 (m, 4H), 4.82 (m, 2H), 3.49 (m, 4H), 2.46 (s, 6H), 1.70 (s, 3H), 1.26-1.24 (m, 12H), 1.04 (s, 6H). ^{13}C {1H} NMR ($CDCl_3$, 125 MHz) δ 170.3, 154.4, 153.3, 146.3, 141.9, 139.7, 138.6, 137.2, 137.2, 136.5, 136.4, 135.4, 133.9, 133.8, 13355, 133.1, 132.8, 130.4, 130.2, 129.5, 129.2, 129.1, 129.0, 128.9, 128.8, 128.5, 128.3, 127.9, 127.6, 122.1, 119.1, 119.0, 118.3, 118.3, 62.8, 51.1, 20.6, 20.5, 14.2, 12.8, 11.5. ^{19}F NMR ($CDCl_3$, 376 MHz) δ −137.07 (dq, J = 103.0, 28.3 Hz, 2F), −137.88 (dq, J = 103.0, 28.3 Hz, 2F), −143.52- −144.36 (m, 2F). HRMS (ESI/Q-TOF) m/z: $[M+H]^+$ Calcd for $C_{96}H_{83}B_3F_6N_{12}O_2$ 1583.7055; Found 1583.7082.

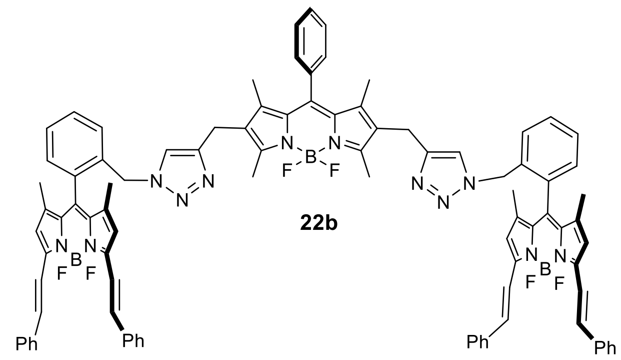

Compound 22b. Following the general procedure D, compound **10b** (40 mg, 0.1 mmol) and azido-BODIPY **21** (144 mg, 0.26 mmol) in CH_2Cl_2 (6 mL) were added to a solution of sodium ascorbate (60 mg, 0.3 mmol) and $CuSO_4$ (37 mg, 0.15 mmol) in H_2O (2 mL). The solution was placed in a glass seal tube (65 °C) for 24 h. The residue was purified by silica column chromatography (hexane/ethyl acetate: 9/1 to 1/1) to afford BODIPY triad **22b** (90mg, 60%). Mp 208-211 °C; ^{1}H NMR ($CDCl_3$, 400 MHz): δ 7.74-7.70 (m, 2H), 7.59-7.57 (m, 4H), 7.52-7.21 (m, 25H), 7.12-7.09 (m, 2H), 6.68-6.65 (m, 4H), 5.30 (s, 4H), 3.54 (s, 4H), 2.46 (s, 6H), 1.27 (s, 12H), 1.06 (s, 6H). ^{13}C {^{1}H} NMR ($CDCl_3$, 125 MHz) δ 154.1, 153.5, 146.7, 141.9, 141.4, 140.1, 137.3, 136.6, 135.4, 135.1, 134.0, 133.1, 132.9, 131.0, 130.5, 130., 129.6, 129.3, 129.2, 129.0, 128.9, 128.1, 127.7, 121.9, 119.2, 118.4, 51.2, 20.7, 14.3, 12.9, 12.1. ^{19}F NMR ($CDCl_3$, 376 MHz) δ −138.30 (dq, J = 101.4, 31.5 Hz, 2F), −139.31 (dq, J = 101.4, 31.5 Hz, 2F), −145.12- −145.36 (m, 2F). HRMS (ESI/Q-TOF) m/z: $[M+H]^+$ Calcd for $C_{93}H_{79}B_3F_6N_{12}$ 1511.6843; Found 1511.6893.

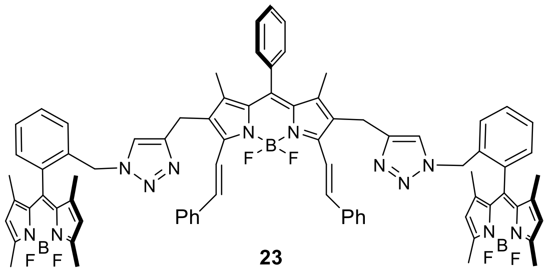

Compound 23. Following the general procedure D, compound **19** (41 mg, 0.071 mmol) and azido-BODIPY **20** (70 mg, 0.18 mmol) in CH_2Cl_2 (8 mL) were added to a solution of sodium ascorbate (42 mg, 0.21 mmol) and $CuSO_4$ (26 mg, 0.11 mmol) in H_2O (2 mL). The solution was placed in a glass seal tube (65 °C) for 12 h. The residue was purified by silica column chromatography (hexane/ethyl acetate: 6/4) to afford BODIPY triad **23** (54 mg, 56%). Mp 210-213 °C; ^{1}H NMR ($CDCl_3$, 400 MHz): δ 7.78-7.72 (m, 2H), 7.54-7.29 (m, 21H), 7.12-7.06 (m, 2H), 6.74 (s, 2H), 5.95 (s, 4H), 5.34 (s, 4H), 4.00 (s, 4H), 2.48 (s, 12H), 1.33 (s, 6H), 1.16 (s, 12H). ^{13}C {^{1}H} NMR ($CDCl_3$, 125 MHz) δ 156.7, 151.3, 142.9, 140.8, 140.0, 138.1, 137.8, 136.9, 135.3, 133.8, 133.0, 132.7, 131.0, 130.2, 130.2, 129.6, 129.6, 129.4, 129.1, 128.9, 128.6, 128.2, 127.6, 121.9, 119.1, 51.1, 22.6, 14.7, 14.0, 12.2. ^{19}F NMR ($CDCl_3$, 376 MHz) δ −139.04 (q, *J* = 30.1 Hz, 2F), −146.16 (dq, *J* = 108.1, 30.4 Hz, 2F), −147.69 (dq, *J* = 108.1, 30.4 Hz, 2F). HRMS (ESI/Q-TOF) m/z: $[M+H]^+$ Calcd for $C_{79}H_{72}B_3F_6N_{12}$ 1335.6286; Found 1335.6269.

4. Photophysical properties

Spectroscopic signatures were recorded using diluted dye solutions (*ca.* 2 × 10^{-6} M) prepared from a concentrated stock solution in acetone (*ca.* 10^{-3} M), after solvent evaporation under reduced pressure, and subsequent dilution with the desired solvent of spectroscopic grade. UV-vis absorption and fluorescence spectra were recorded on a Varian (model CARY 4E) spectrophotometer and an Edinburgh Instrument spectrofluorometer (model FLSP 920), respectively. Fluorescence quantum yields (ϕ*)* were determined from corrected spectra (detector sensibility to the wavelength) by the optically dilute relative method. PM567 (ϕ = 0.84 in ethanol) for simple yellow-emitting BODIPYs and cresyl violet (ϕ = 0.54 in methanol) for the red-emitting cassettes were used as references. The aforementioned spectrofluorometer is also equipped with a wavelength-tunable pulsed Fianium laser. Thus, the Time Correlated Single-Photon Counting (TCSPC) technique was used to record the fluorescence decay curves. Fluorescence emission was monitored at the maximum emission wavelength after excitation by

the said Fianium at the maximum absorption wavelengths. The fluorescence lifetime (τ) was obtained from the slope of the exponential fit of the decay curve, after the deconvolution of the instrumental response signal (recorded by means of a ludox scattering suspension) by means of an iterative method. The goodness of the exponential fit was controlled by statistical parameters (chi-square and the analysis of the residuals). The radiative (k_{fl}) and non-radiative (k_{nr}) rate constants were calculated from the fluorescence quantum yield and lifetime; $k_{fl} = \phi/\tau$ and $k_{nr} = (1-\phi)/\tau$.

5. Lasing properties

Laser efficiency was evaluated from concentrated solutions (milimolar) of dyes in ethyl acetate contained in 1-cm optical-path rectangular quartz cells carefully sealed to avoid solvent evaporation during experiments. The liquid solutions were transversely pumped with 5 mJ, 8 ns FWHM pulses from the second (532 nm) and third (355 nm) harmonic of a Q-switched Nd:YAG laser (Lotis TII 2134) at a repetition rate of 1 Hz. The exciting pulses were line-focused onto the cell using a combination of positive and negative cylindrical lenses (f = 15 cm and f = -15 cm, respectively) perpendicularly arranged. The plane parallel oscillation cavity (2 cm length) consisted of a 90% reflectivity aluminium mirror acting as back reflector, and the lateral face of the cell acting as output coupler (4% reflectivity). The pump and output energies were detected by a GenTec powermeter. The photostability of the dyes in ethyl acetate solution was evaluated by using a pumping energy and geometry equal to the laser experimental set-up. We used spectroscopic quartz cuvettes with 0.1 cm optical to allow for the minimum solution volume (40 µL) to be excited. The lateral faces were grounded, whereupon no laser oscillation was obtained. Information about photostabilitiy was obtained by monitoring the decrease in laser-induced fluorescence (LIF) intensity after 130 000 pump pulses and 10 Hz repetition rate to speed up the experimental running. Laser emission emissions was monitored perpendicular to the exciting beam, collected by an optical fiber, and imaged onto a spectrometer (Acton Research corporation) and detected with a charge-coupled device (CCD) (SpectruMM:GS128B). Laser-induced emission was monitored in front-face configuration and recorded by feeding the signal to the boxcar (Stanford Research, model 250) to be integrated before being digitized and processed by a computer. The estimated error in the energy and photostability measurements was 10%.

6. Computational chemistry

Ground state geometries were optimized with the b3lyp hybrid functional, within the Density Functional Theory (DFT), using the double valence basis set with a polarization function (6-31G*). The geometries were considered as energy minimum when the corresponding frequency analysis did not give any negative value. The absorption spectra was simulated as a Franck-Condon vertical transition from the optimized ground state geometries using the time dependent method with the aforementioned functional and basis set (td b3lyp/6-31g*). All the theoretical calculations were carried out using the GAUSSIAN 16 program suite, implemented in the computational cluster provided by the SGIker resources of UPV-EHU.

7. Table S1. Photophysical[a)] and lasing[b)] properties of representative propargylated BODIPYs at positions 2 and/or 6.

The corresponding data of the commercially available BODIPYs 2 (PM567) in ethyl acetate are also added for comparison (in italics).

	λ_{ab} (nm)	$\varepsilon_{max}\cdot10^{-4}$ (M^{-1} cm^{-1})	λ_{fl} (nm)	ϕ	τ (ns)	k_{fl} (10^8 s^{-1})	k_{nr} (10^8 s^{-1})	λ_{la} (nm)	Eff(%)
**3**									
EtOAc	509.0	8.4	527.0	0.97	5.79	1.67	0.05	546	59
ACN	506.0	9.2	525.5	0.97	5.99	1.62	0.05		
**10b**									
EtOAc	514.0	7.4	526.0	0.77	4.78	1.61	0.48	546	54
ACN	512.0	6.2	524.5	0.69	4.73	1.46	0.65		
**11b**									
EtOAc	506.5	6.9	518.0	0.68	4.19	1.62	0.76	541	46
ACN	504.5	6.5	516.5	0.60	4.12	1.45	0.97		
**12**									
EtOAc	508.0	6.4	524.5	1.00	5.91	1.69	0.00	546	61
ACN	506.0	6.0	521.5	1.00	6.22	1.61	0.00		
**13**									
EtOAc	513.5	9.3	525.0	0.92	5.73	1.61	0.14	543	56
ACN	512.0	8.2	522.5	0.90	5.80	1.55	0.17		
2	*517.0*	*7.6*	*533.0*	*0.84*	*5.78*	*1.45*	*0.28*	*568*	*48*

a) Dye concentration: 2 μM. Absorption (λ_{ab}) and fluorescence (λ_{fl}) wavelength, molar absorption (ε_{max}), fluorescence quantum yield (ϕ) and lifetime (τ), radiative (k_{fl}) and non-radiative (k_{nr}) rate constants.

b) Dye concentrations in ethyl acetate ranging from 1×10^{-3} M to 7×10^{-3} M to match the optical density at the irradiation wavelength (532 nm). Laser wavelength (λ_{la}) and efficiency (%Eff).

EtOAc: ethyl acetate; ACN: acetonitrile

8. Table S2. Photophysical [a] and lasing[b] properties of BODIPYs after click conjugation with biomolecules.

	λ_{ab} (nm)	$\varepsilon_{max}\cdot10^{-4}$ (M^{-1} cm^{-1})	λ_{fl} (nm)	ϕ	τ (ns)	k_{fl} (10^8 s^{-1})	k_{nr} (10^8 s^{-1})	λ_{la} (nm)	Eff(%)
**17a**									
EtOAc	519.0	6.5	532.0	0.79	4.74	1.66	0.44	549	52
ACN	517.0	4.6	530.0	0.67	4.64	1.44	0.71		
**17b**									
EtOAc	518.5	5.8	532.0	0.75	4.88	1.54	0.51	550	47
ACN	517.0	5.1	530.5	0.74	4.90	1.51	0.53		
**17c**									
EtOAc	519.0	9.3	532.5	0.81	5.03	1.61	0.38	549	55
ACN	517.5	8.8	531.0	0.76	5.04	1.51	0.47		
**17d**									
EtOAc	518.5	7.4	532.0	0.78	4.89	1.59	0.45	547	51
ACN	517.0	7.0	530.5	0.78	4.98	1.57	0.44		

a) Dye concentration: 2 μM. Absorption (λ_{ab}) and fluorescence (λ_{fl}) wavelength, molar absorption (ε_{max}), fluorescence quantum yield (ϕ) and lifetime (τ), radiative (k_{fl}) and non-radiative (k_{nr}) rate constants.

b) Dye concentrations in ethyl acetate ranging from 6×10^{-4} M to 9×10^{-4} M to match the optical density at the irradiation wavelength (532 nm). Laser wavelength (λ_{la}) and efficiency (%Eff).

EtOAc: ethyl acetate; ACN: acetonitrile

9. Table S3. Photophysical[a)] and lasing[b)] properties of the all-BODIPYs based cassettes.

The corresponding data of the isolated red-emitting fragment 19 are also added for comparison.

	λ_{ab} (nm)	$\varepsilon_{max}\cdot10^{-4}$ (M^{-1} cm^{-1})	λ_{fl} (nm)	ϕ*	τ* (ns)	λ_{la}* (nm)	Eff(%)*
**22a**							
EtOAc	624.5<br>523.5<br>346.0	17.7<br>8.7<br>14.6	640.5	0.61	1.86(9%)-4.67(81%)		
ACN	623.0<br>522.5<br>345.5	14.9<br>8.4<br>12.5	639.5	0.19	1.16(70%)-2.86(30%)		
**22b**							
EtOAc	625.0<br>521.0<br>346.0	15.3<br>7.8<br>13.0	637.0	0.46	2.17(17%)-4.37(83%)	708	12
ACN	623.0<br>519.0<br>345.0	12.1<br>6.9<br>10.4	635.0	0.11	0.47(21%)-1.14(79%)		
**23**							
EtOAc	628.0<br>502.0<br>344.0	6.5<br>13.2<br>6.6	644.0	0.64	4.81	712	25
ACN	625.0<br>502.0<br>345.0	8.0<br>12.7<br>6.6	644.5	0.38	2.67		
**19**							
EtOAc	617.0<br>344.0	7.0<br>6.2	636.5	0.73	4.86	701	31
ACN	617.0<br>343.0	6.8<br>5.8	636.5	0.81	5.10		

a) Dye concentration: 2 μM. Absorption (λ_{ab}) and fluorescence (λ_{fl}) wavelength, molar absorption (ε_{max}), fluorescence quantum yield (ϕ) and lifetime (τ).

b) Dye concentrations in ethyl acetate ranging from 1×10^{-4} M to 1×10^{-3} M to match the optical density at the irradiation wavelengths (532nm and 355 nm). Laser wavelength (λ_{la}) and efficiency (%Eff).

EtOAc: ethyl acetate; ACN: acetonitrile

*these fluorescence and laser parameters remain the same regardless of the excited fragment

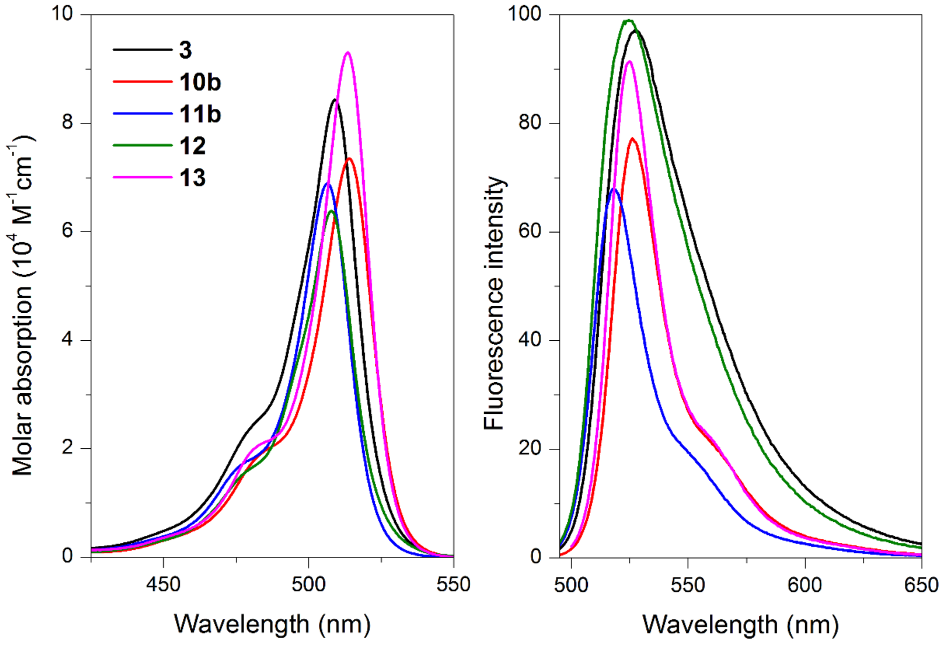

10. Figure S1. Absorption and fluorescence spectra of the propargylated BODIPYs in diluted solutions (2 μM) of ethyl acetate.

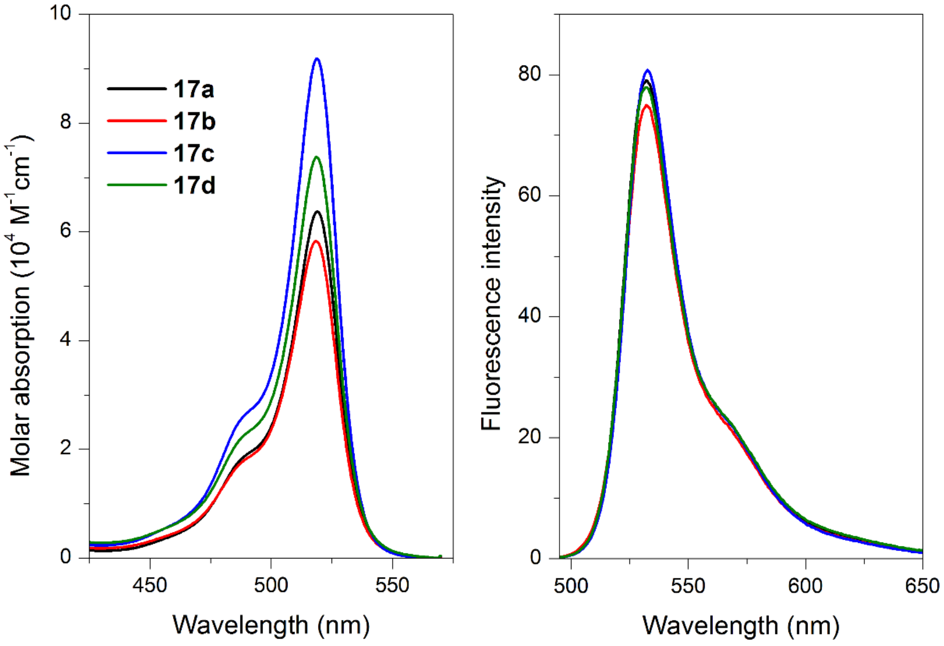

11. Figure S2. Absorption and fluorescence spectra of clickable BODIPYs at 2 and 6 positions in diluted solutions (2 μM) of ethyl acetate.

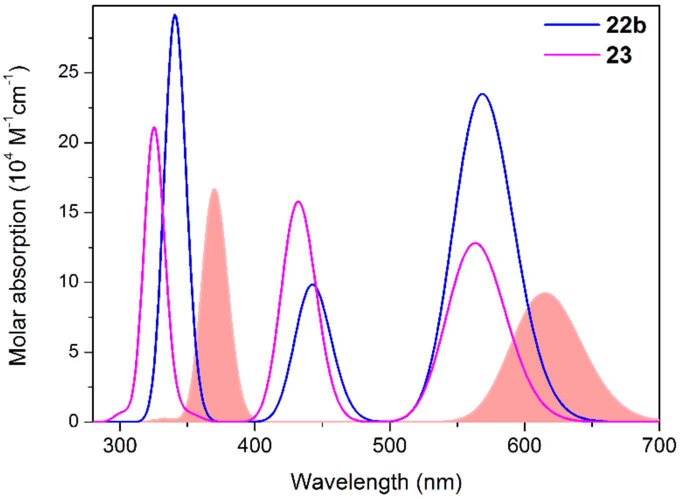

12. Figure S3. Theoretically predicted absorption spectra (td b3lyp/6-31g*) of triads 22b and 23.

The corresponding absorption spectrum of the isolated red-emitting fragment **19** is added for comparison.

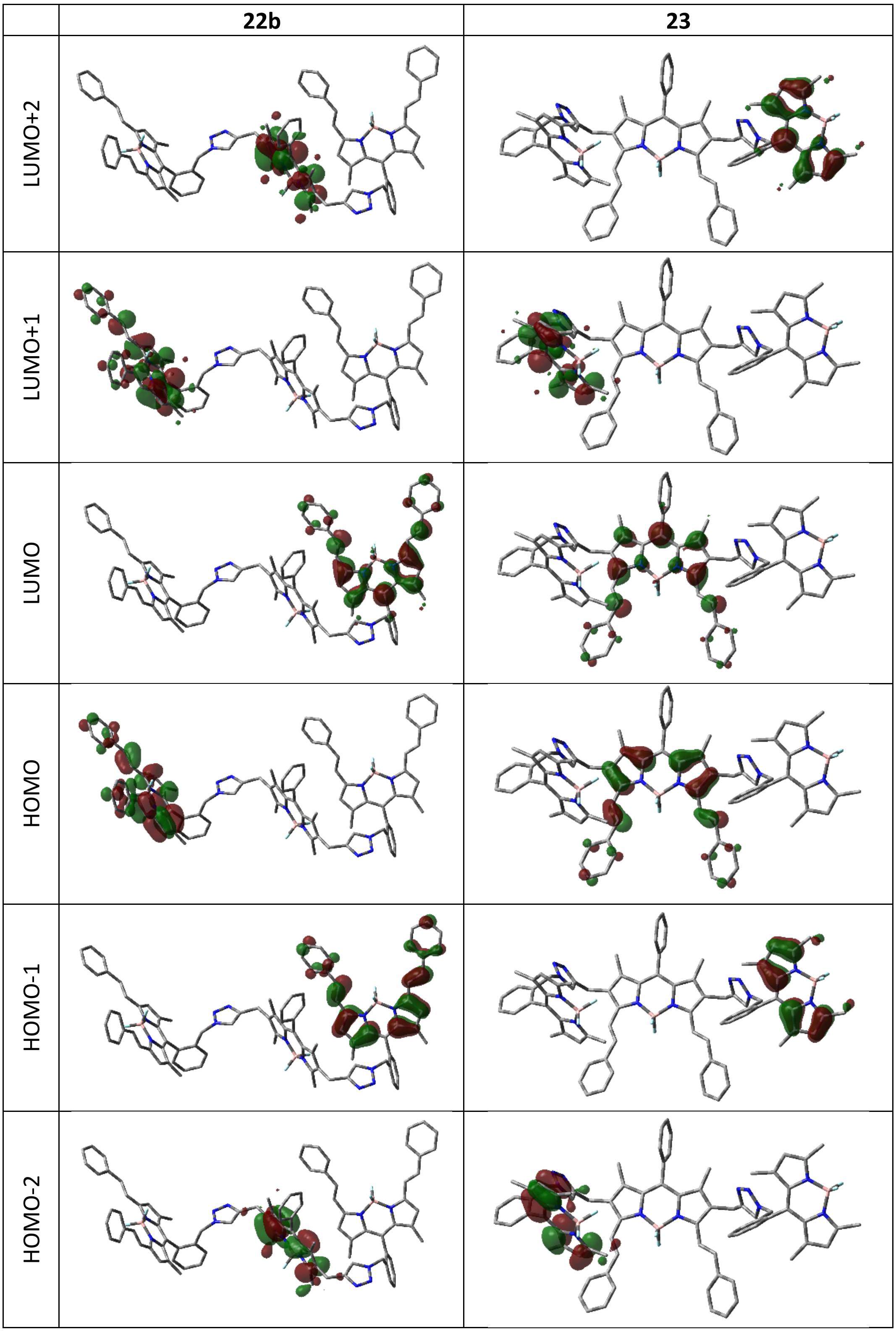

13. Figure S4. Theoretically calculated (b3lyp/6-31g*) molecular orbitals involved in the main visible electronic transitions from ground state optimized geometries of the all-BODIPY based cassettes.

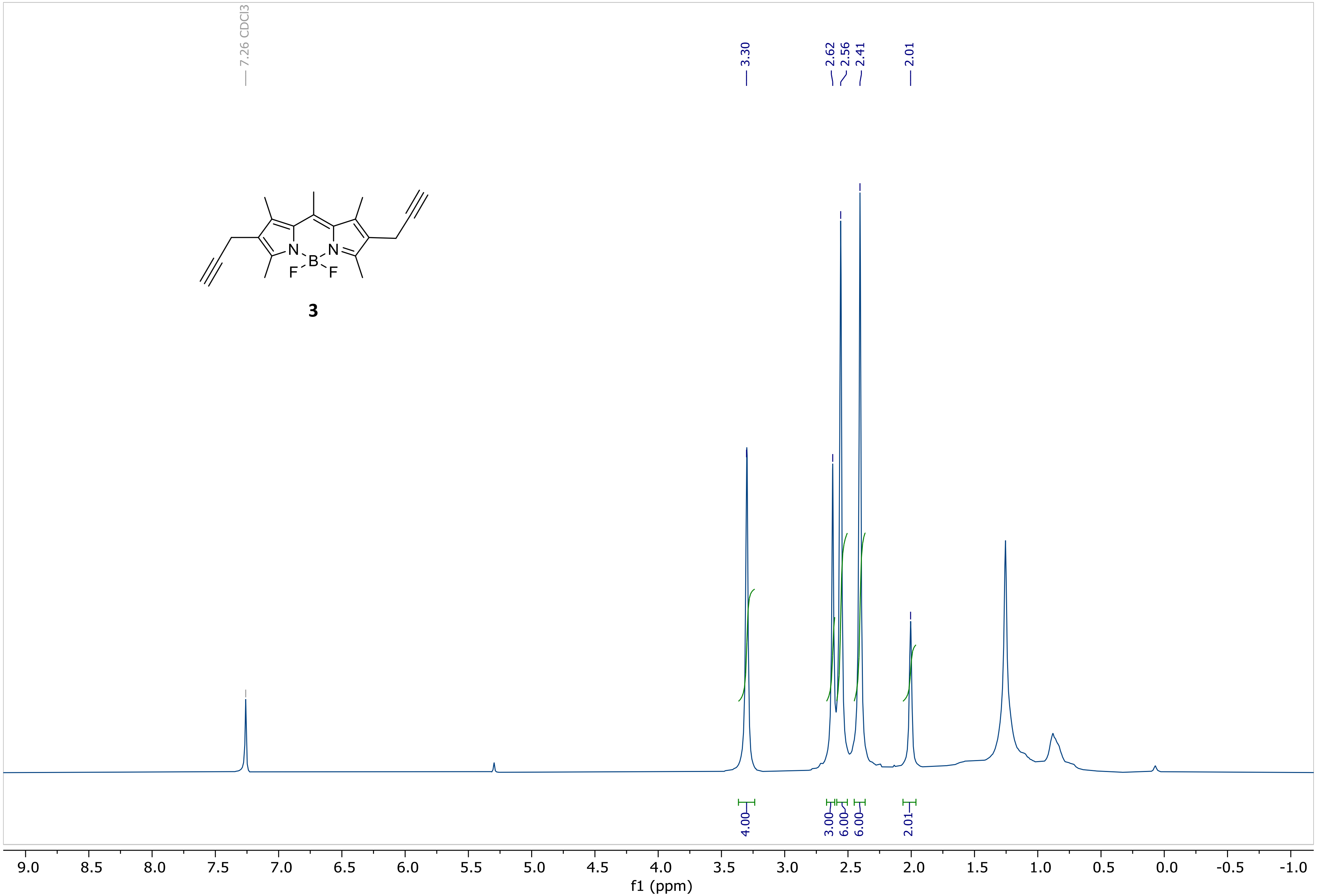

1H NMR (400MHz, CDCl3)

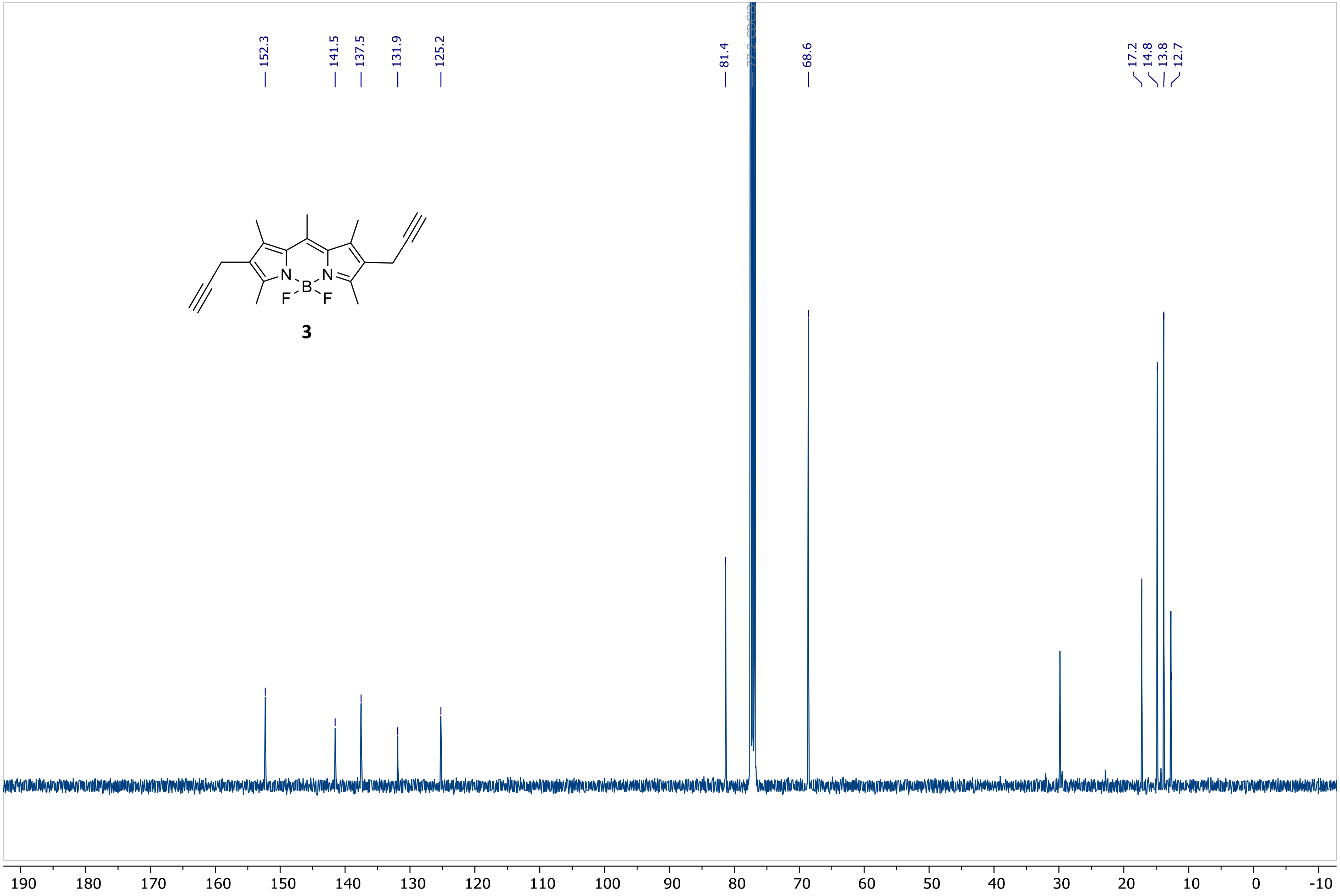

13C {1H} NMR (125 MHz, CDCl3)

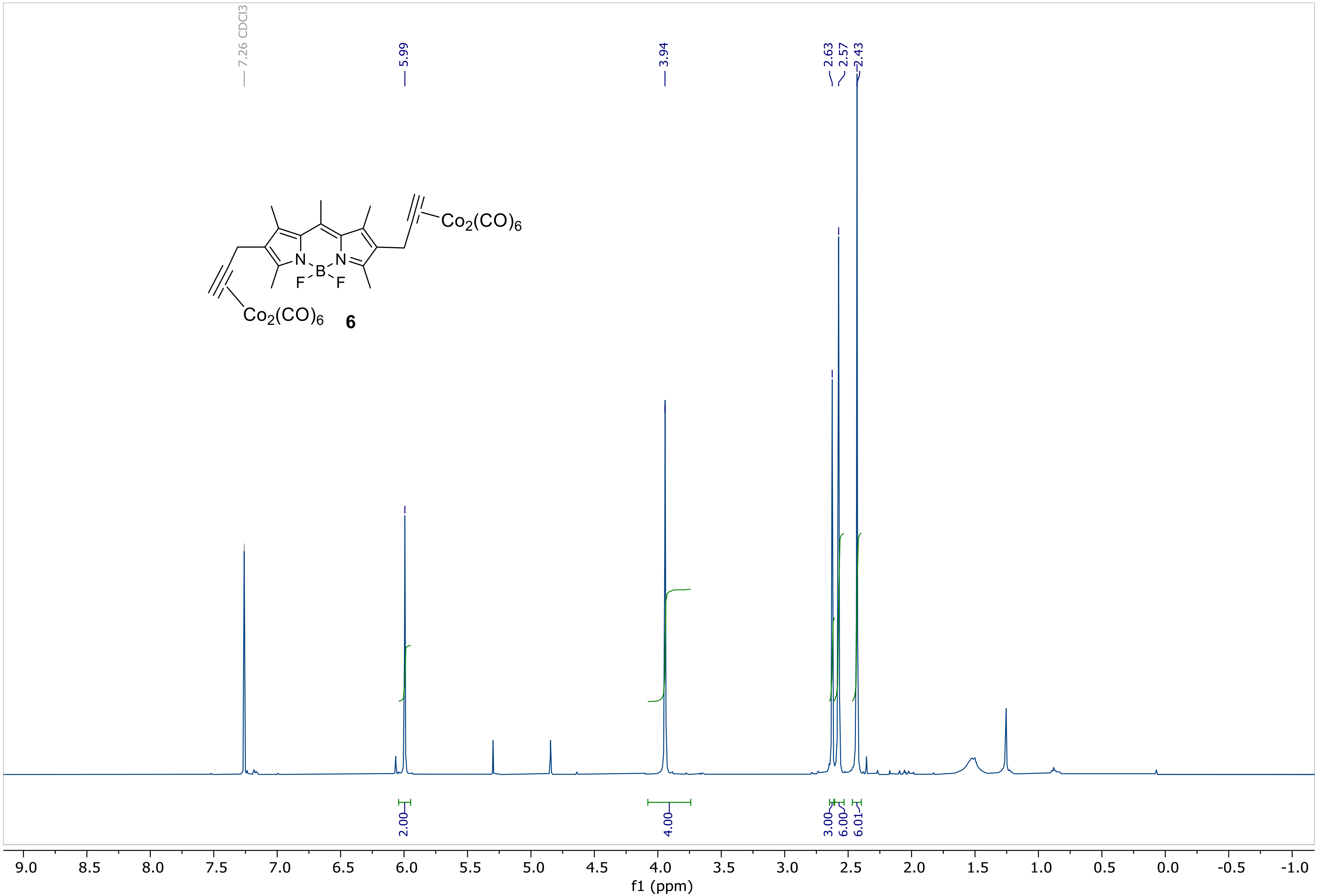

1H-NMR (400 MHz, CDCl3)

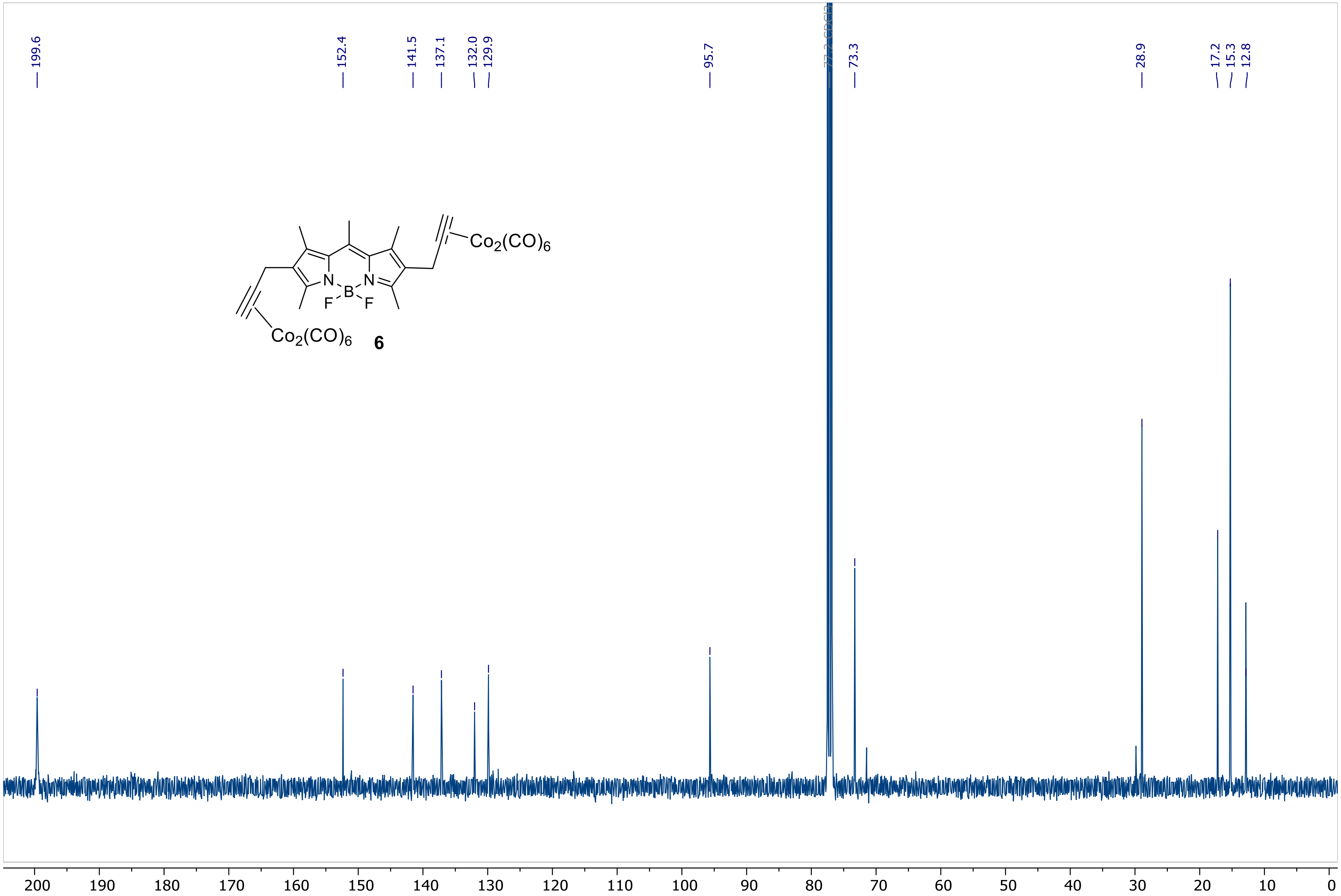

13C {1H} NMR (125 MHz, CDCl3)

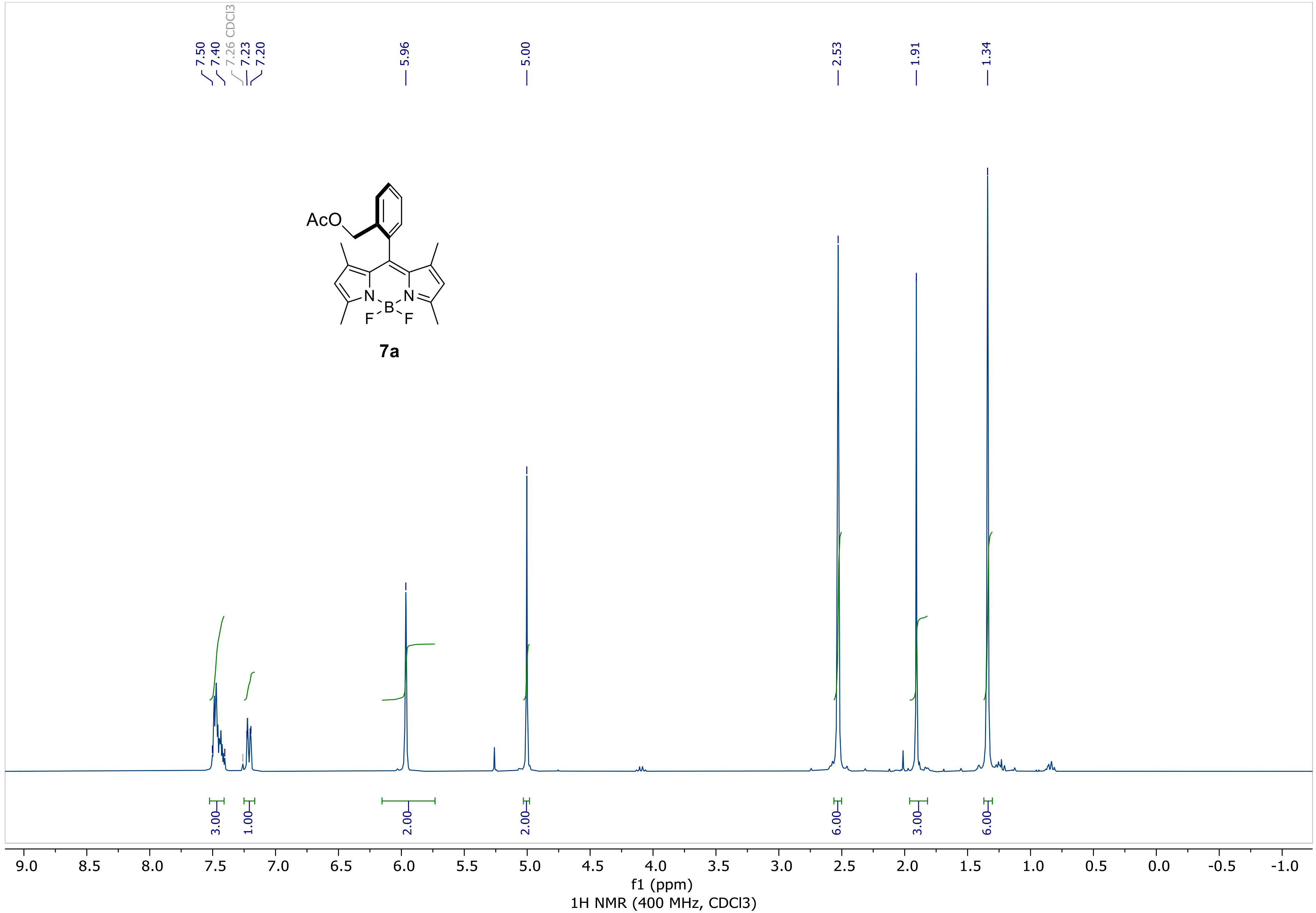
7.50
7.40
7.26 CDCl3
7.23
7.20
5.96
5.00
2.53
1.91
1.34
AcO
N
B
N
F
F
7a
3.00
1.00
2.00
2.00
6.00
3.00
6.00
9.0 8.5 8.0 7.5 7.0 6.5 6.0 5.5 5.0 4.5 4.0 3.5 3.0 2.5 2.0 1.5 1.0 0.5 0.0 -0.5 -1.0
f1 (ppm)
1H NMR (400 MHz, CDCl3)

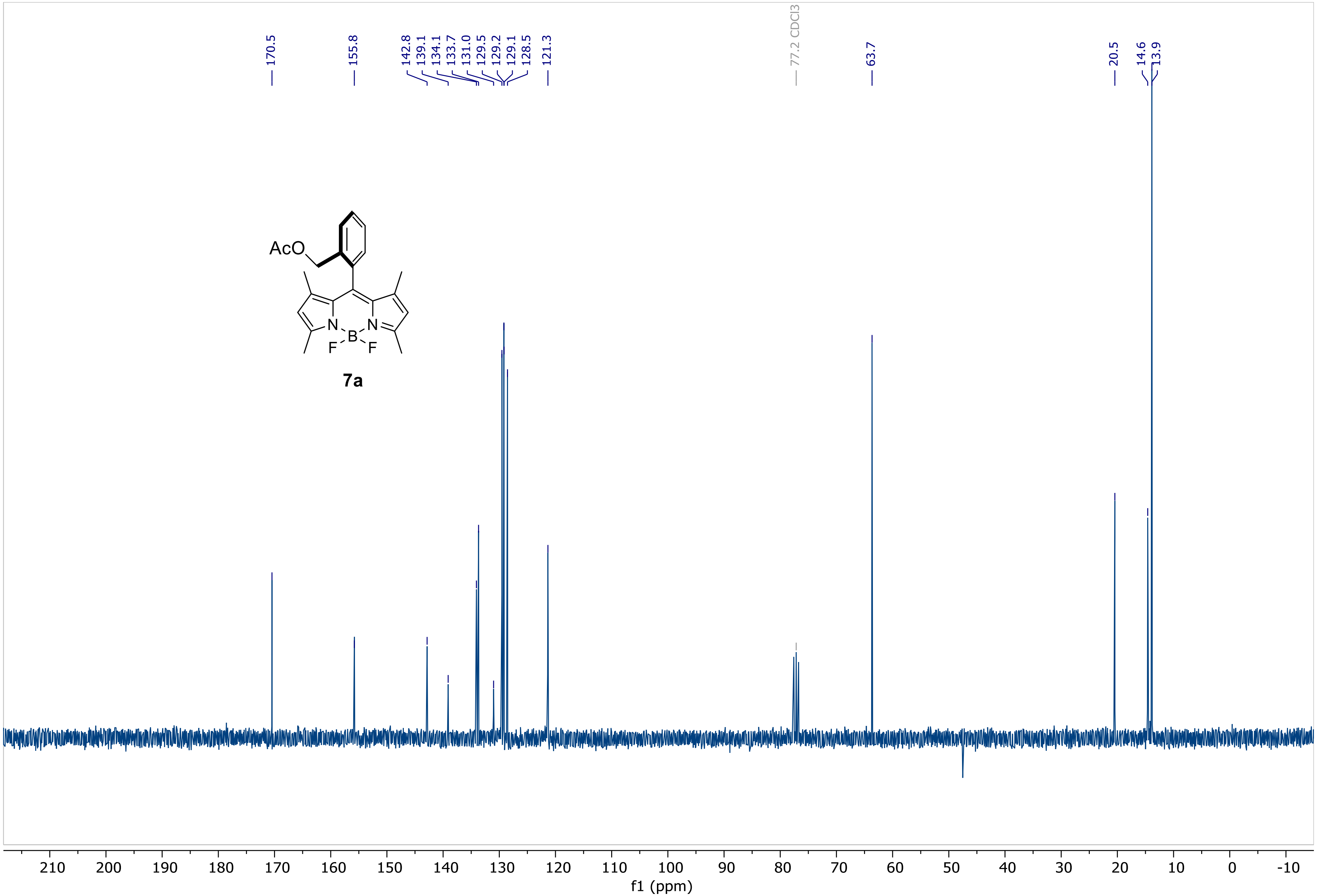

13C {1H} NMR (125 MHz, CDCl3)

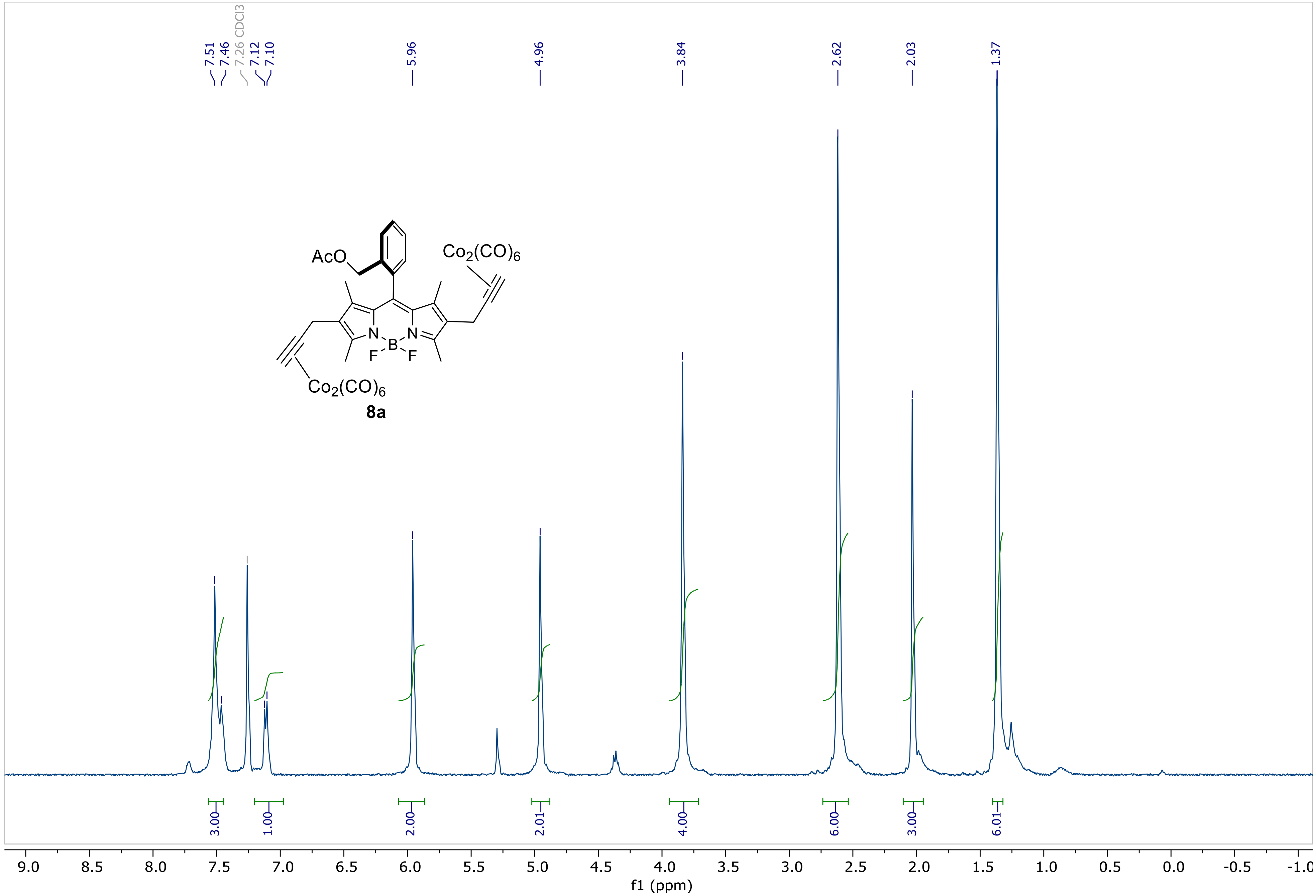

1H NMR (400 MHz, $CDCl_3$)

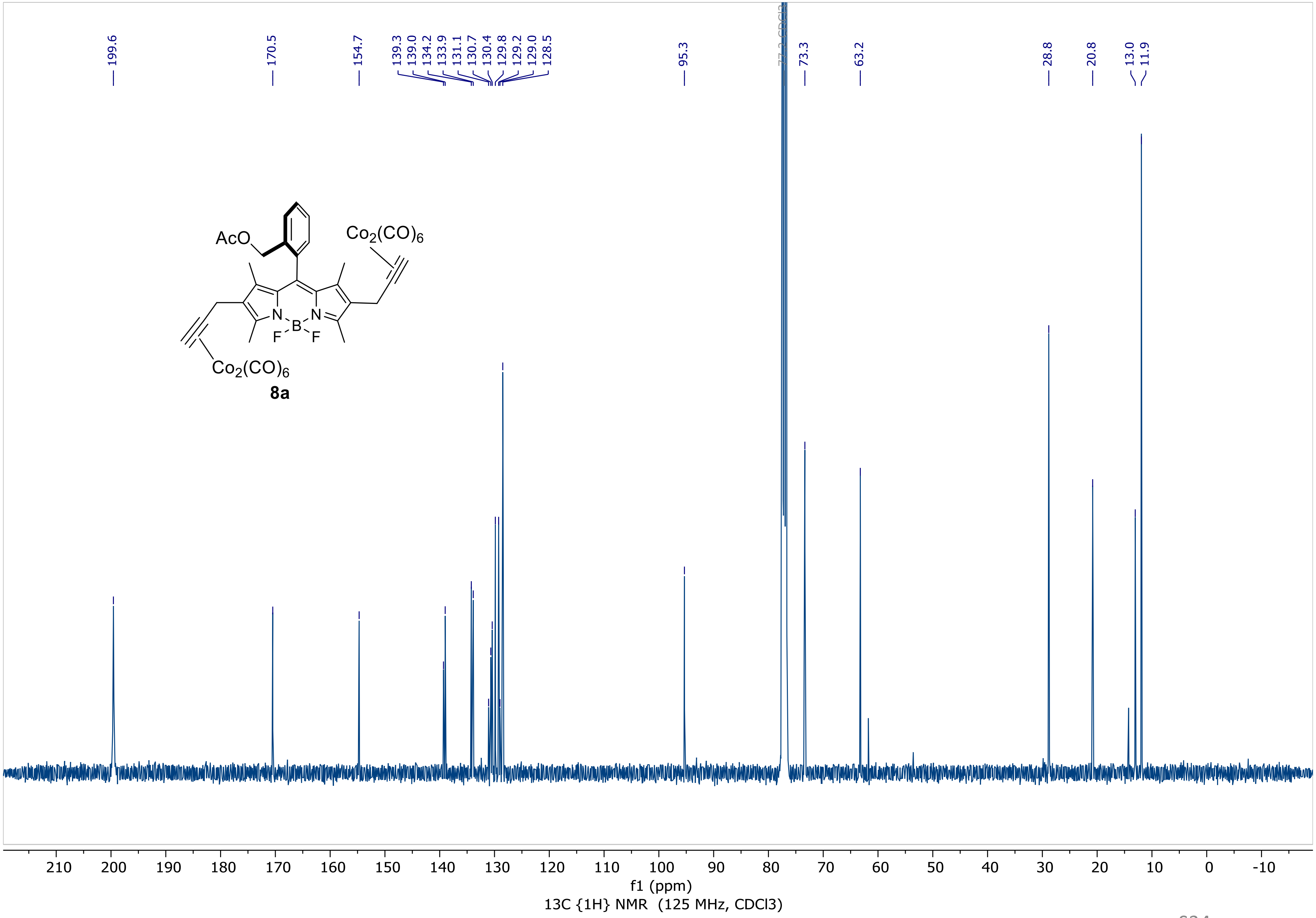
199.6
170.5
154.7
139.3
139.0
134.2
133.9
131.1
130.7
130.4
129.8
129.2
129.0
128.5
95.3
77.2 CDCl3
73.3
63.2
28.8
20.8
13.0
11.9
AcO
Co2(CO)6
N
B
F
Co2(CO)6
8a
210 200 190 180 170 160 150 140 130 120 110 100 90 80 70 60 50 40 30 20 10 0 -10
f1 (ppm)
13C {1H} NMR (125 MHz, CDCl3)

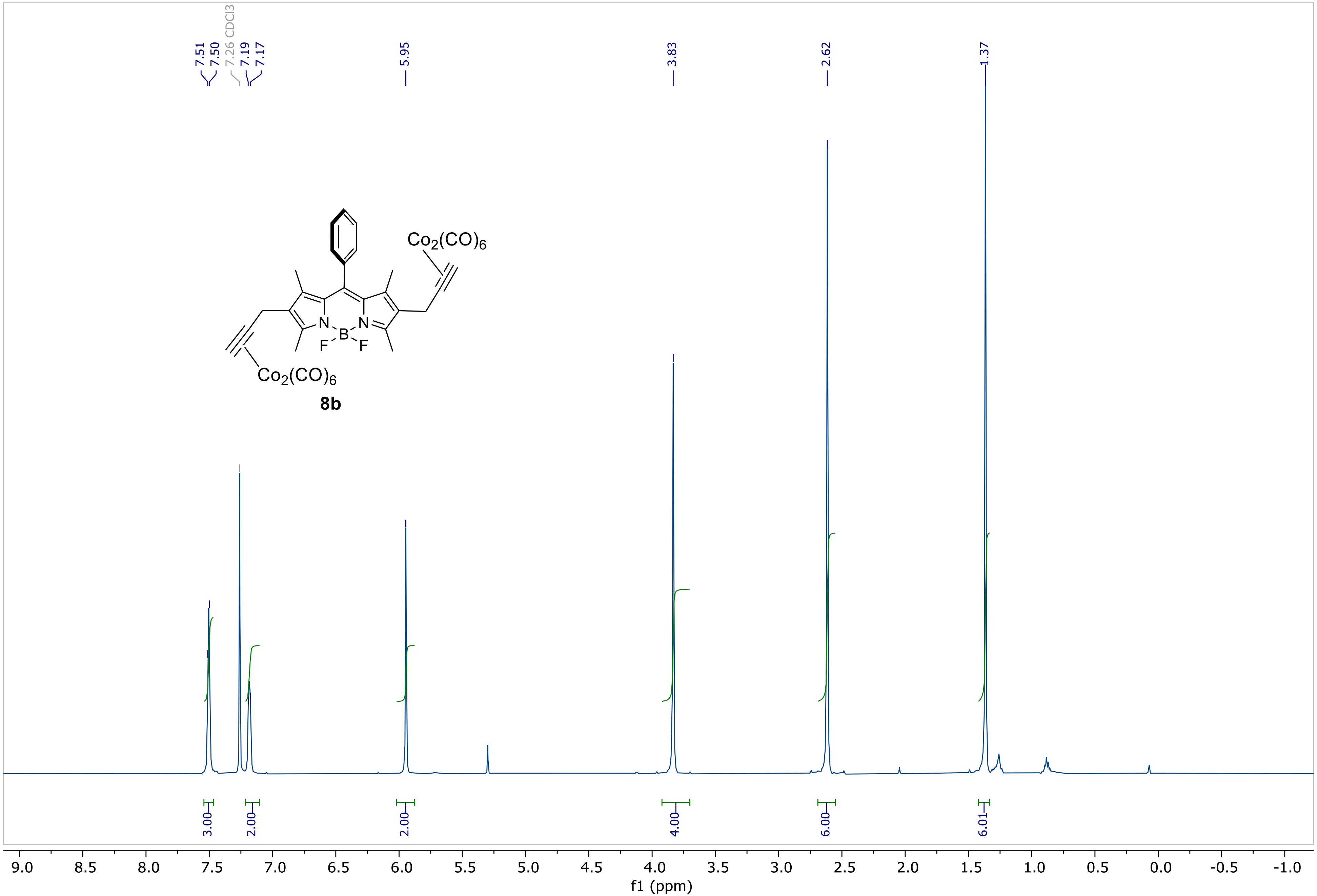

1H NMR (400 MHz, CDCl3)

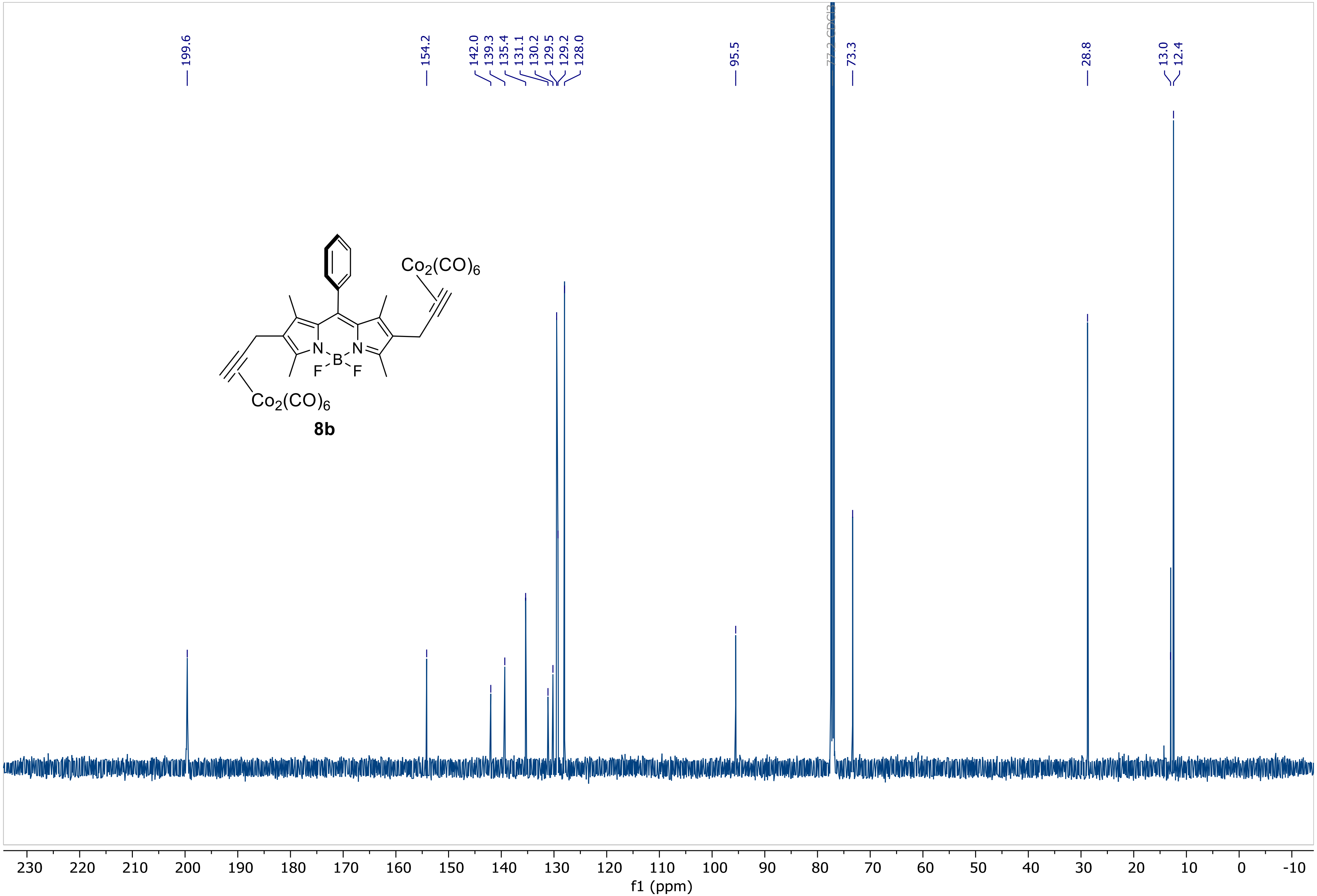

13C {1H} NMR (125 MHz, CDCl3)

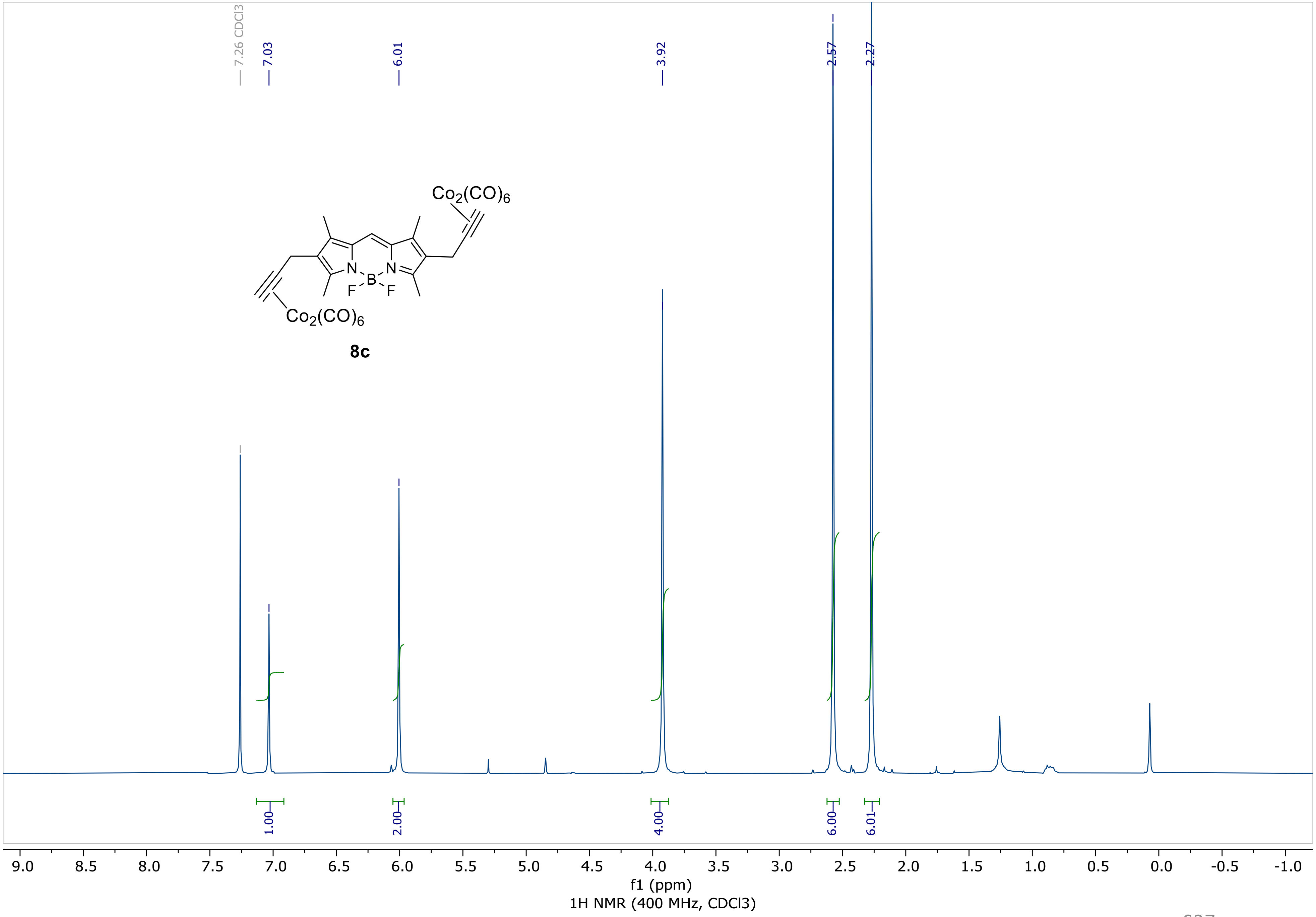
7.26 CDCl3
7.03
6.01
3.92
2.57
2.27
Co2(CO)6
N
B
N
F
F
Co2(CO)6
8c
1.00
2.00
4.00
6.00
6.01
9.0
8.5
8.0
7.5
7.0
6.5
6.0
5.5
5.0
4.5
4.0
3.5
3.0
2.5
2.0
1.5
1.0
0.5
0.0
-0.5
-1.0
f1 (ppm)
1H NMR (400 MHz, CDCl3)

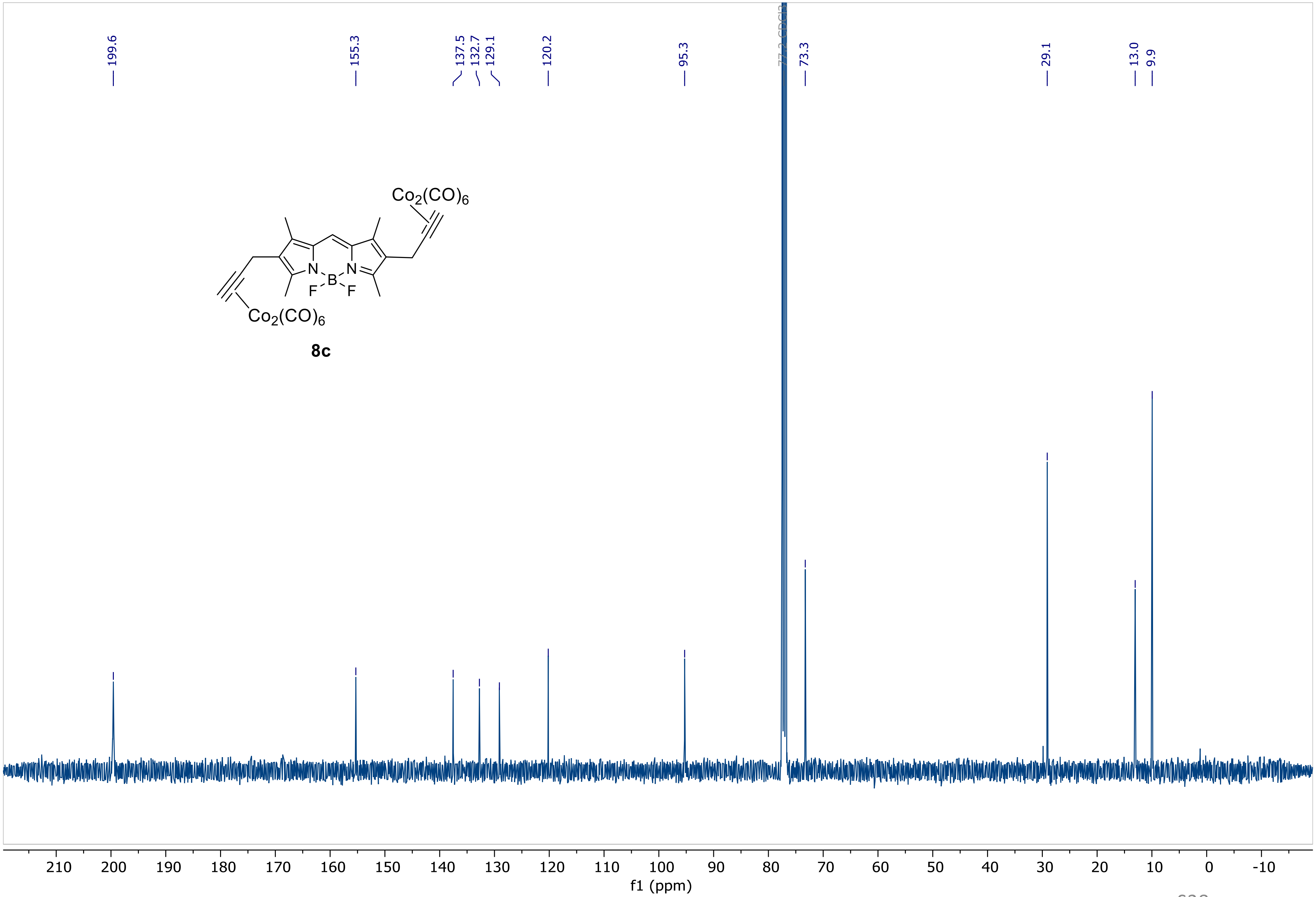

13C {1H} NMR (125 MHz, CDCl3)

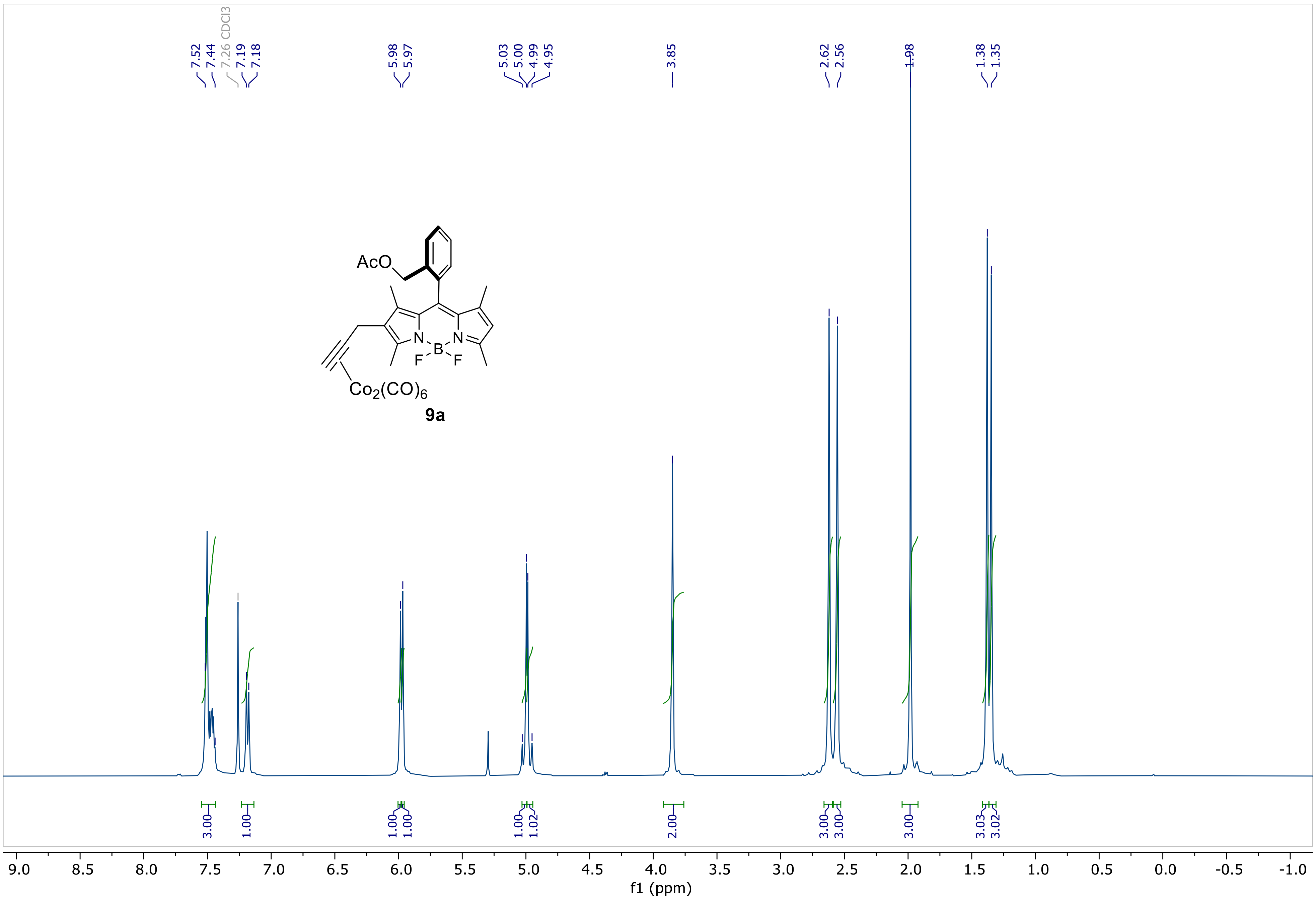

1H NMR (400 MHz, CDCl3)

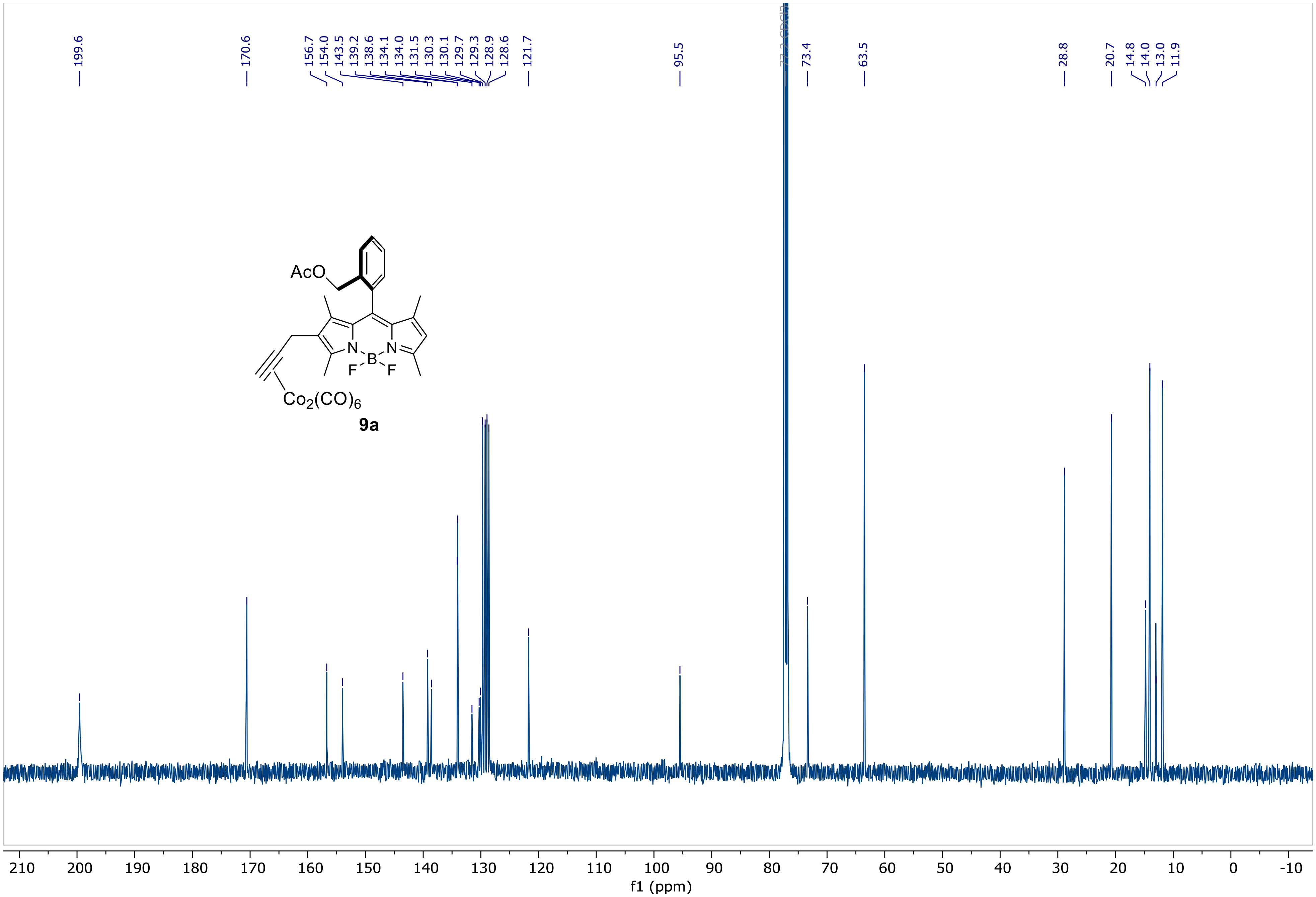

13C NMR (125 MHz, CDCl3)

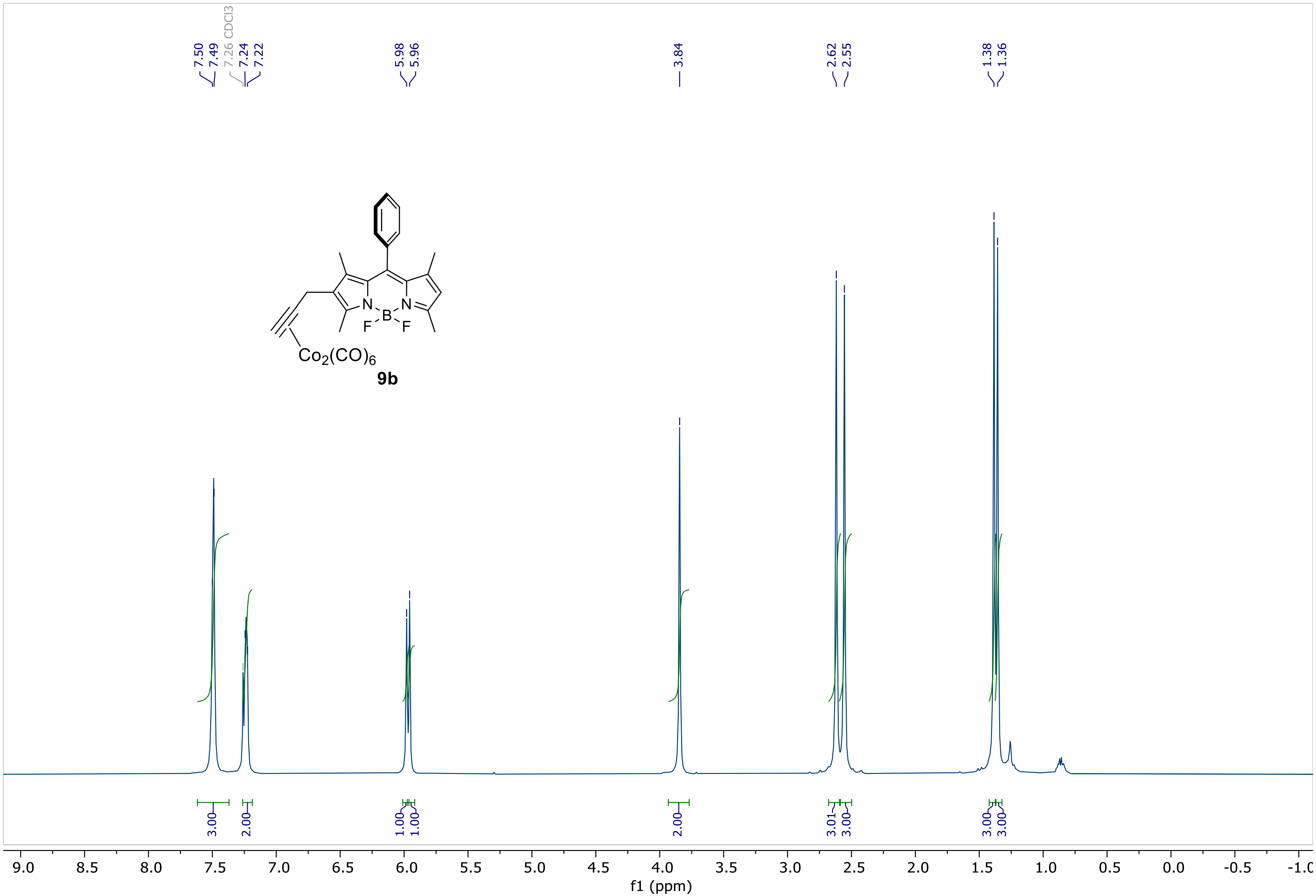

1H NMR (400 MHz, CDCl3)

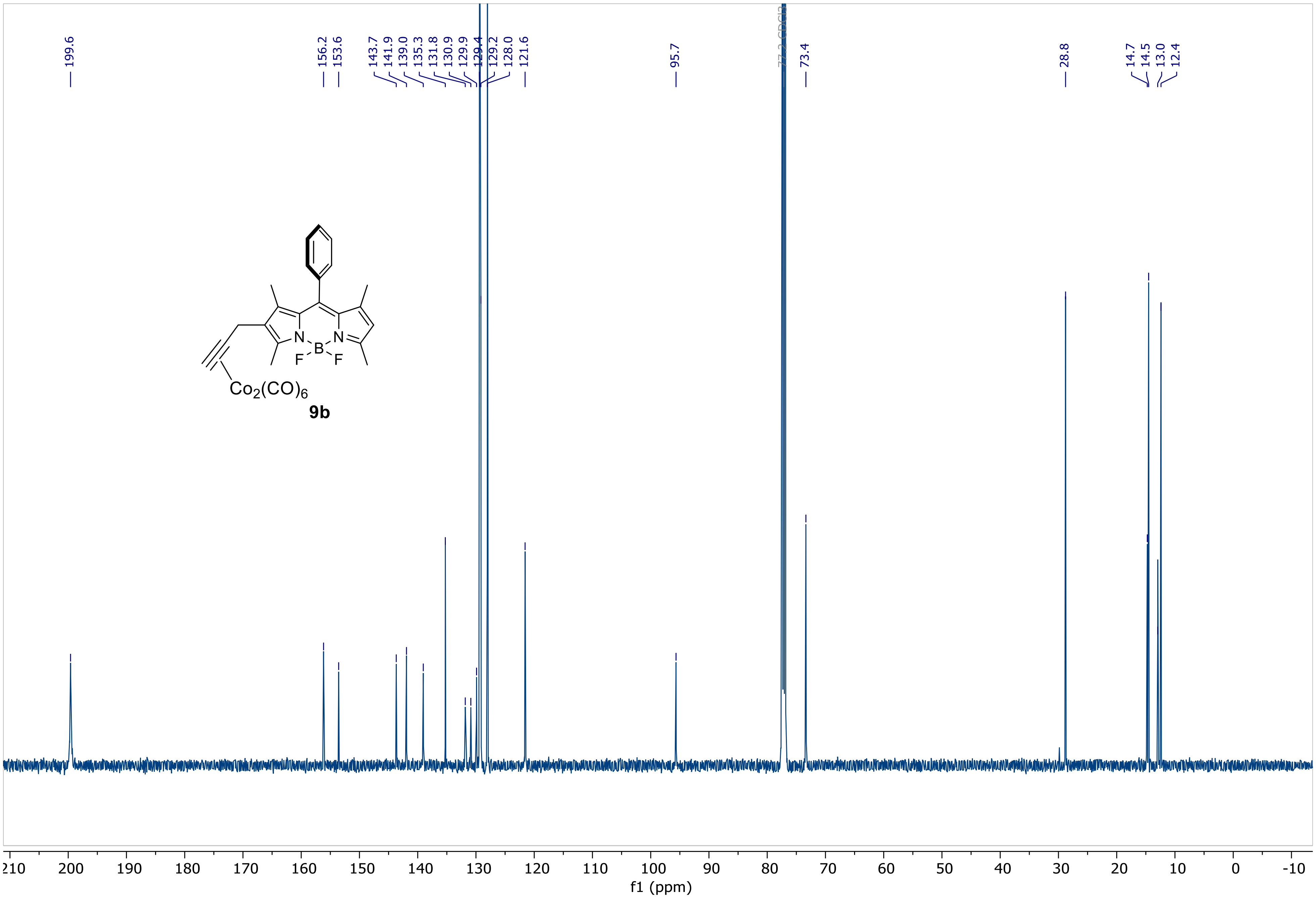

13C {1H} NMR (125MHz, CDCl3)

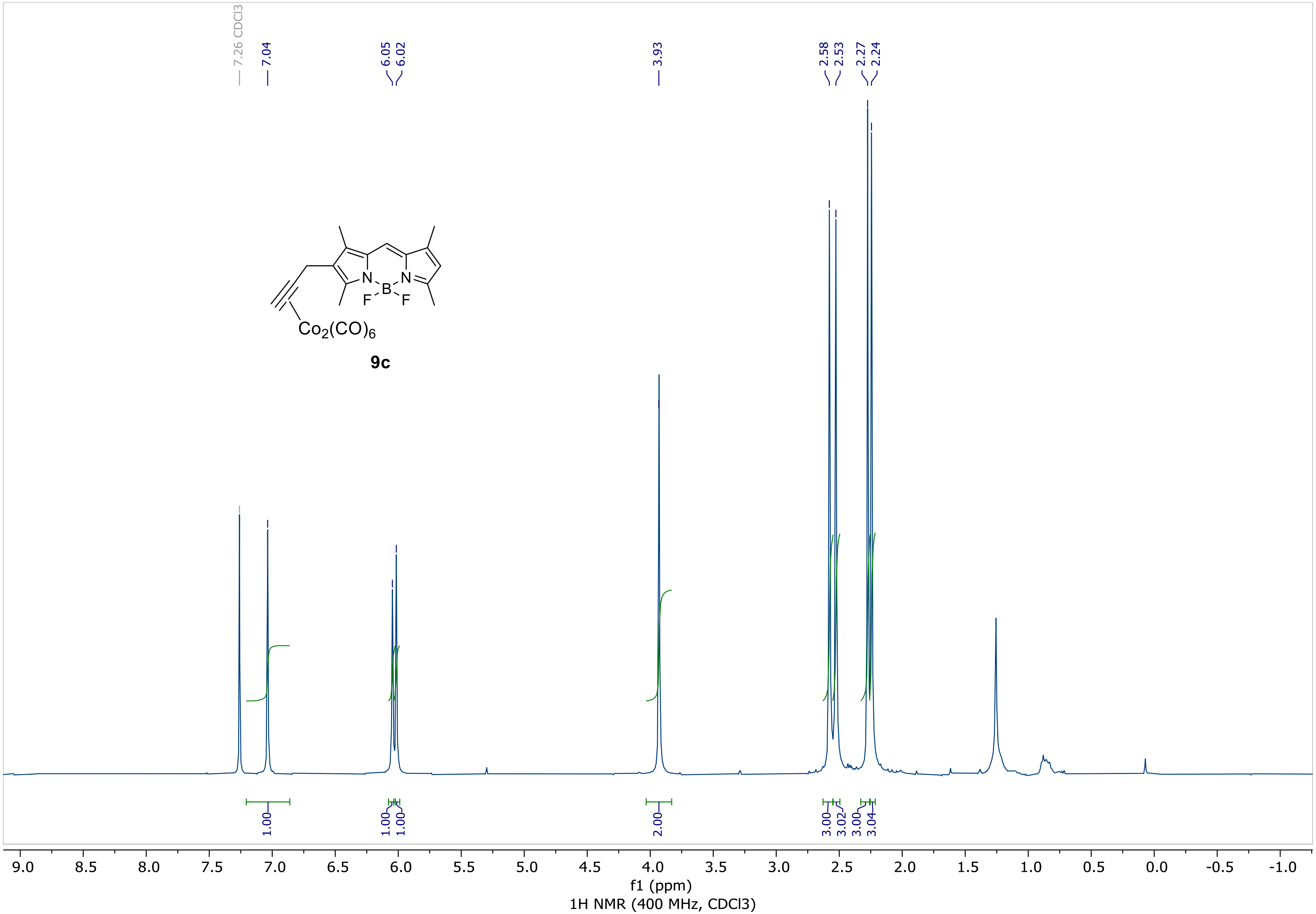
7.26 CDCl3
7.04
6.05
6.02
3.93
2.58
2.53
2.27
2.24
Co2(CO)6
N
B
F
9c
1.00
1.00
1.00
2.00
3.00
3.02
3.00
3.04
9.0 8.5 8.0 7.5 7.0 6.5 6.0 5.5 5.0 4.5 4.0 3.5 3.0 2.5 2.0 1.5 1.0 0.5 0.0 -0.5 -1.0
f1 (ppm)
1H NMR (400 MHz, CDCl3)

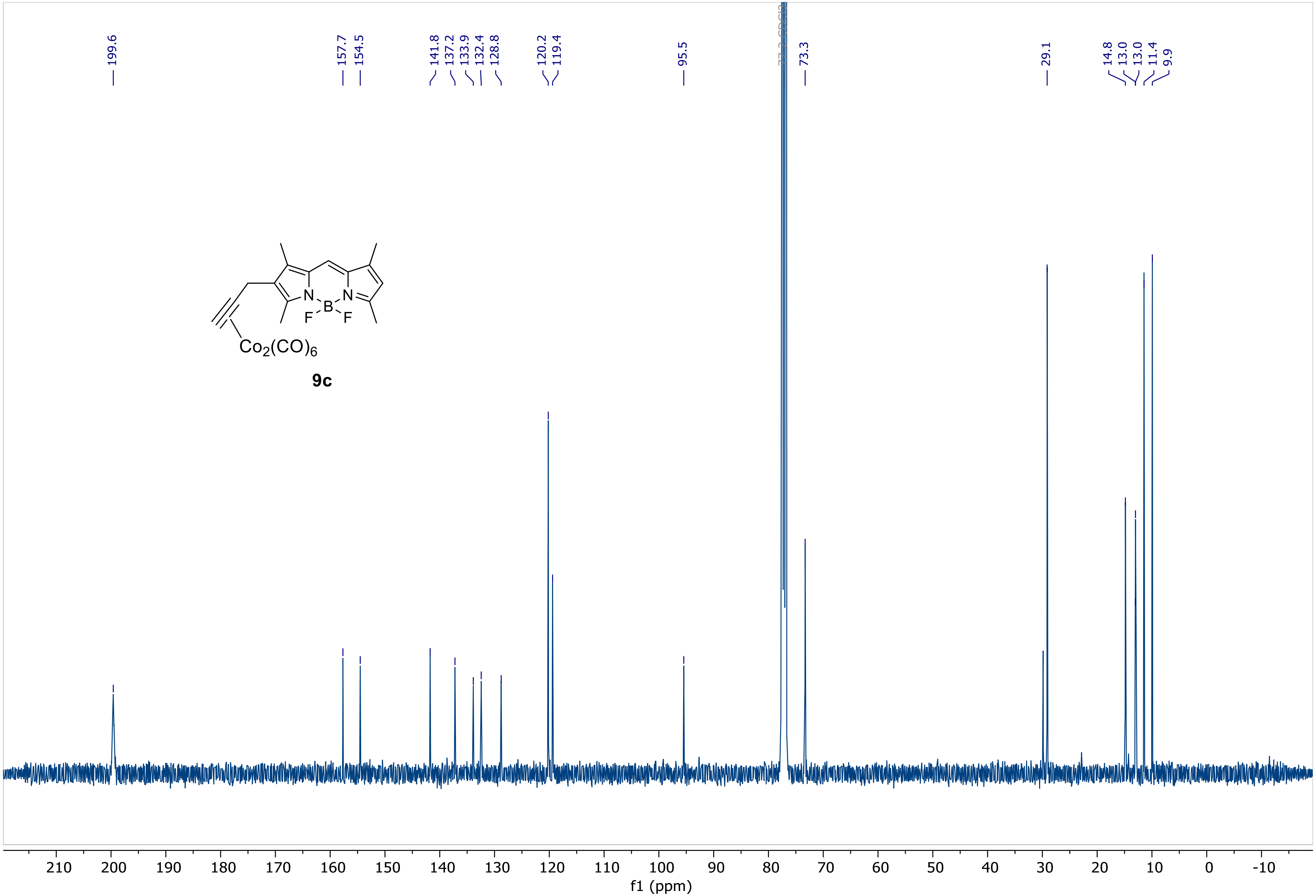

13C {1H} NMR (125 MHz, CDCl3)

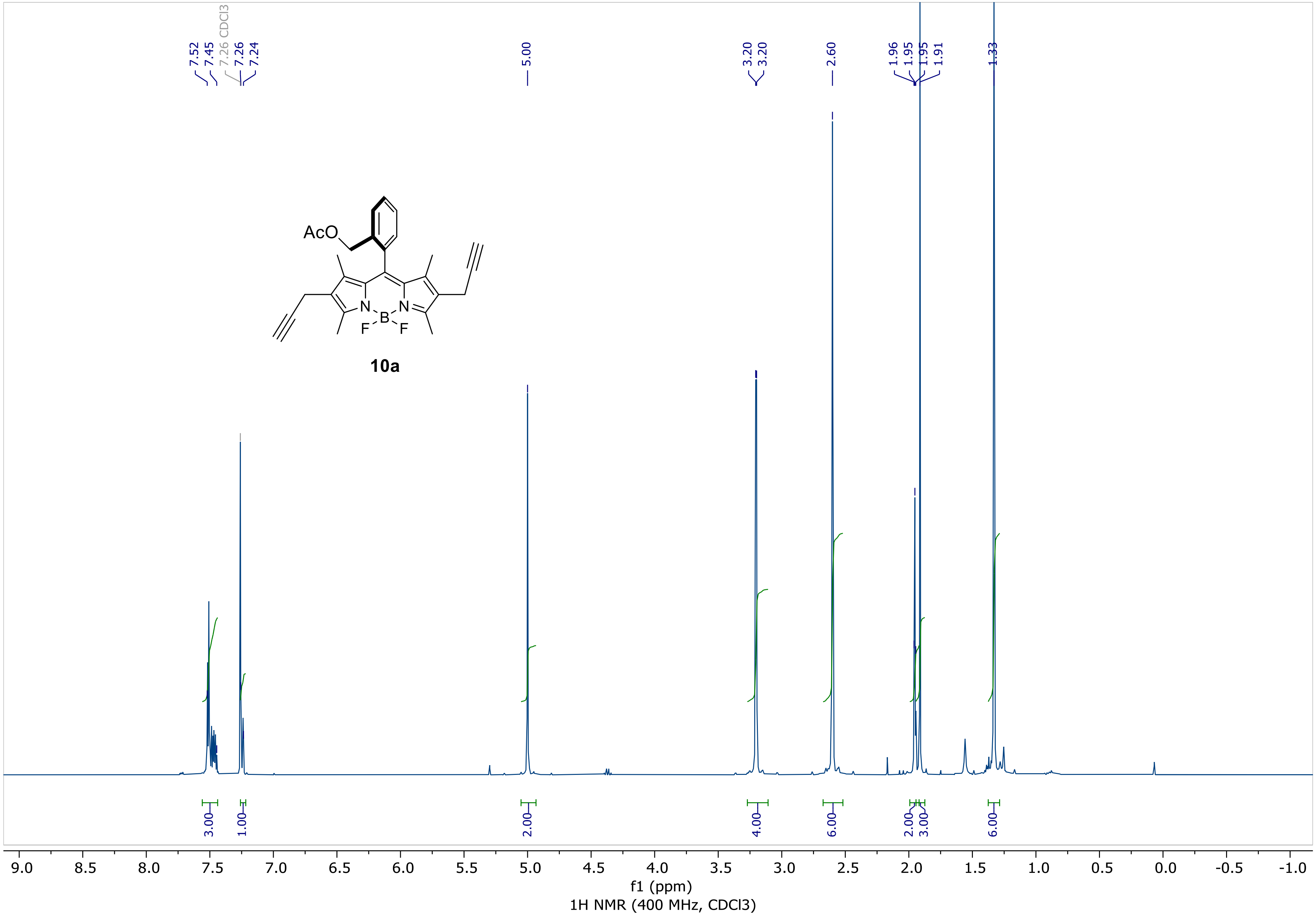
7.52
7.45
7.26 CDCl3
7.26
7.24
5.00
3.20
3.20
2.60
1.96
1.95
1.95
1.91
1.33
AcO
N
B
N
F
F
10a
3.00
1.00
2.00
4.00
6.00
2.00
3.00
6.00
9.0 8.5 8.0 7.5 7.0 6.5 6.0 5.5 5.0 4.5 4.0 3.5 3.0 2.5 2.0 1.5 1.0 0.5 0.0 -0.5 -1.0
f1 (ppm)
1H NMR (400 MHz, CDCl3)

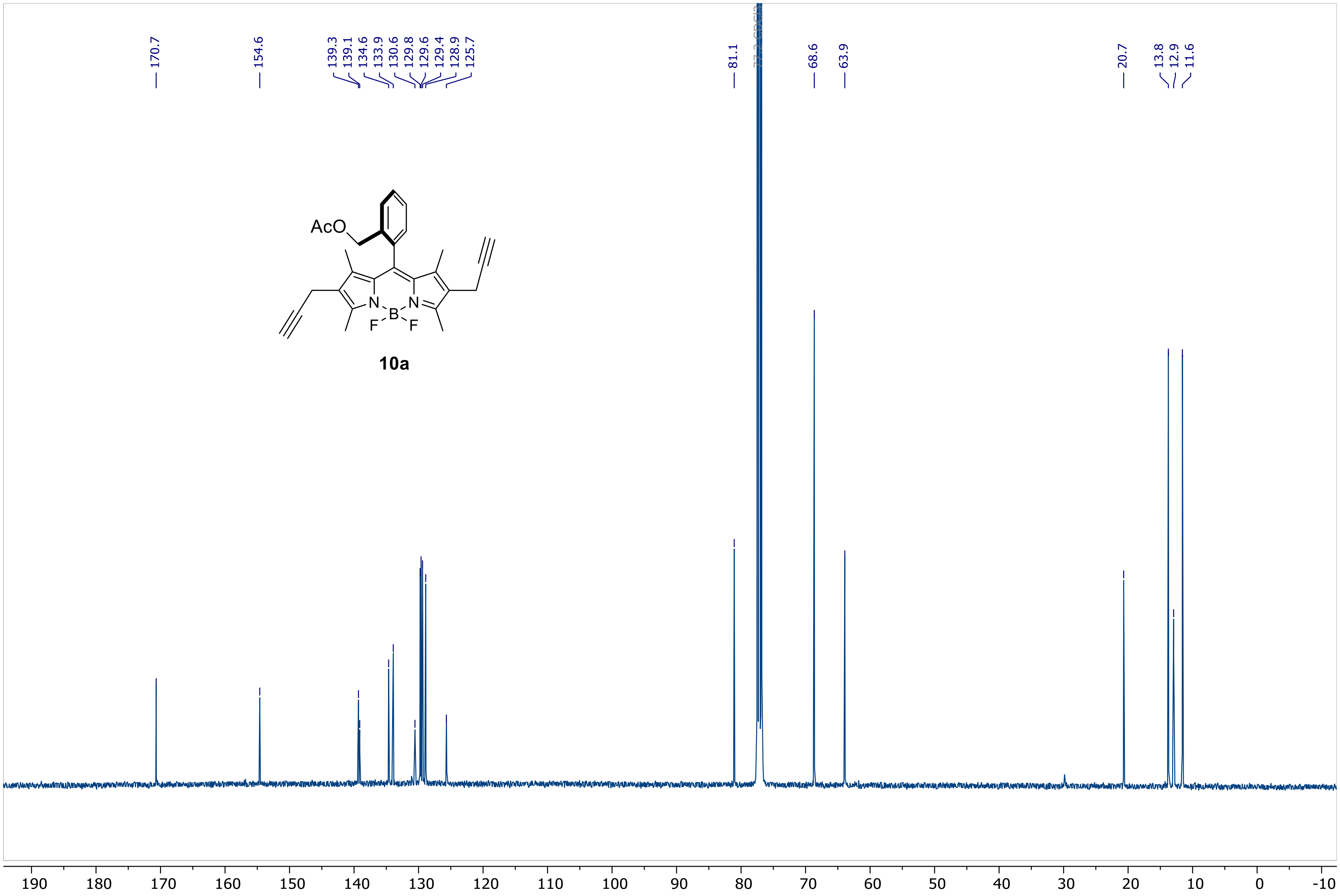

13C {1H} NMR (125 MHz, CDCl3)

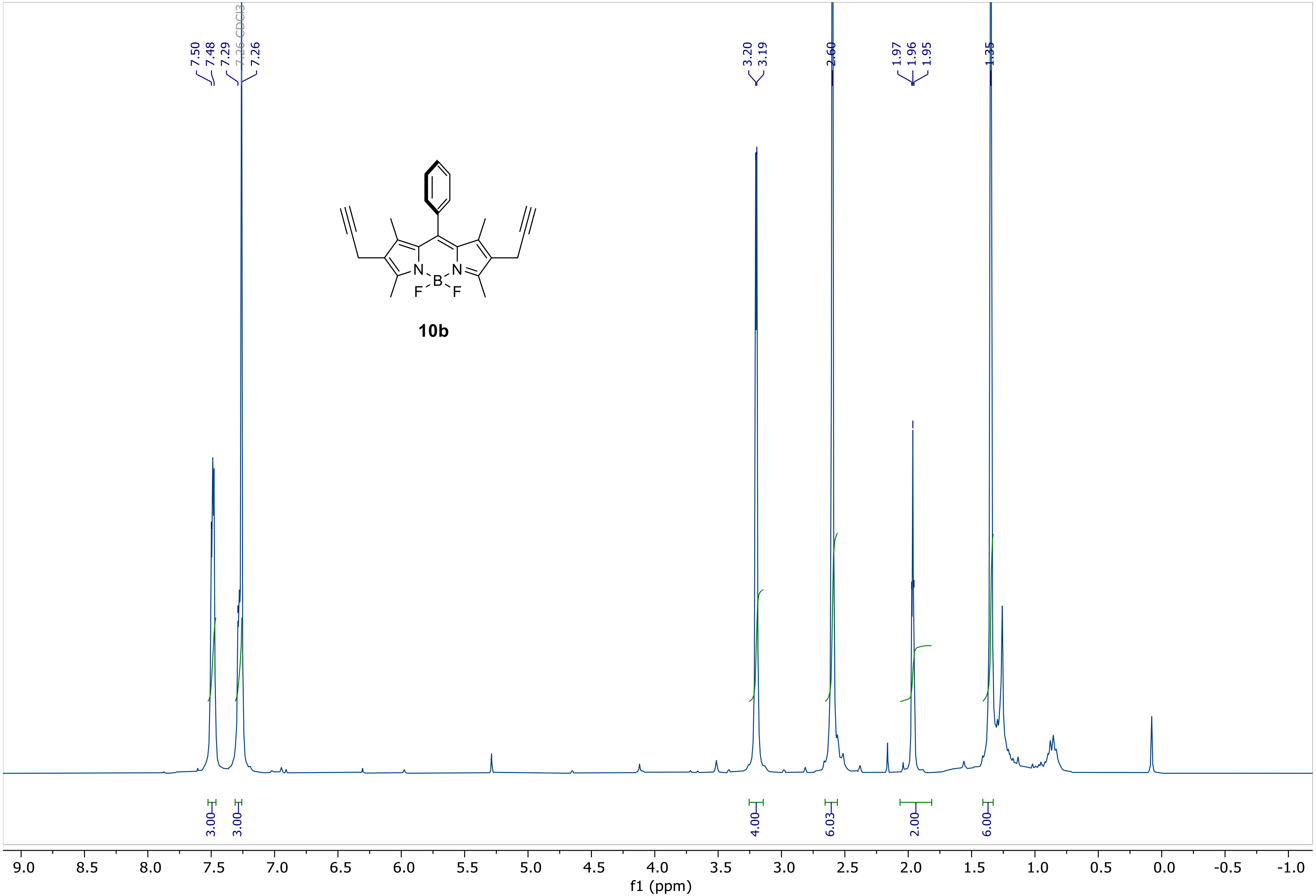

1H NMR (400 MHz, CDCl3)

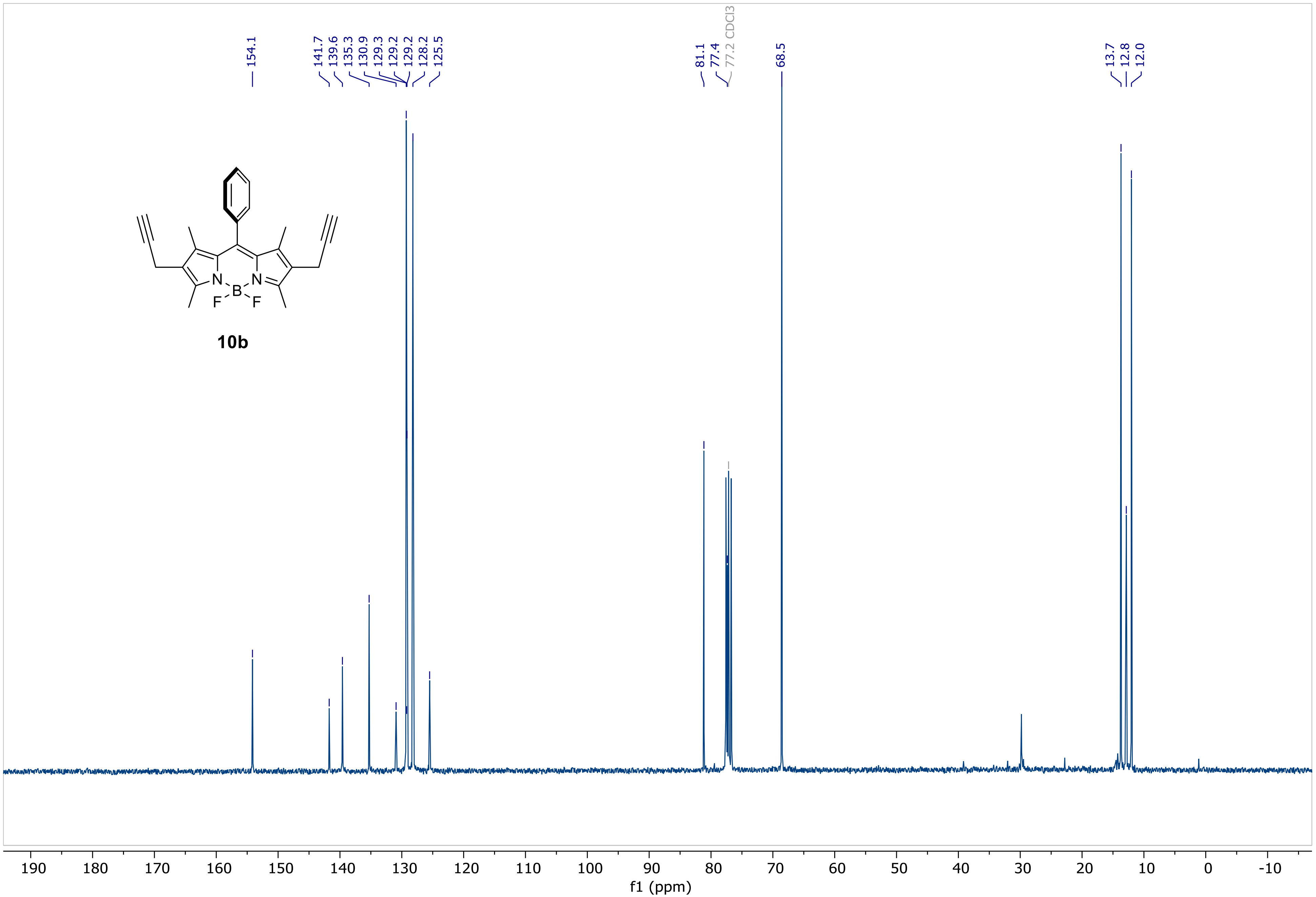

13C {1H} NMR (125 MHz, CDCl3)

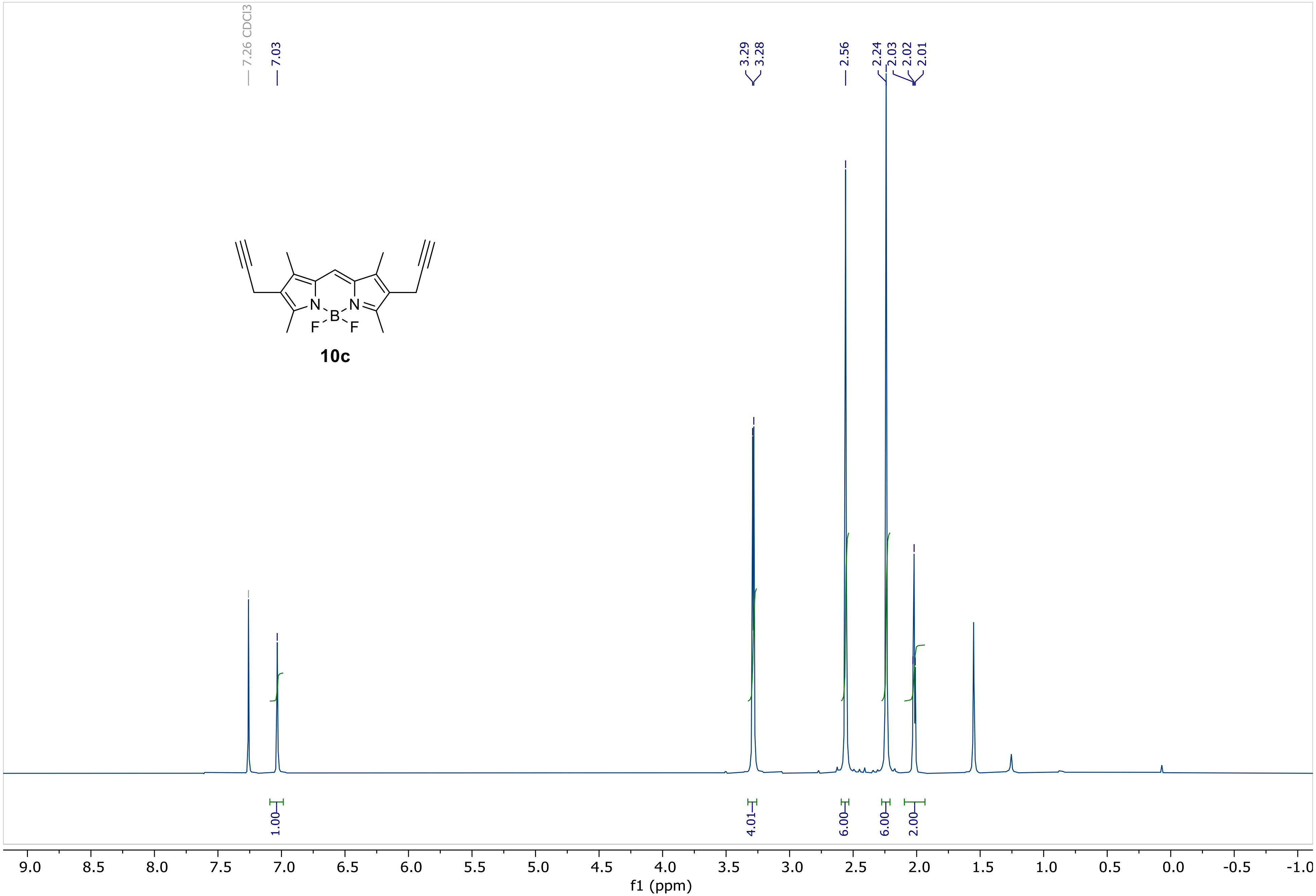

1H NMR (400 MHz, CDCl3)

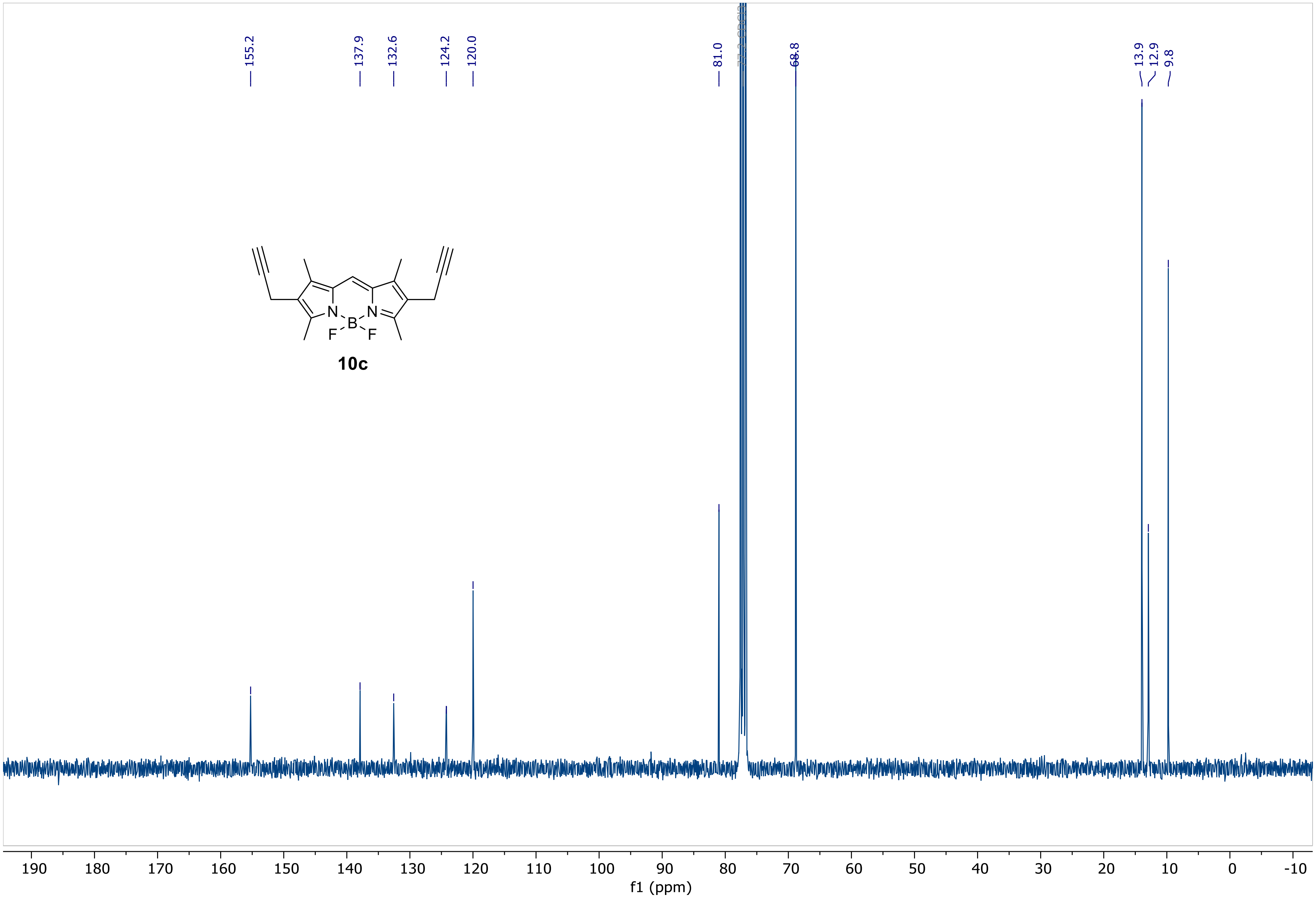

13C {1H} NMR (125 MHz, CDCl3)

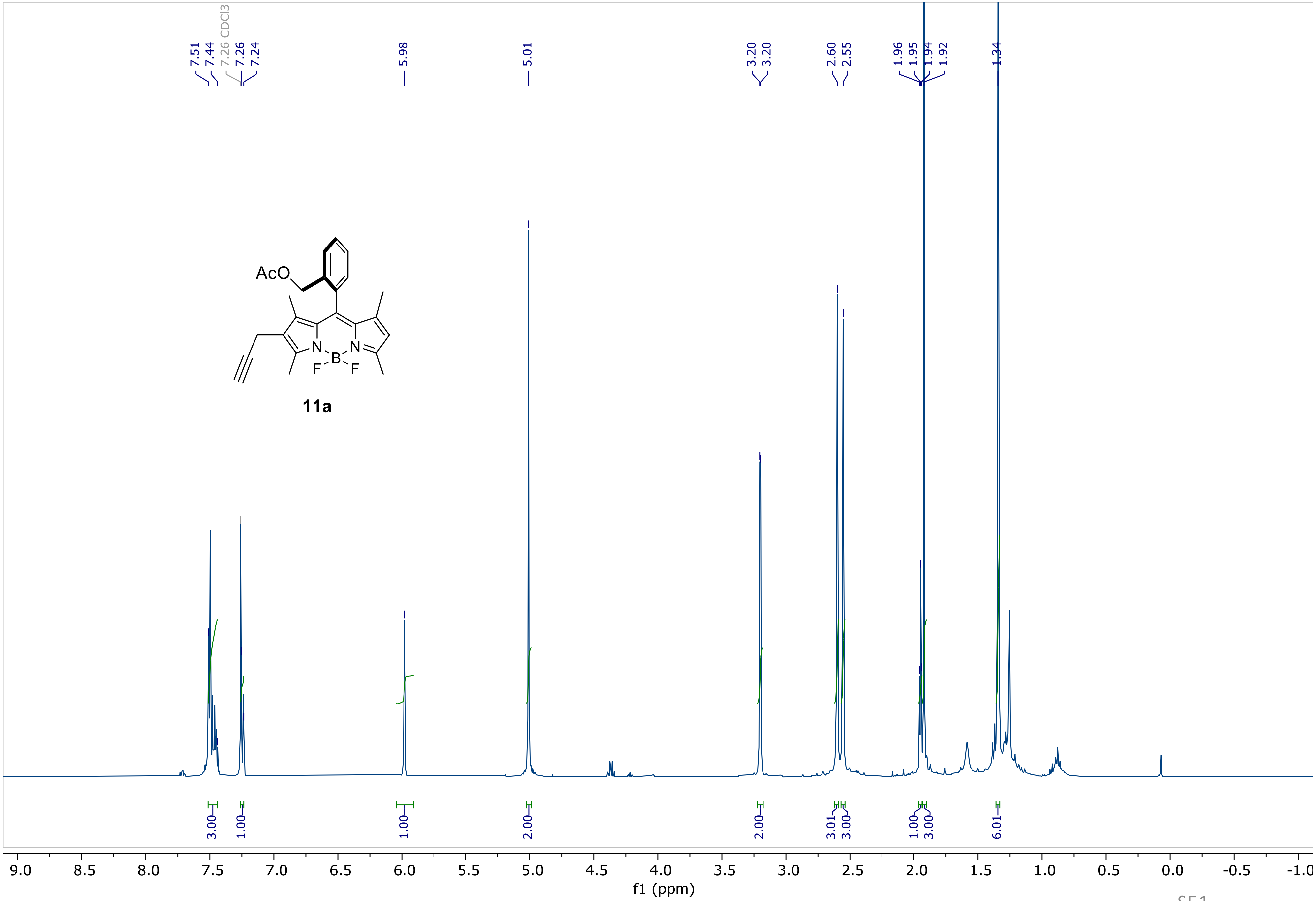

1H NMR (400 MHz, CDCl3)

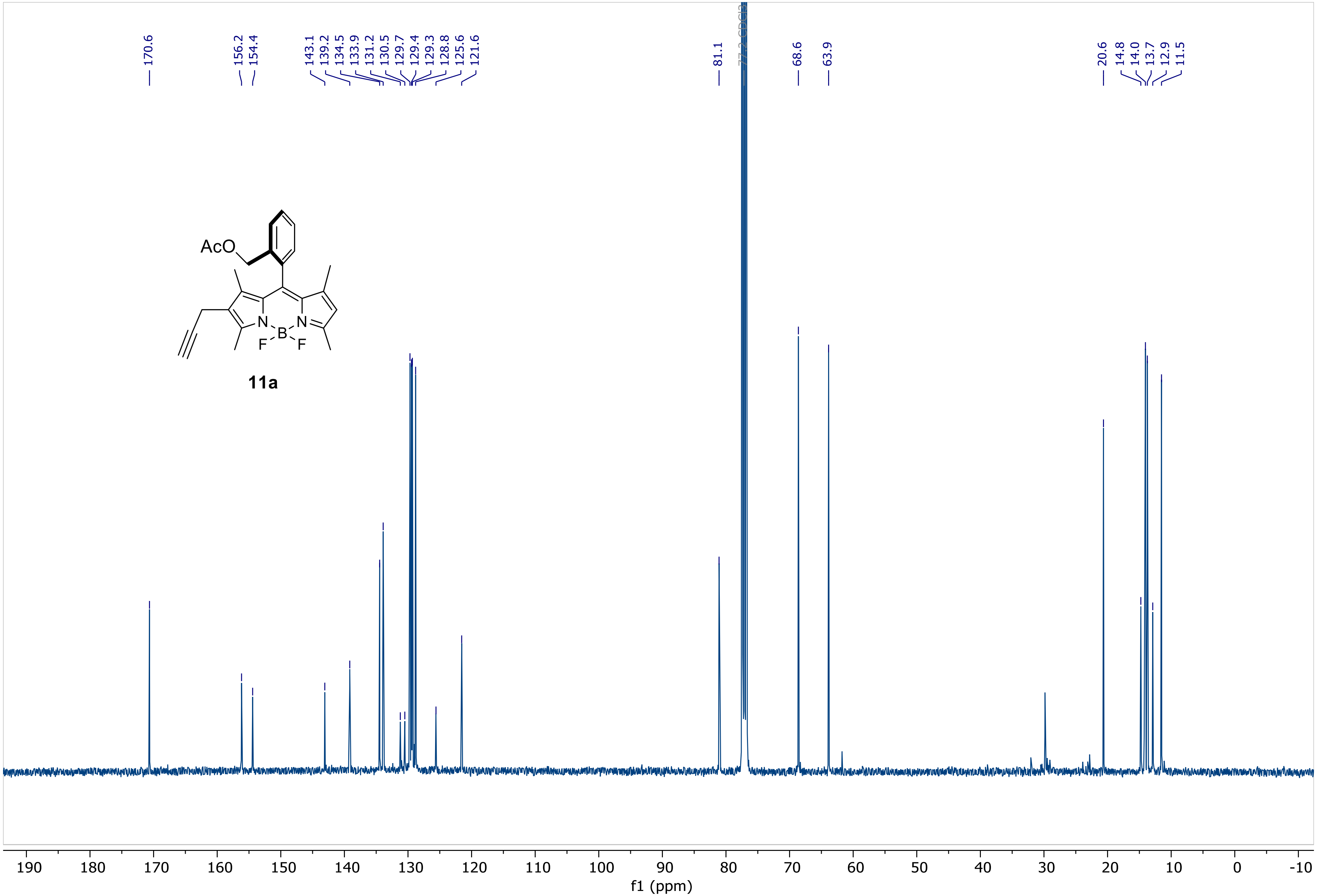

13C NMR (125 MHz, CDCl3)

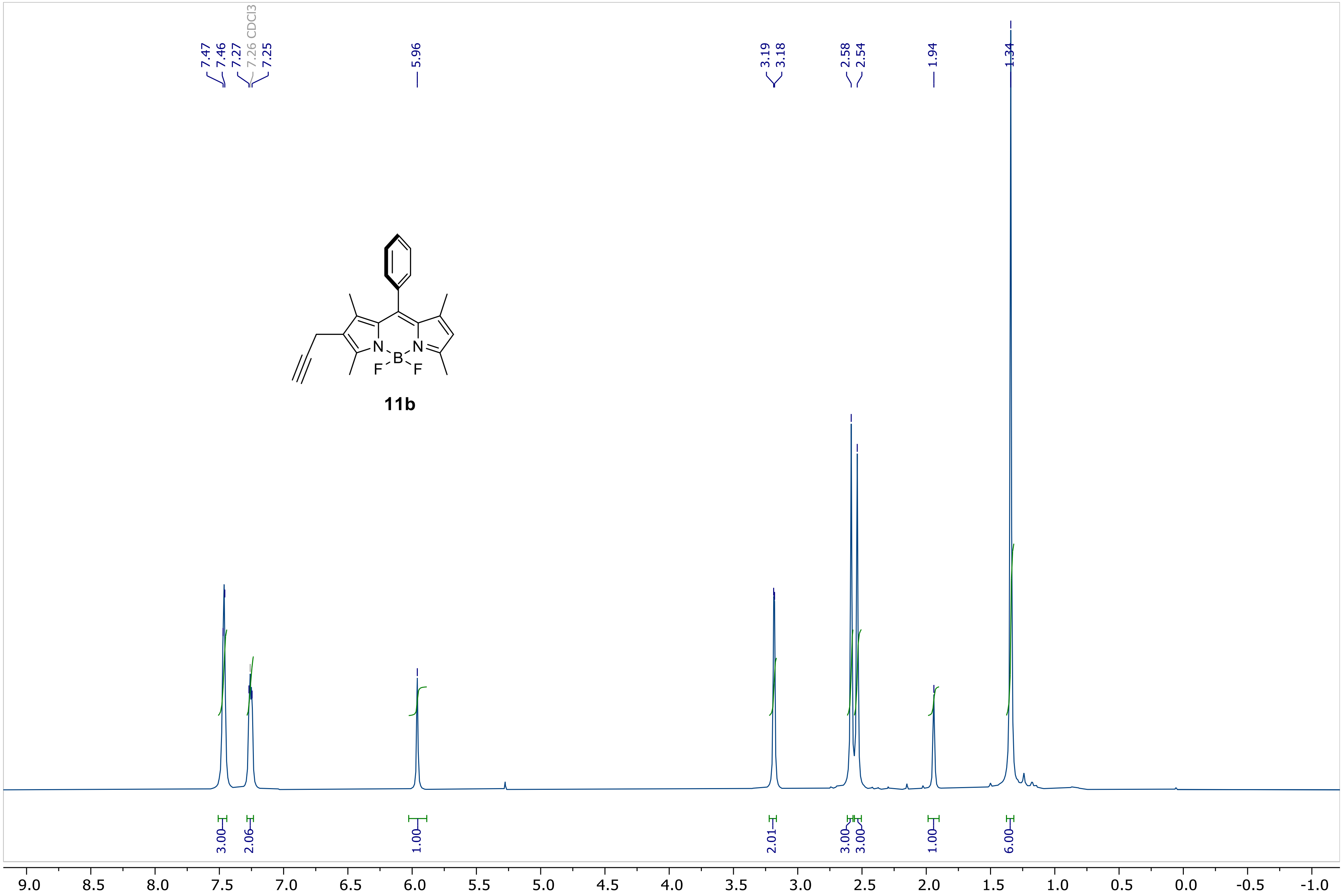

1H NMR (400 MHz, CDCl3)

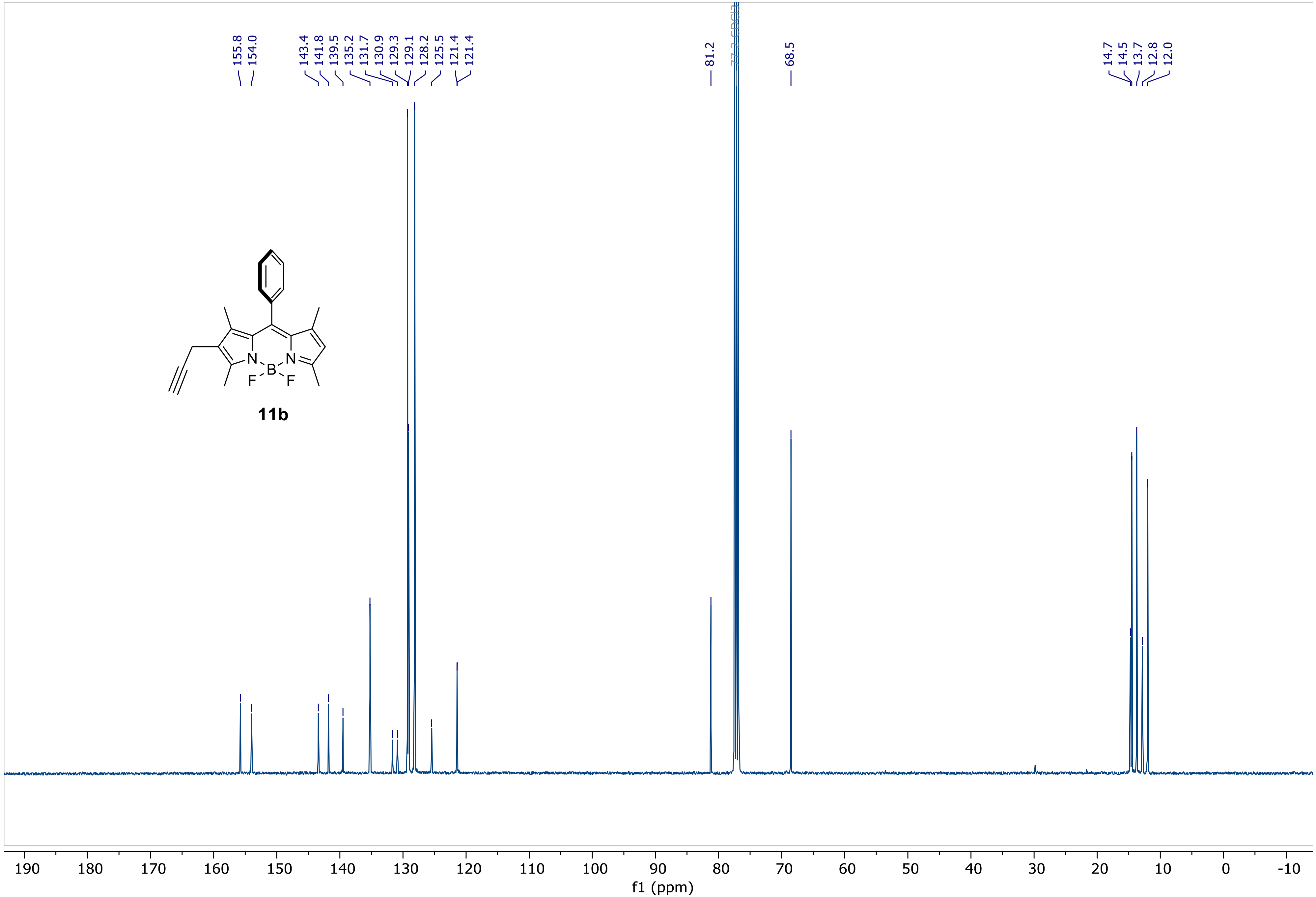

13C {1H} NMR (125 MHz, $CDCl_3$)

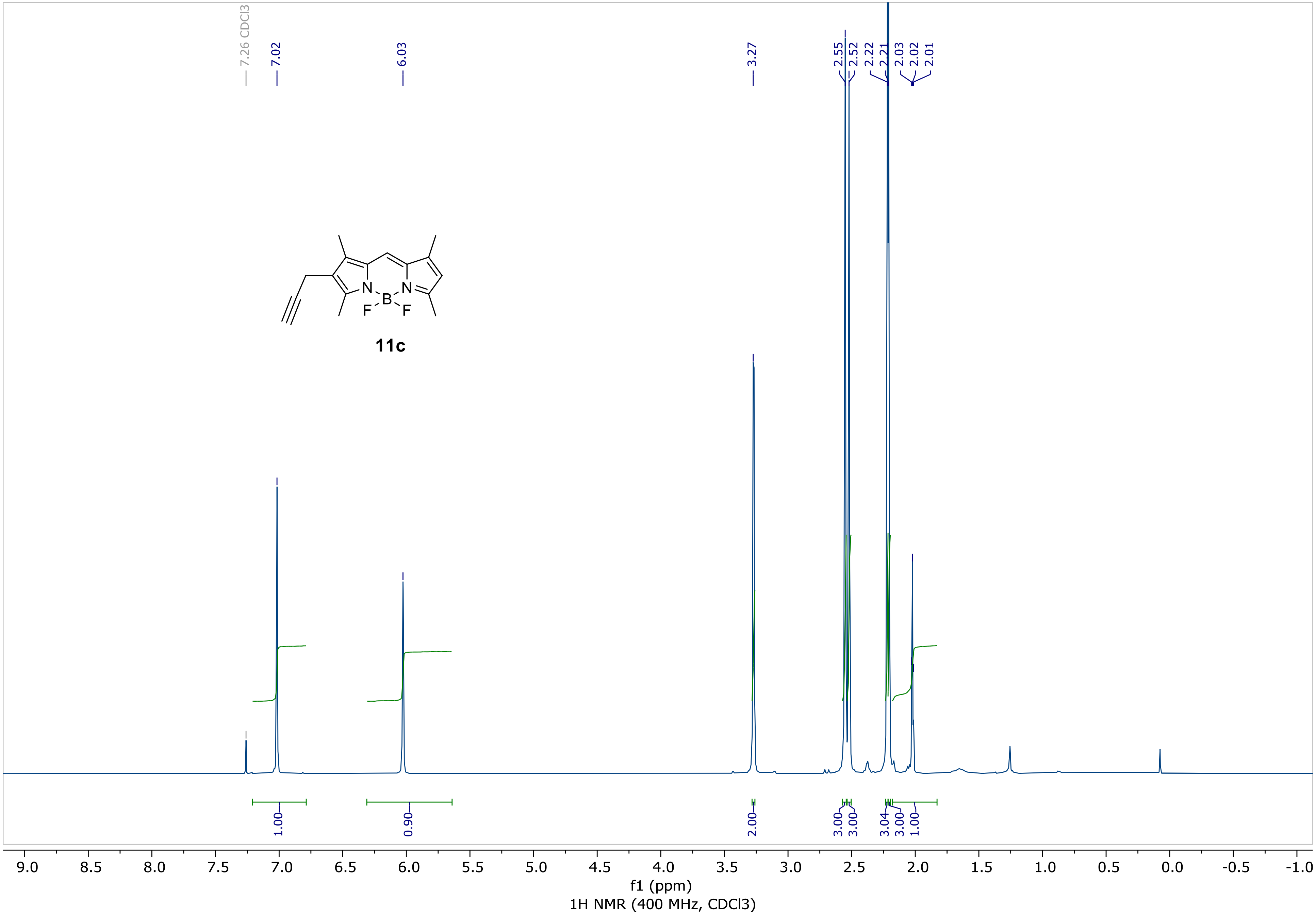
7.26 CDCl3
7.02
6.03
3.27
2.55
2.52
2.22
2.21
2.03
2.02
2.01
N
B
N
F
F
11c
1.00
0.90
2.00
3.00
3.00
3.04
3.00
1.00
9.0 8.5 8.0 7.5 7.0 6.5 6.0 5.5 5.0 4.5 4.0 3.5 3.0 2.5 2.0 1.5 1.0 0.5 0.0 -0.5 -1.0
f1 (ppm)
1H NMR (400 MHz, CDCl3)

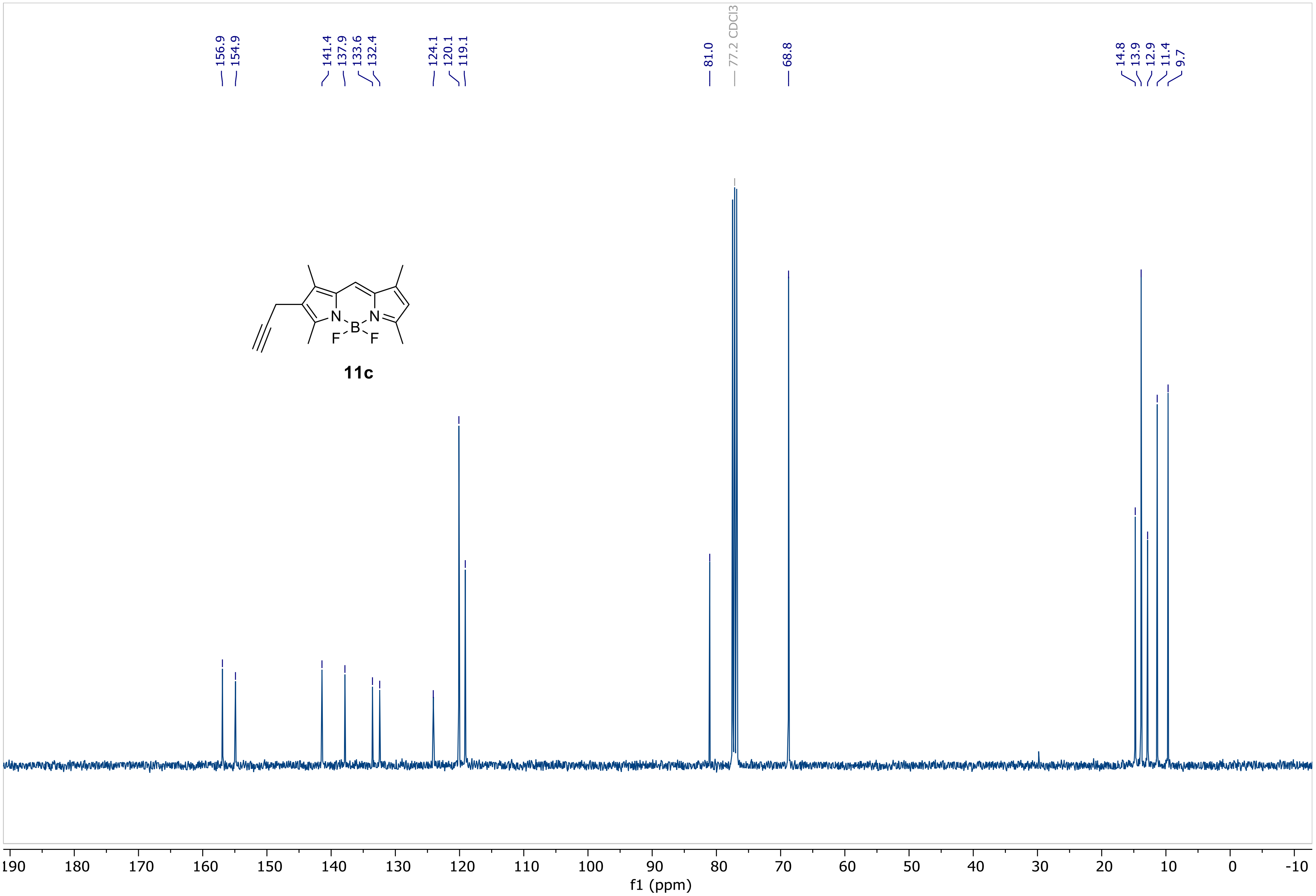

13C {1H} NMR (125 MHz, CDCl3)

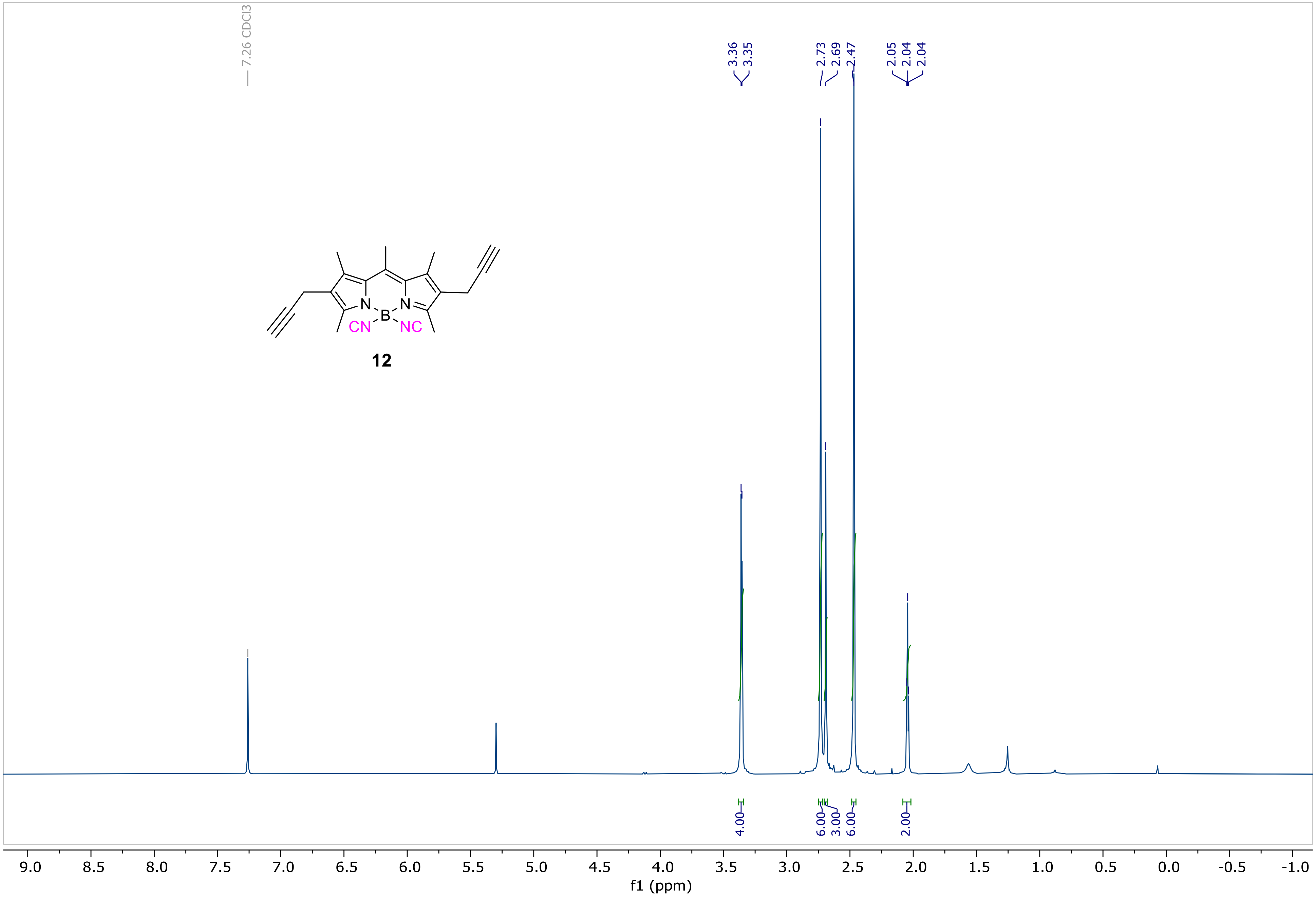

1H NMR (400 MHz, CDCl3)

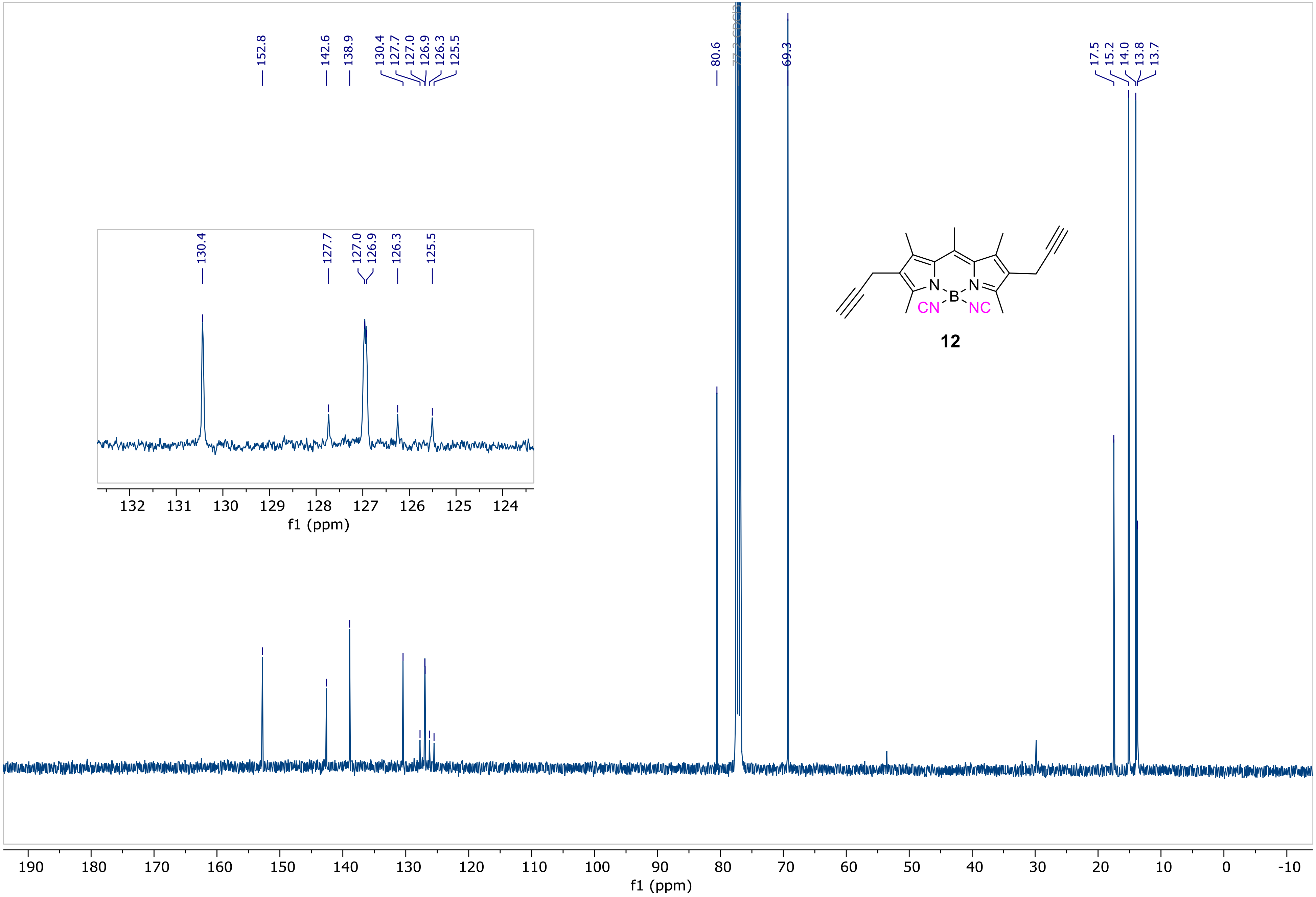

13C {1H} NMR (125 MHz, CDCl3)

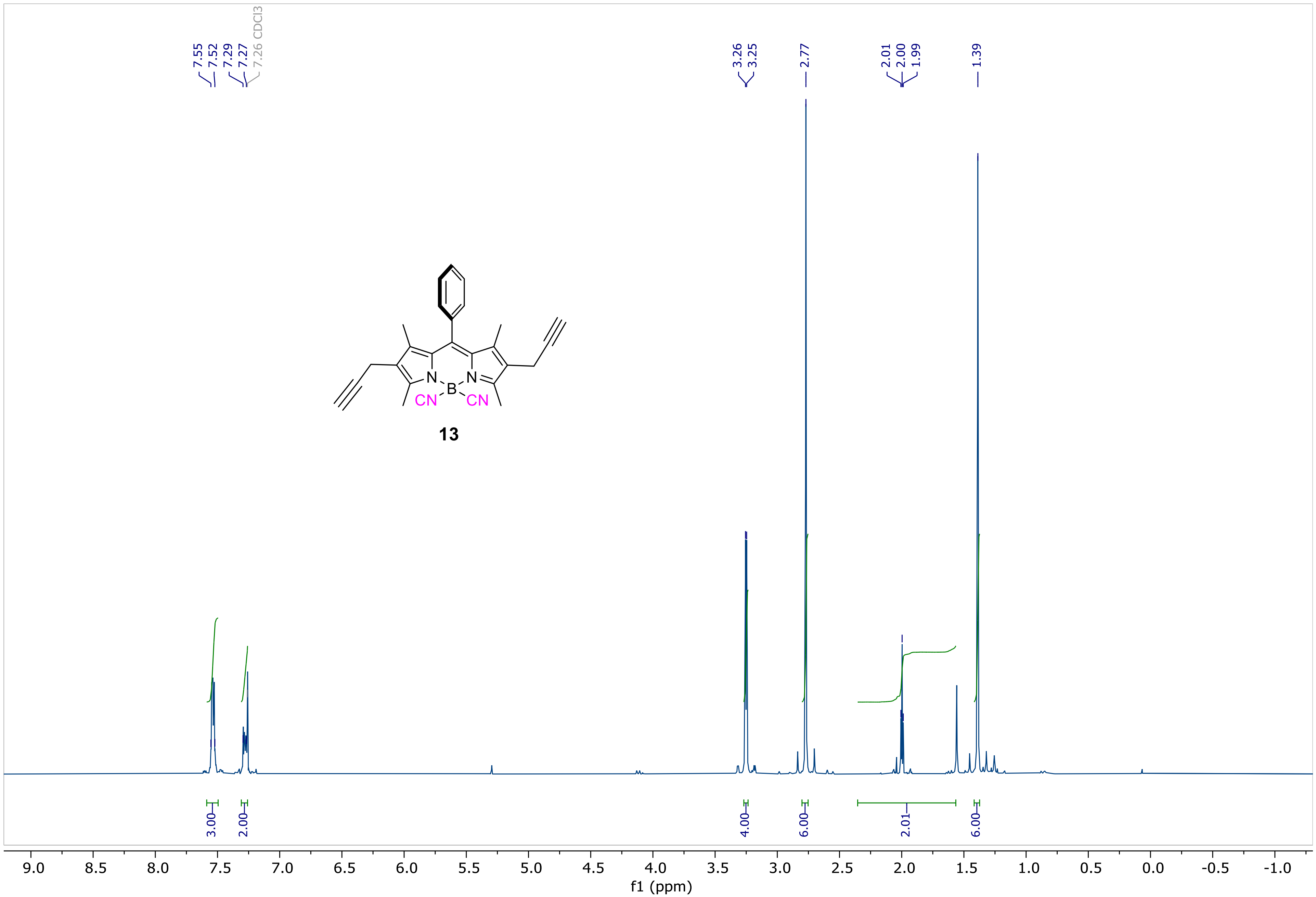

1H NMR (400 MHz, CDCl3)

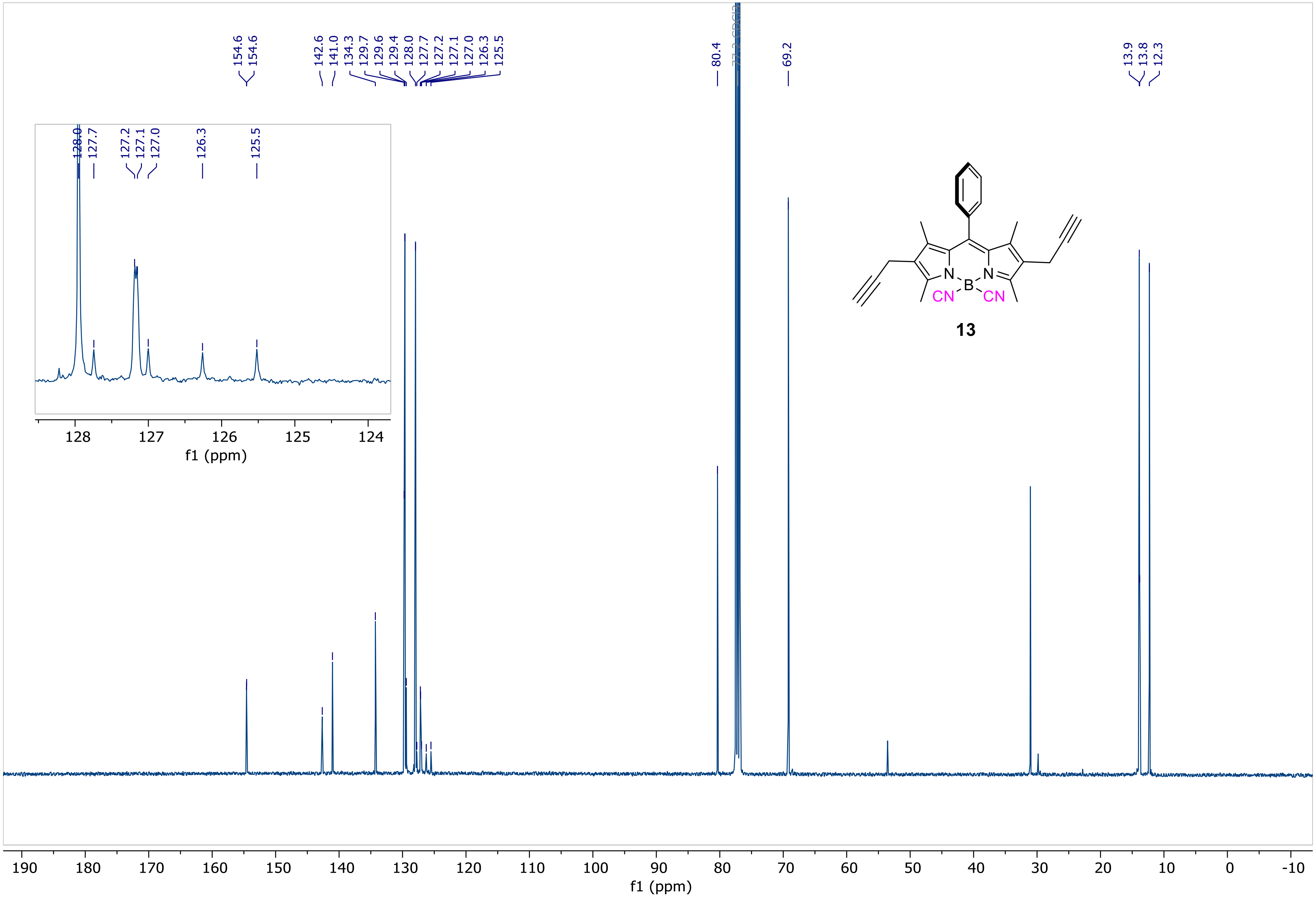

13C {1H} NMR (125 MHz, CDCl3)

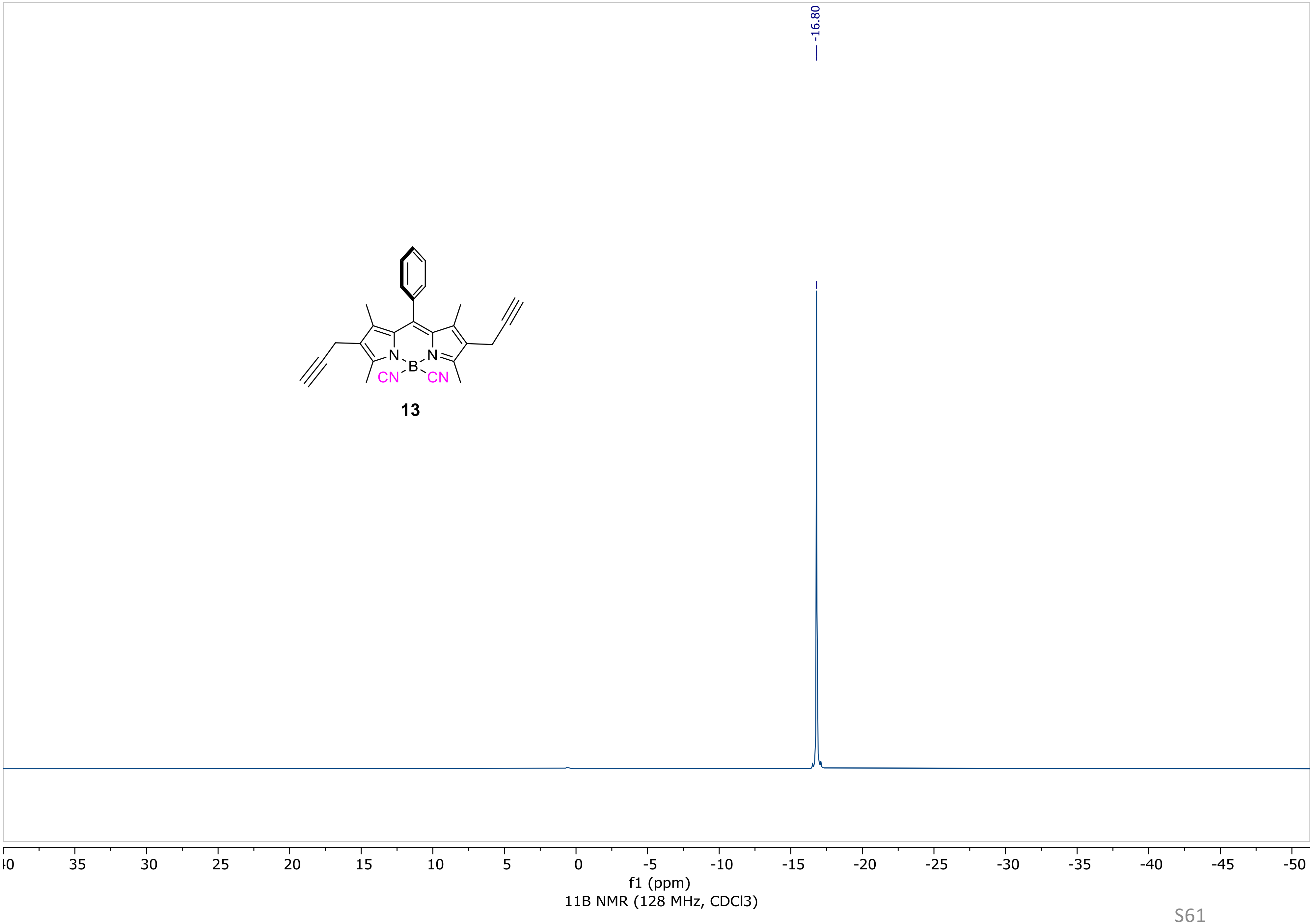
-16.80
CN
CN
N
B
N
13
f1 (ppm)
11B NMR (128 MHz, CDCl3)

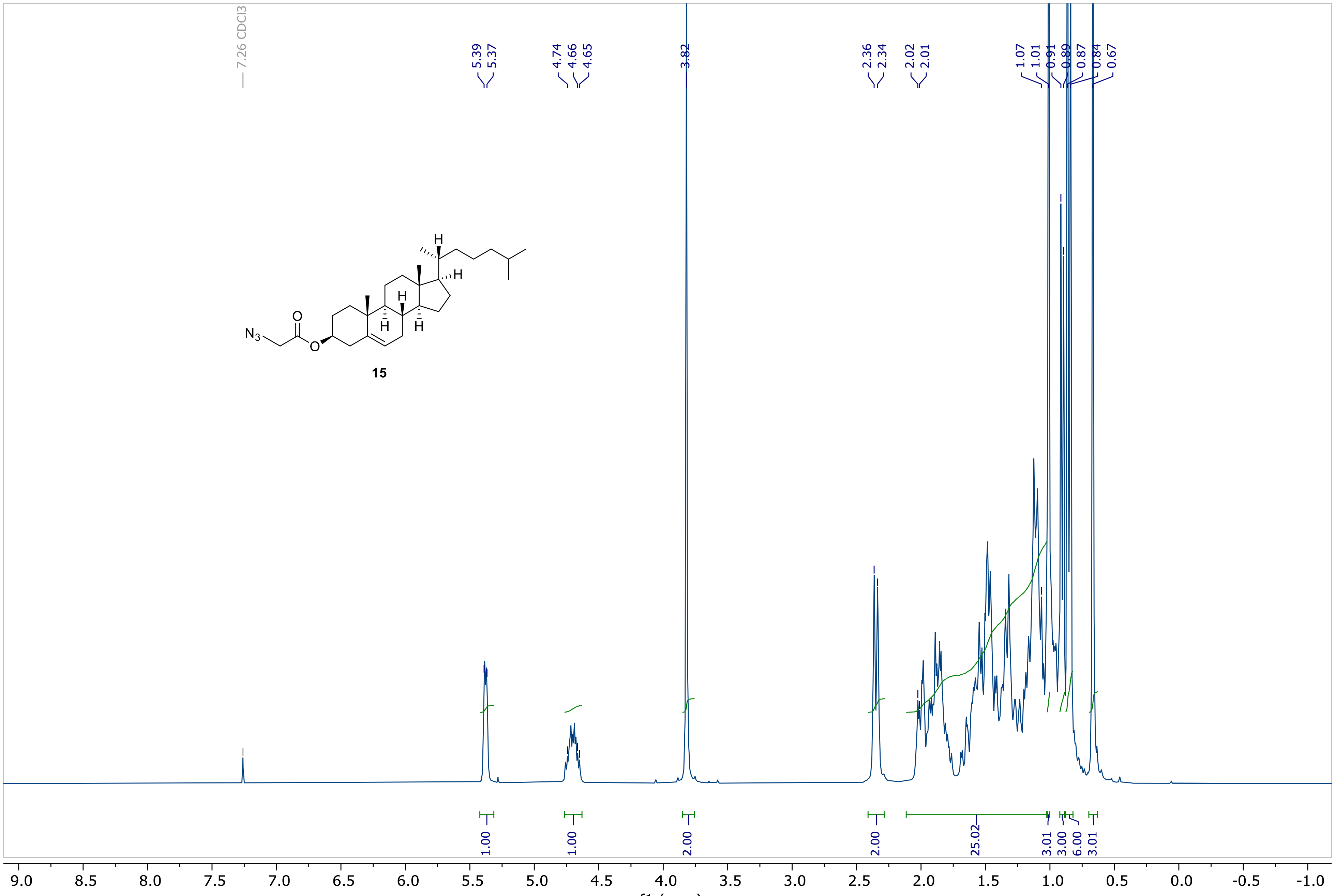

1H NMR (300 MHz, CDCl3)

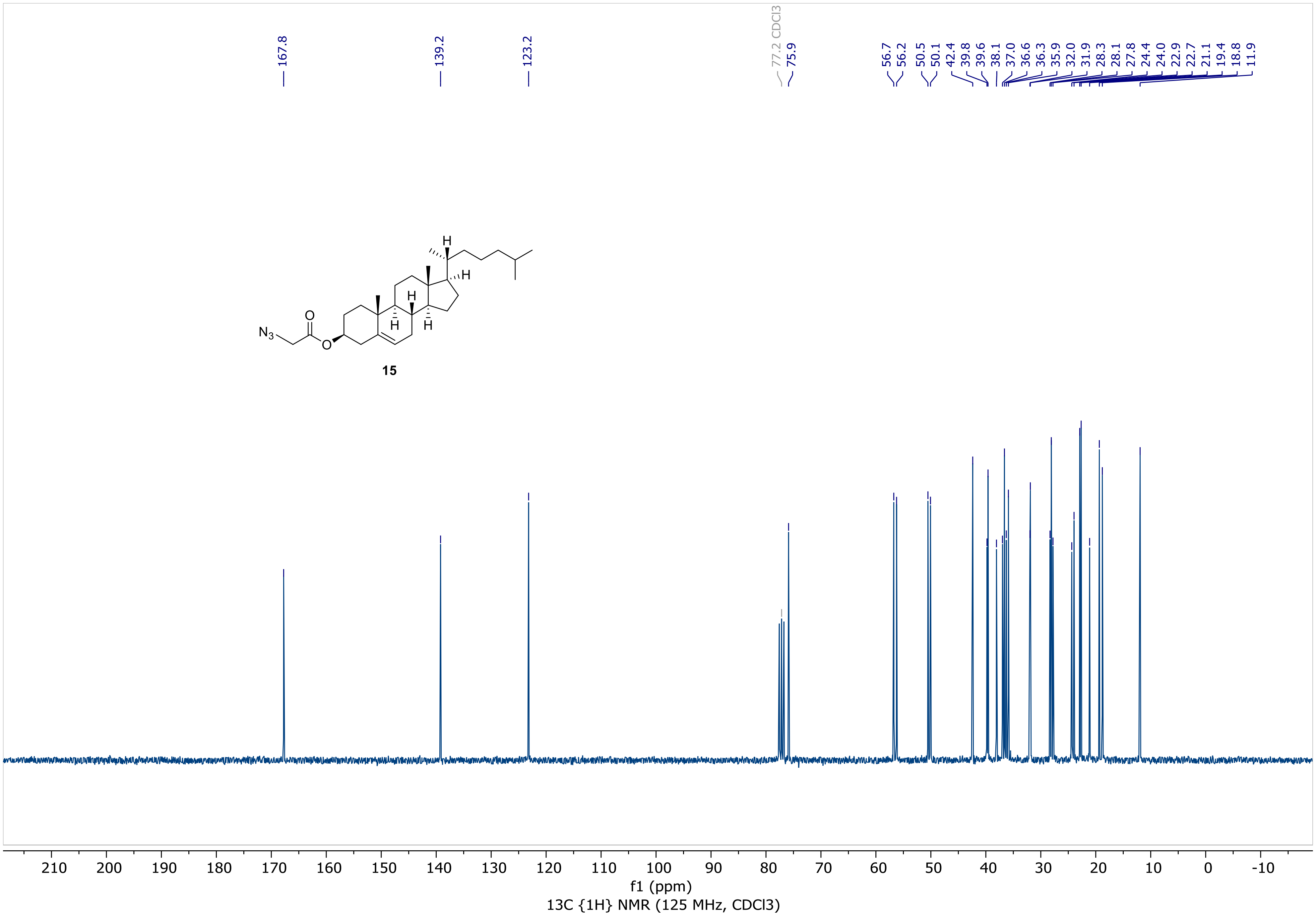
167.8
139.2
123.2
77.2 CDCl3
75.9
56.7
56.2
50.5
50.1
42.4
39.8
39.6
38.1
37.0
36.6
36.3
35.9
32.0
31.9
28.3
28.1
27.8
24.4
24.0
22.9
22.7
21.1
19.4
18.8
11.9
15
f1 (ppm)
13C {1H} NMR (125 MHz, CDCl3)

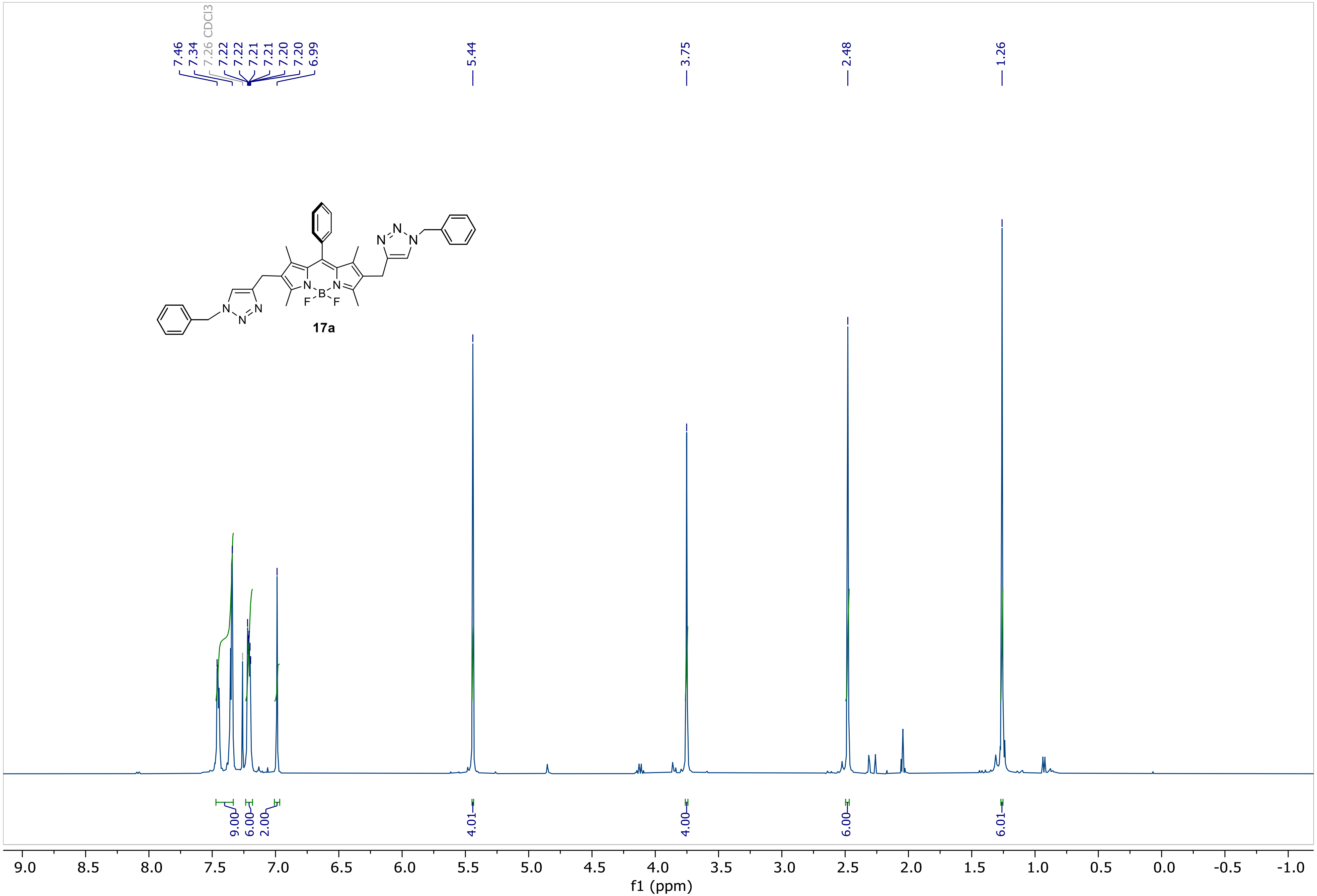

1H NMR (400 MHz, CDCl3)

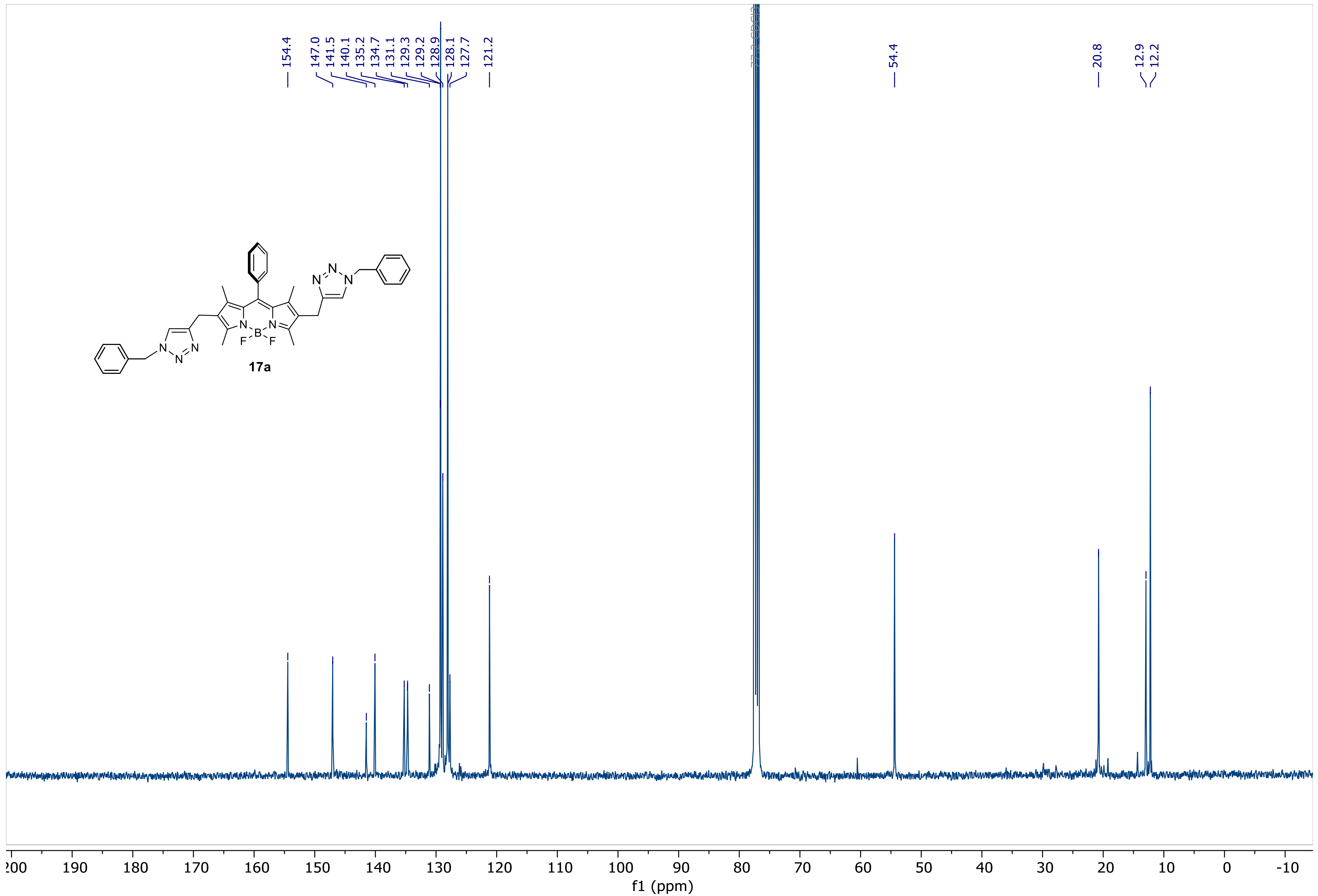

13C {1H} NMR (125 MHz, CDCl3)

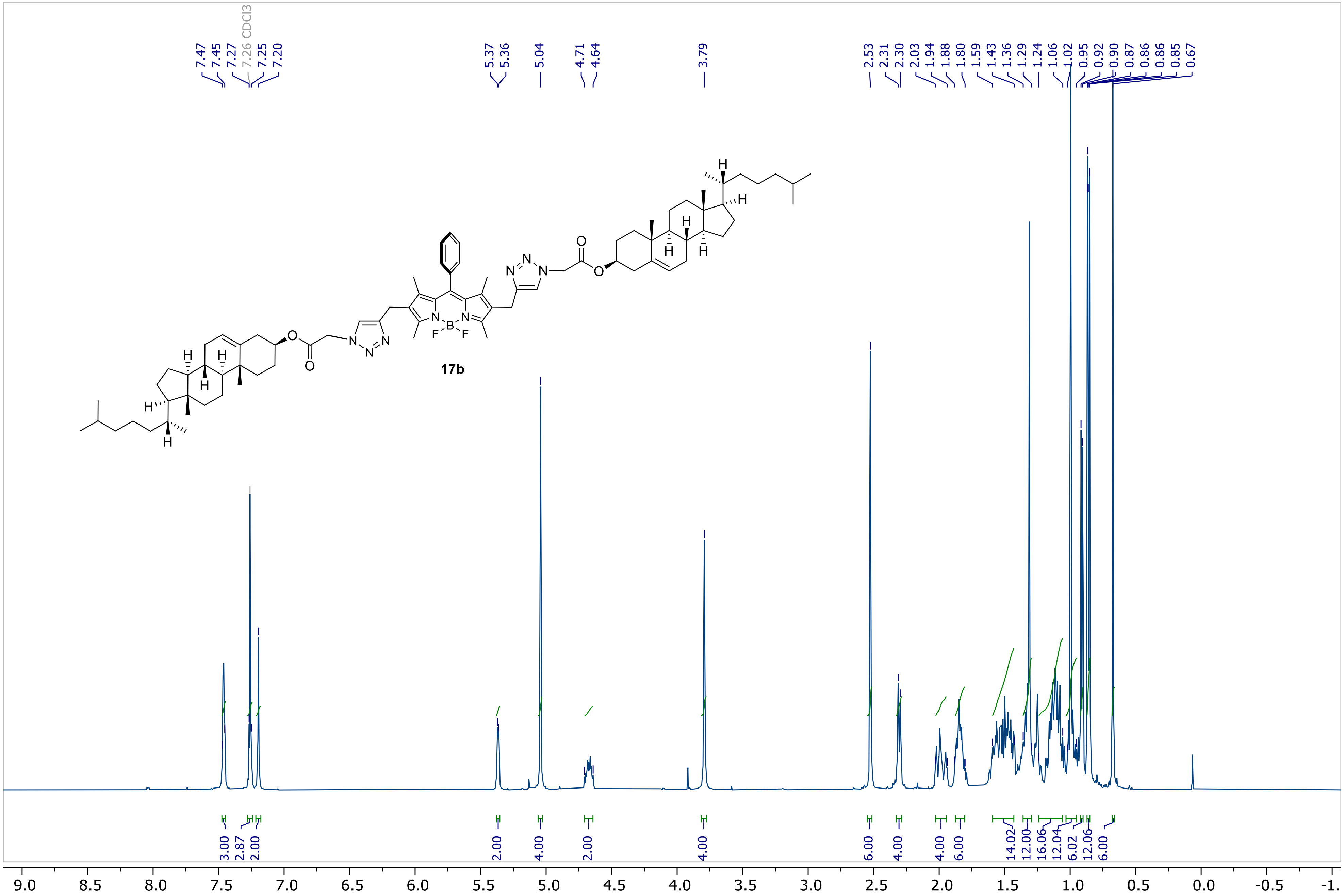

1H NMR (400 MHz, CDCl3)

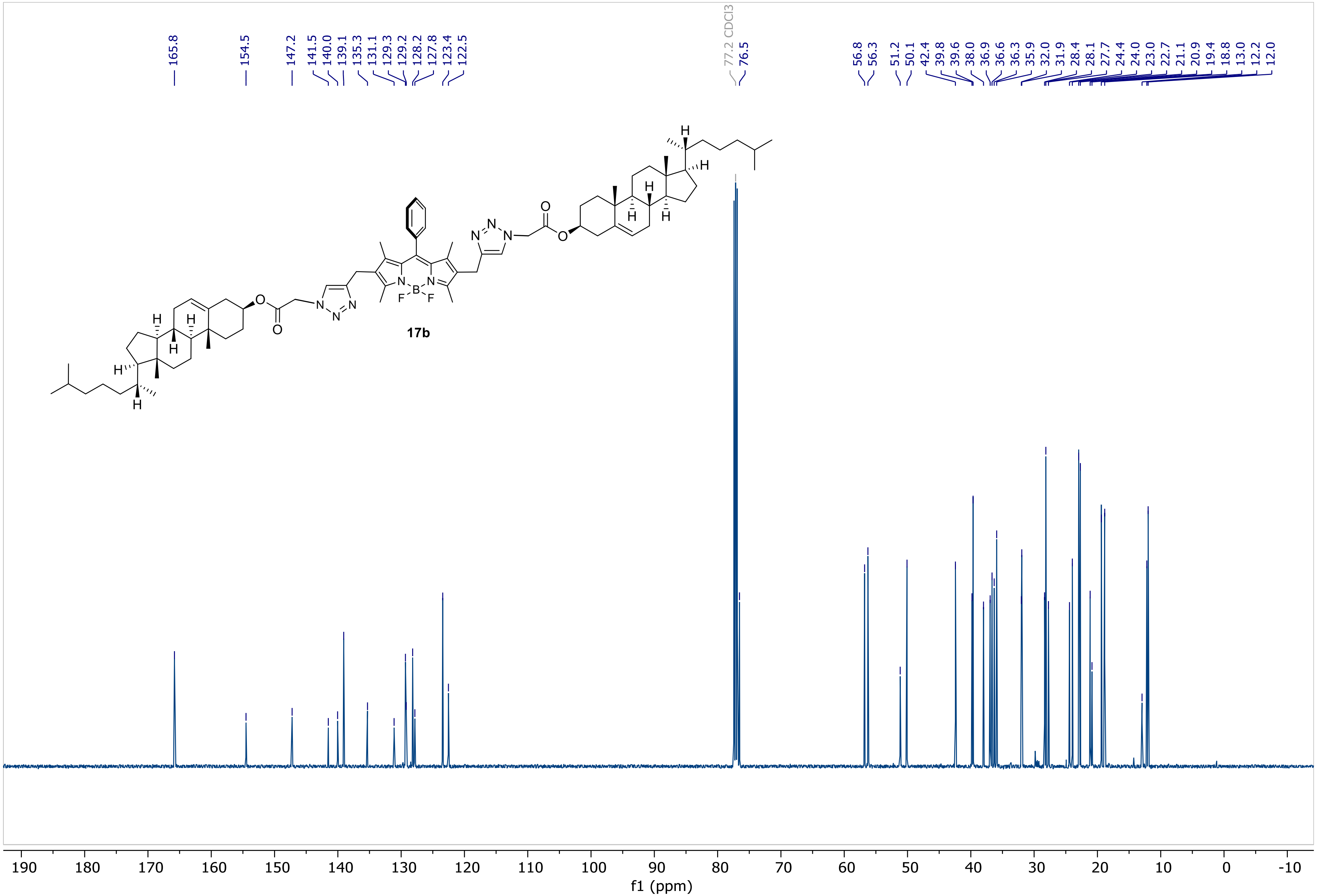

13C {1H} NMR (125 MHz, CDCl3)

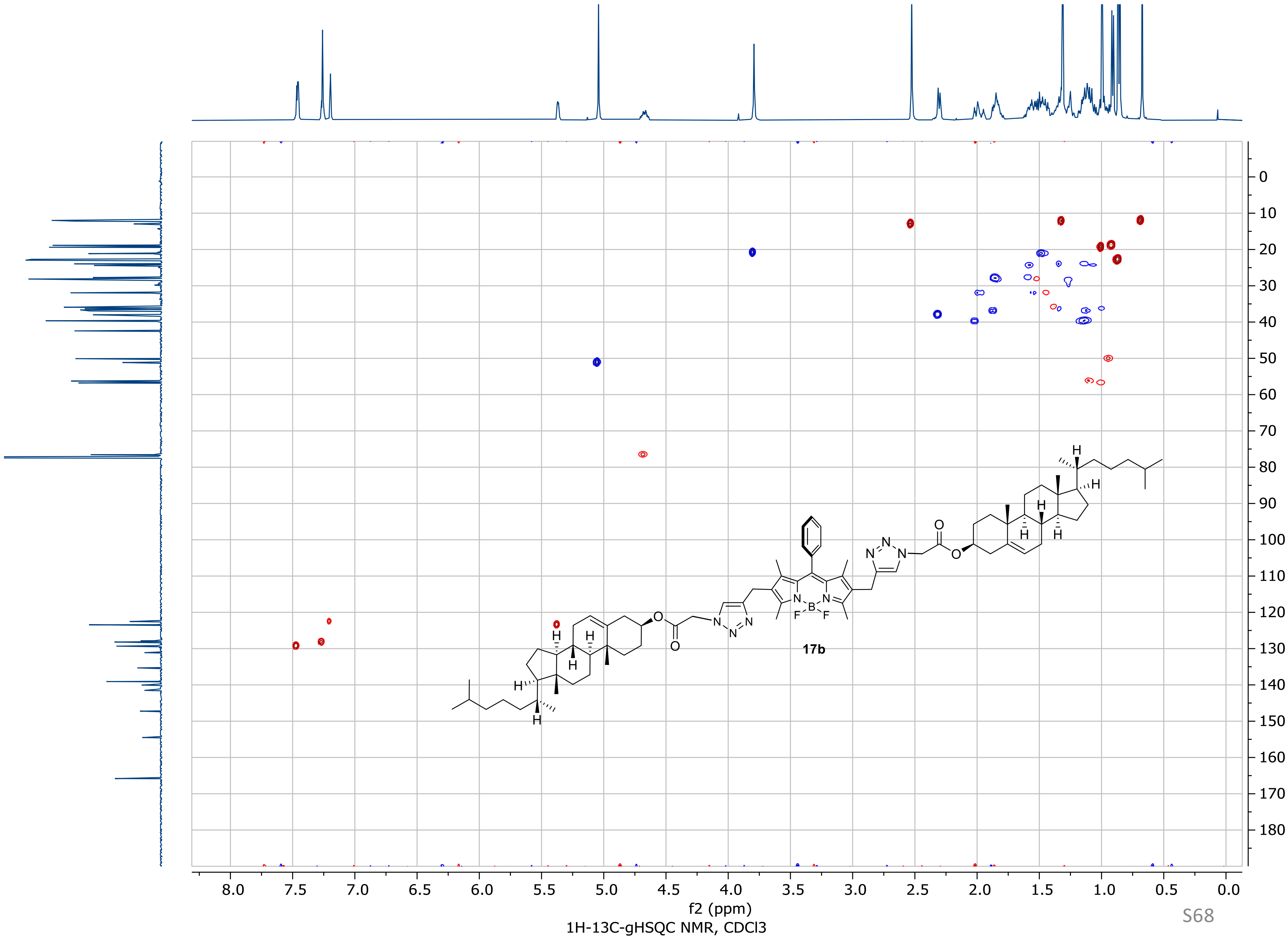
17b
f2 (ppm)
1H-13C-gHSQC NMR, CDCl3

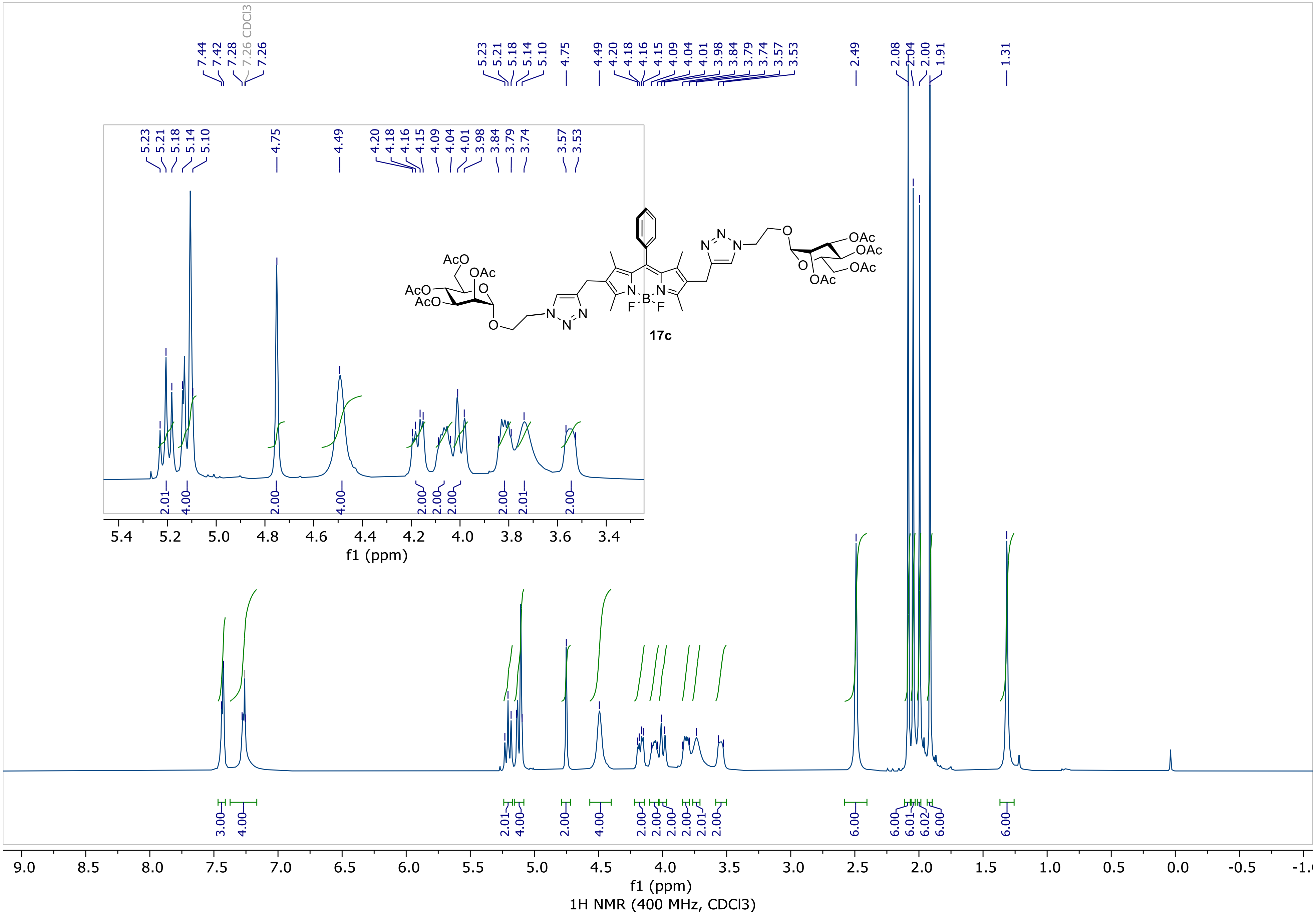
7.44 7.42 7.28 7.26 CDCl3 7.26
5.23 5.21 5.18 5.14 5.10 4.75 4.49 4.20 4.18 4.16 4.15 4.09 4.04 4.01 3.98 3.84 3.79 3.74 3.57 3.53
2.49 2.08 2.04 2.00 1.91 1.31
17c
3.00 4.00 2.01 4.00 2.00 4.00 2.00 2.00 2.00 2.00 2.01 2.00 6.00 6.00 6.01 6.02 6.00
f1 (ppm)
1H NMR (400 MHz, CDCl3)

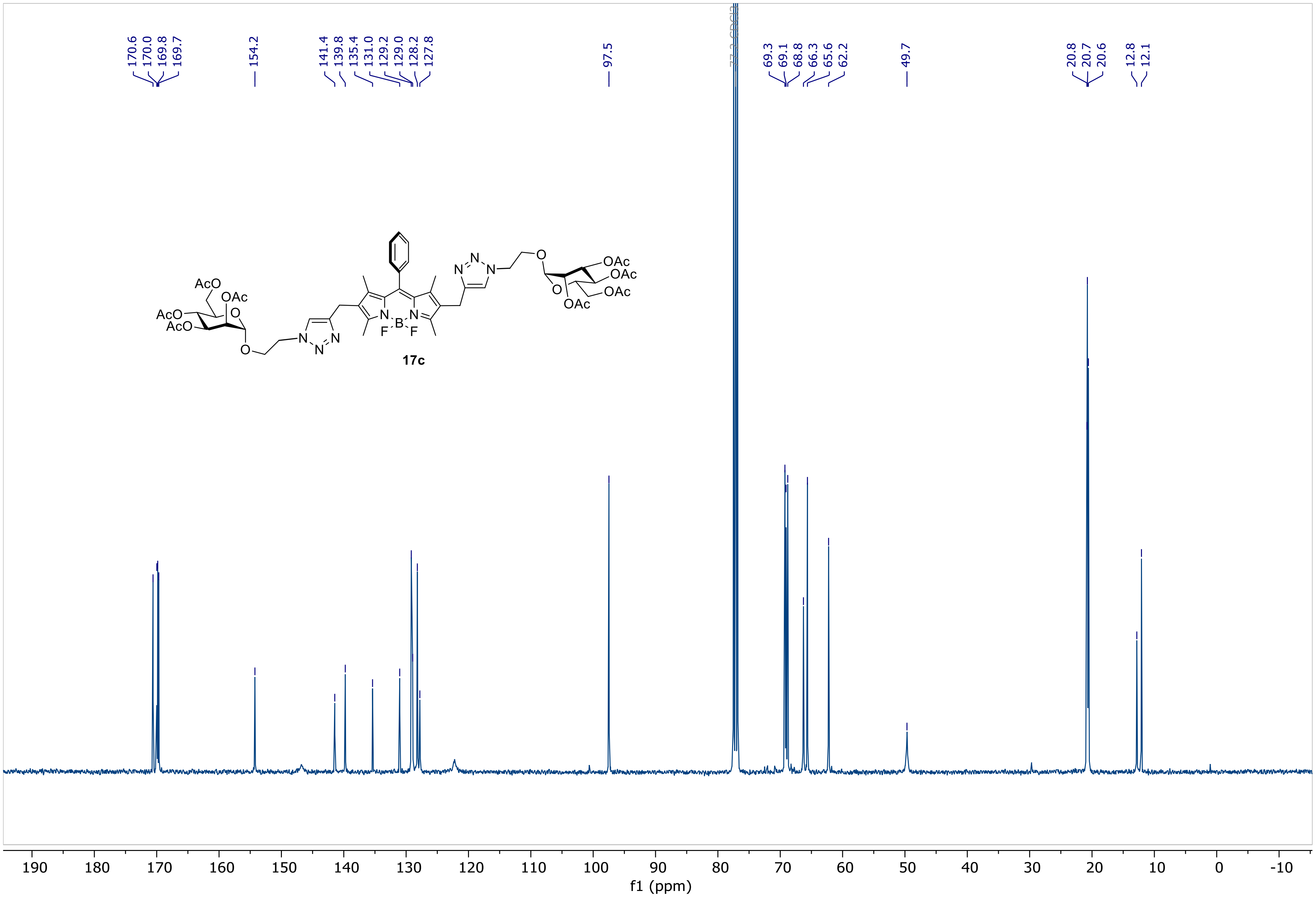

13C {1H} NMR (125 MHz, CDCl3)

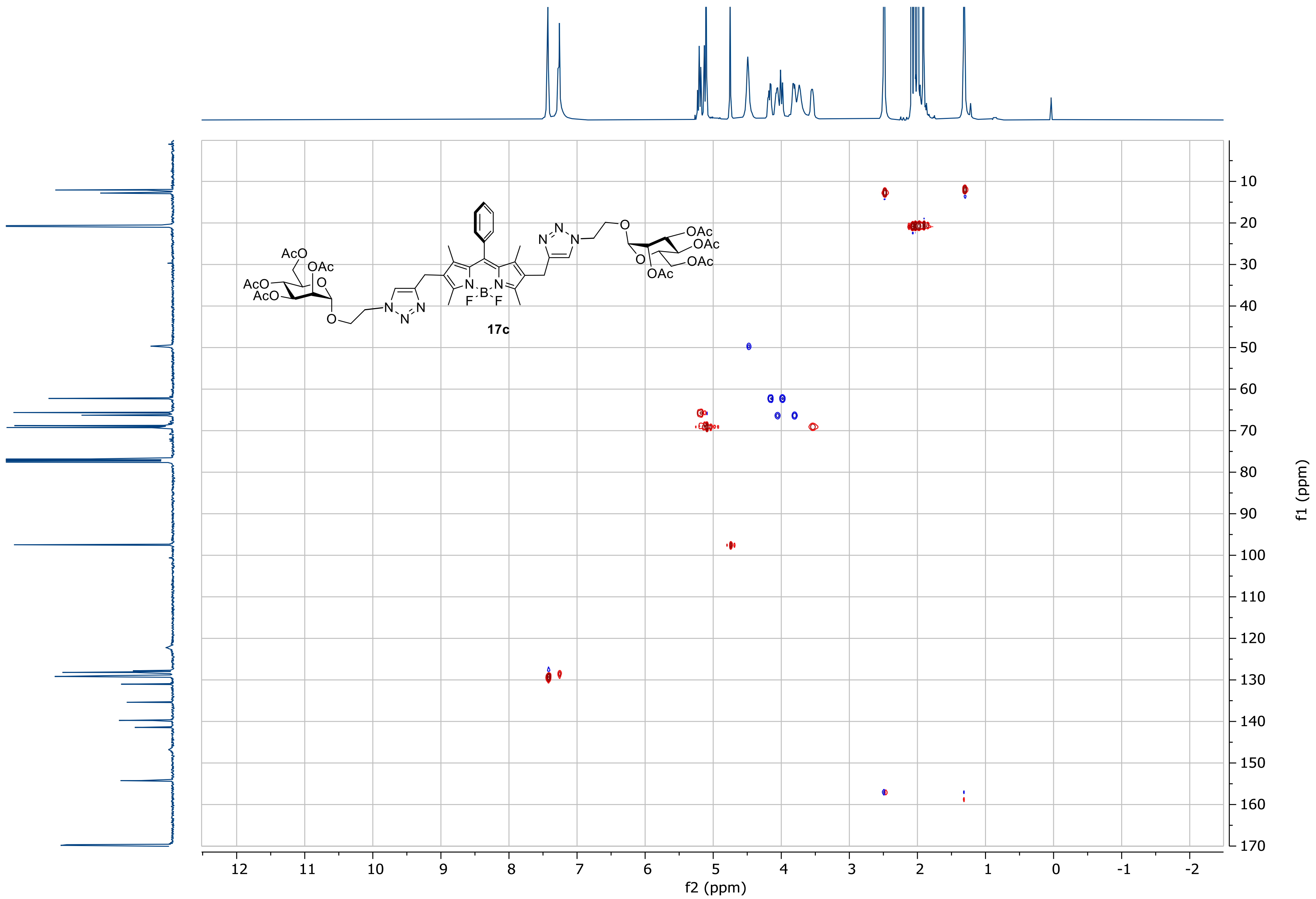

1H-13C gHSQC NMR, CDCl3

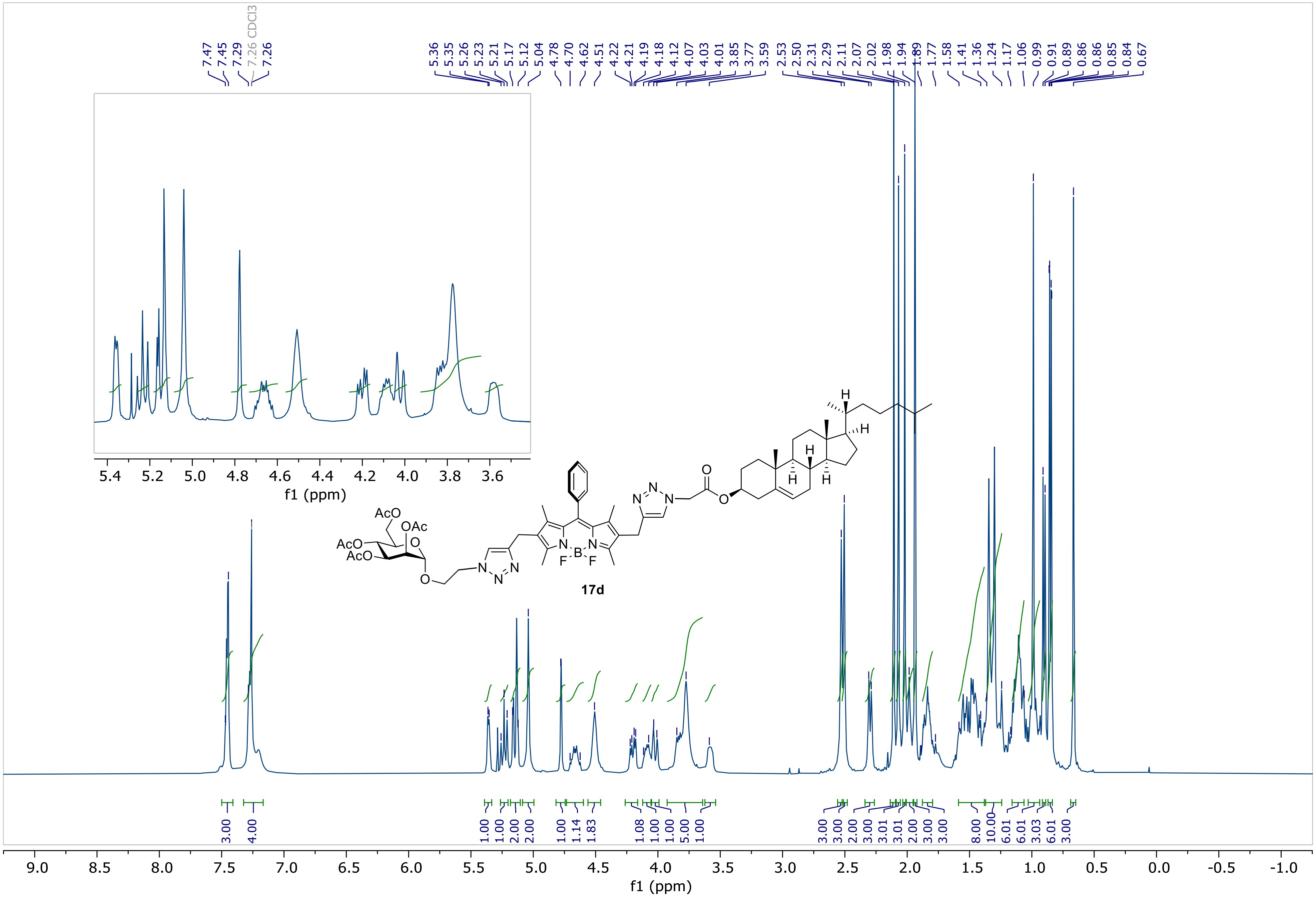

1H NMR (400 MHz, CDCl3)

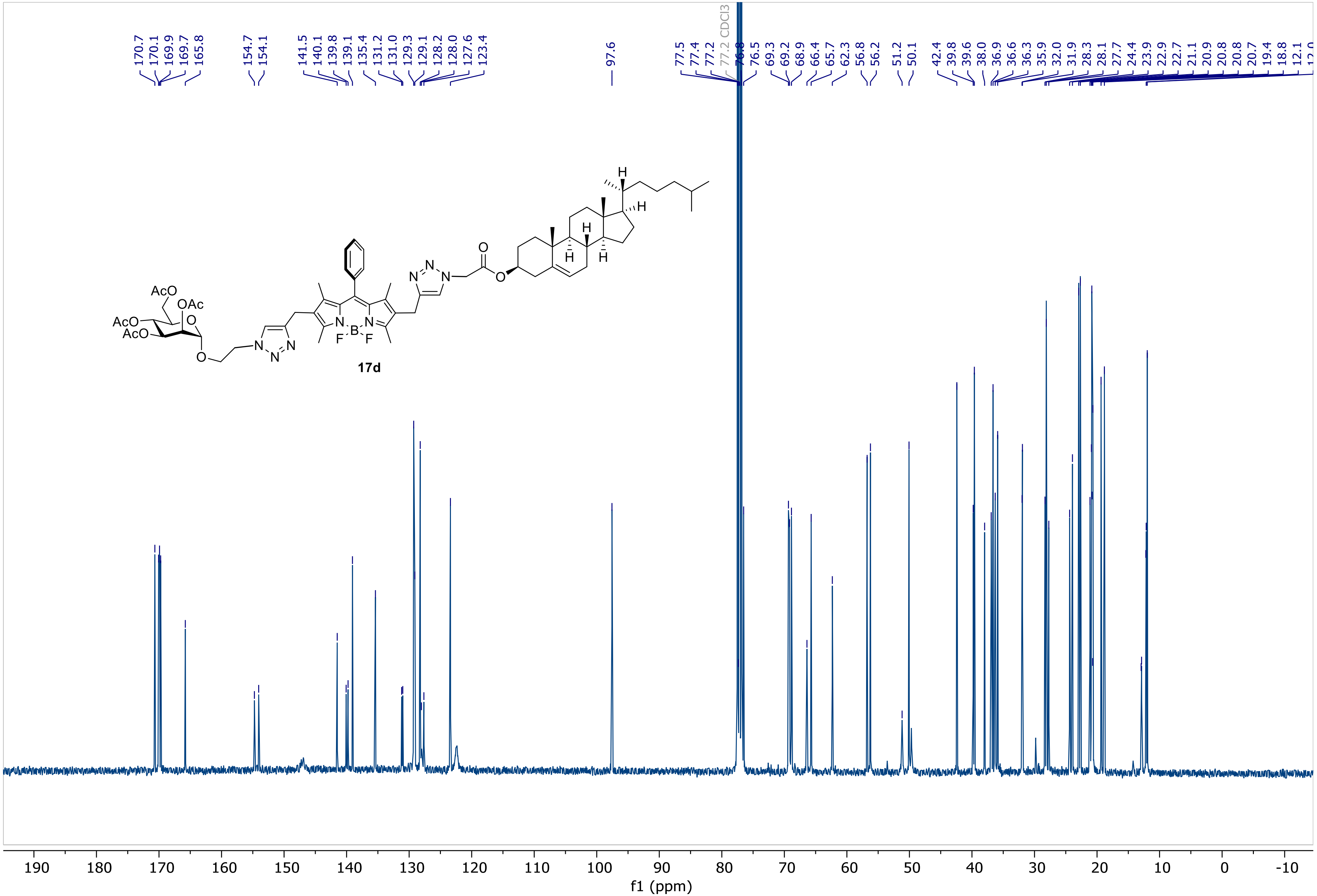

13C {1H} NMR (125 MHz, CDCl3)

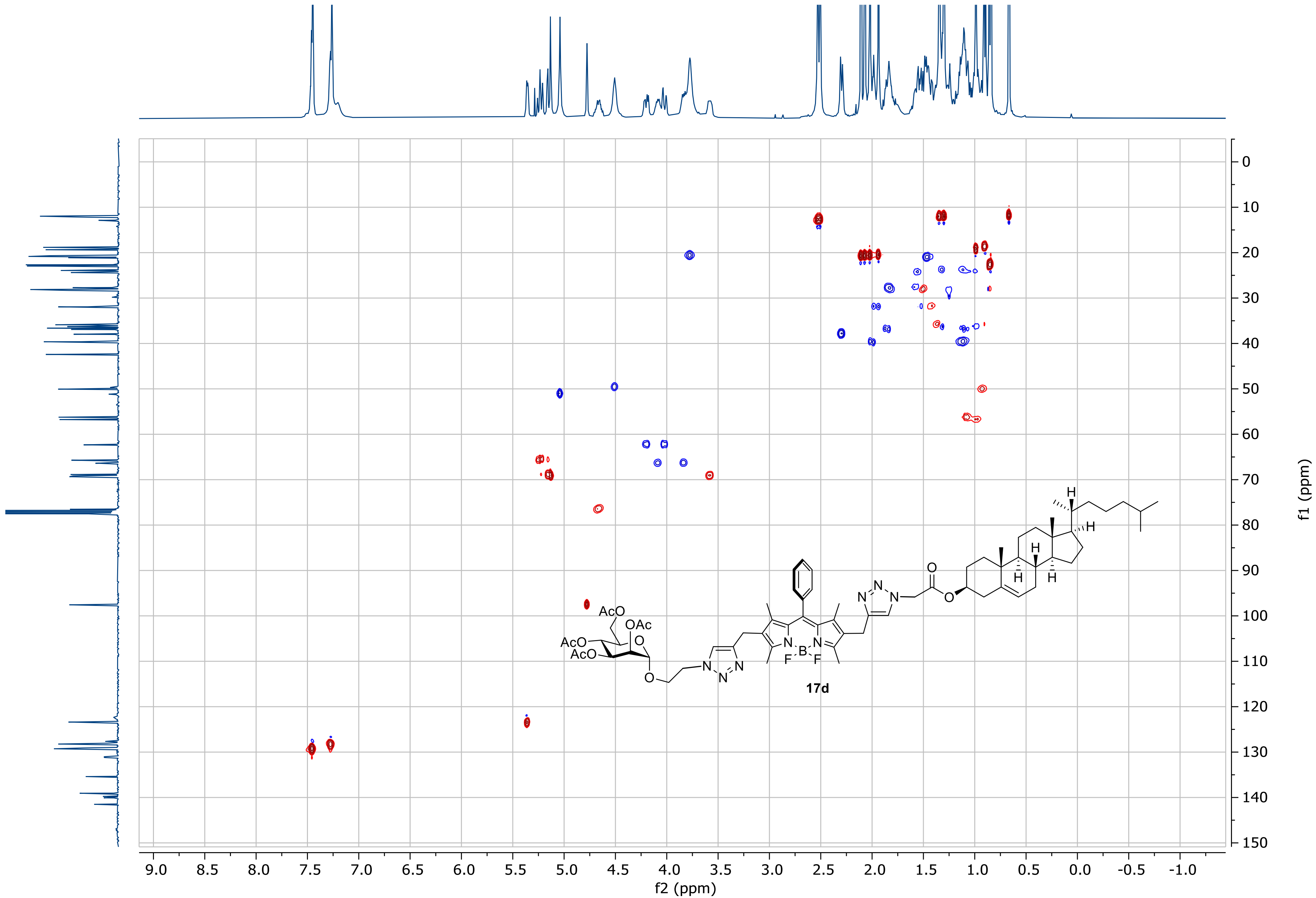

1H-13C gHSQC NMR, CDCl3

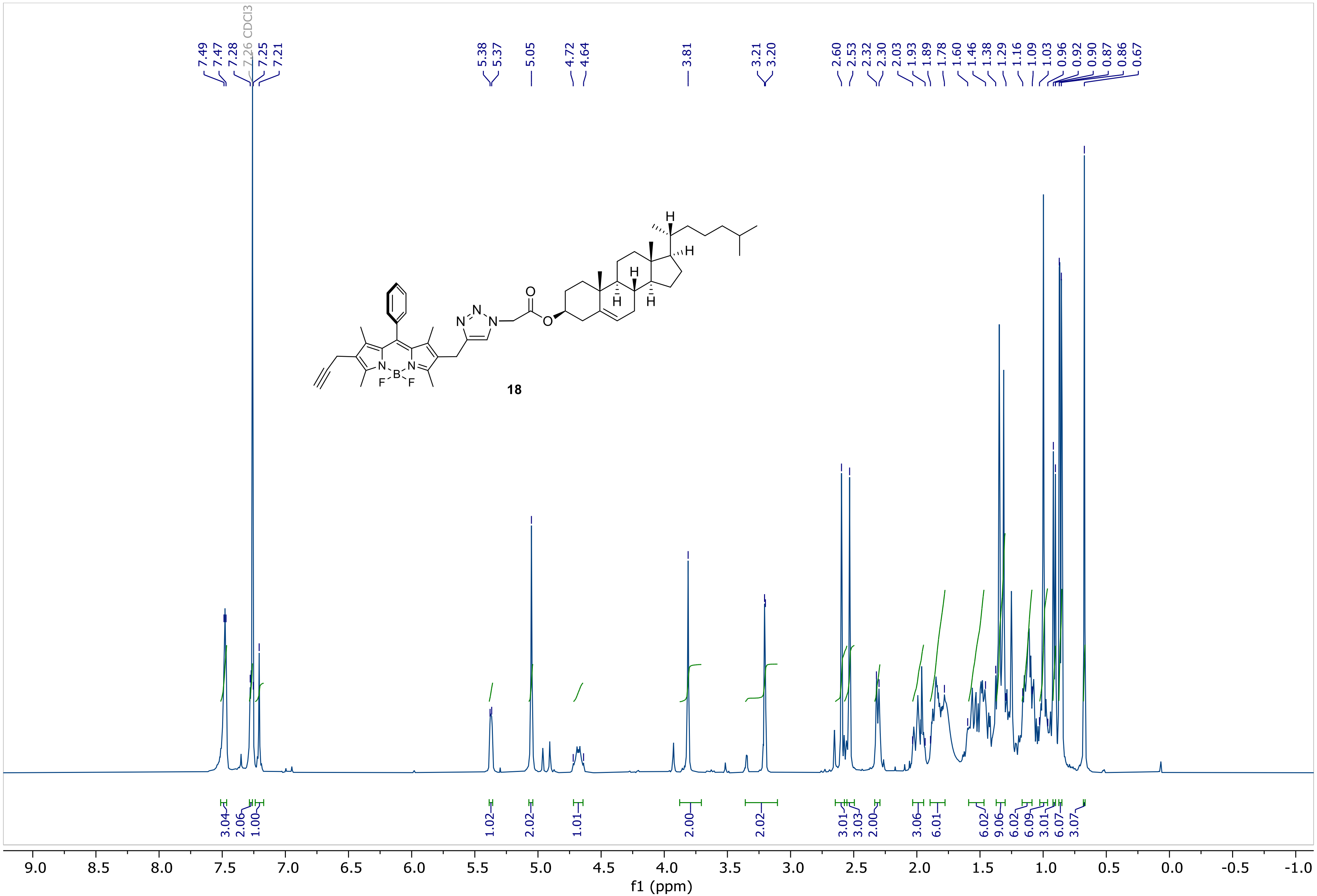

1H NMR (400 MHz, CDCl3)

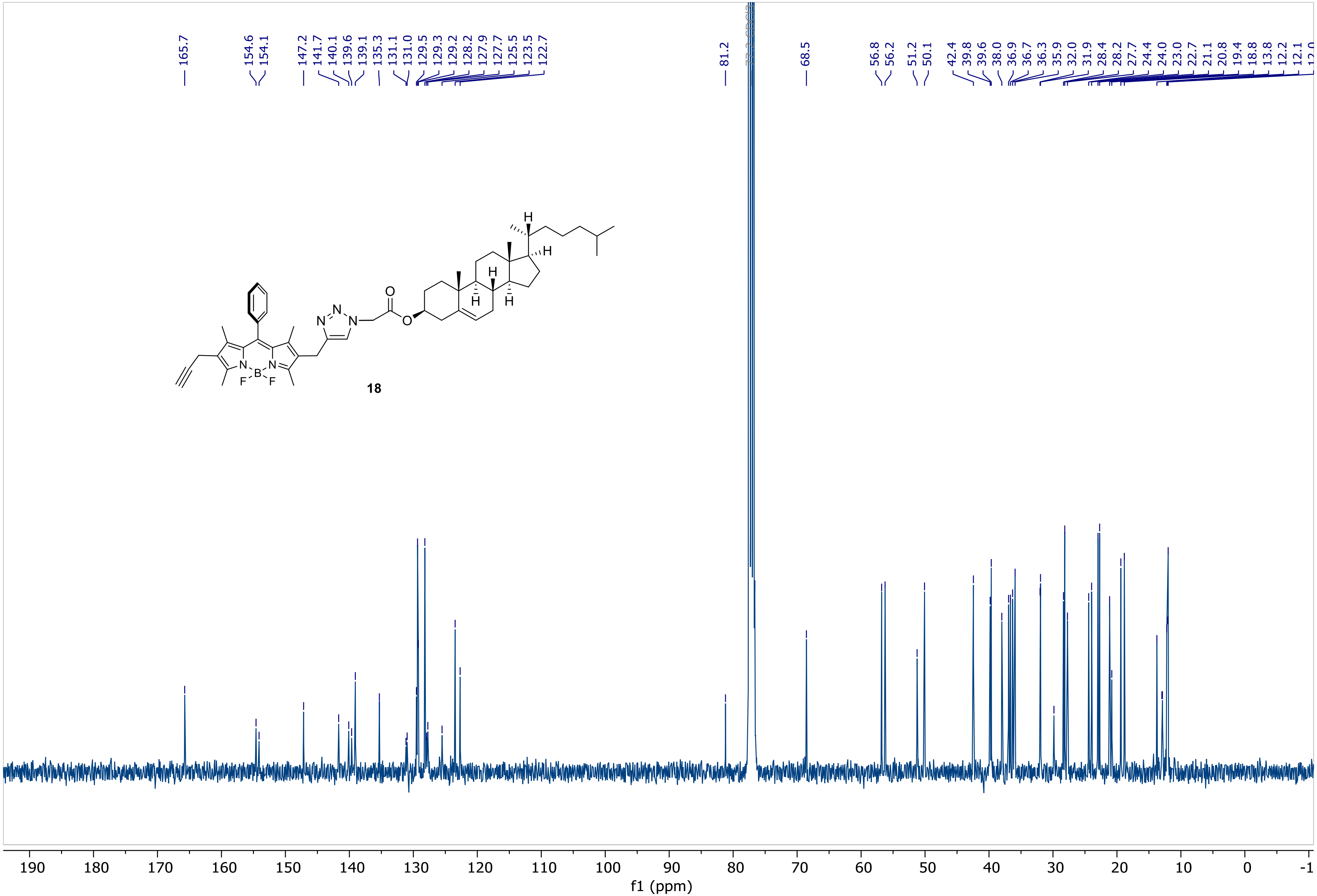

13C {1H} NMR (125 MHz, CDCl3)

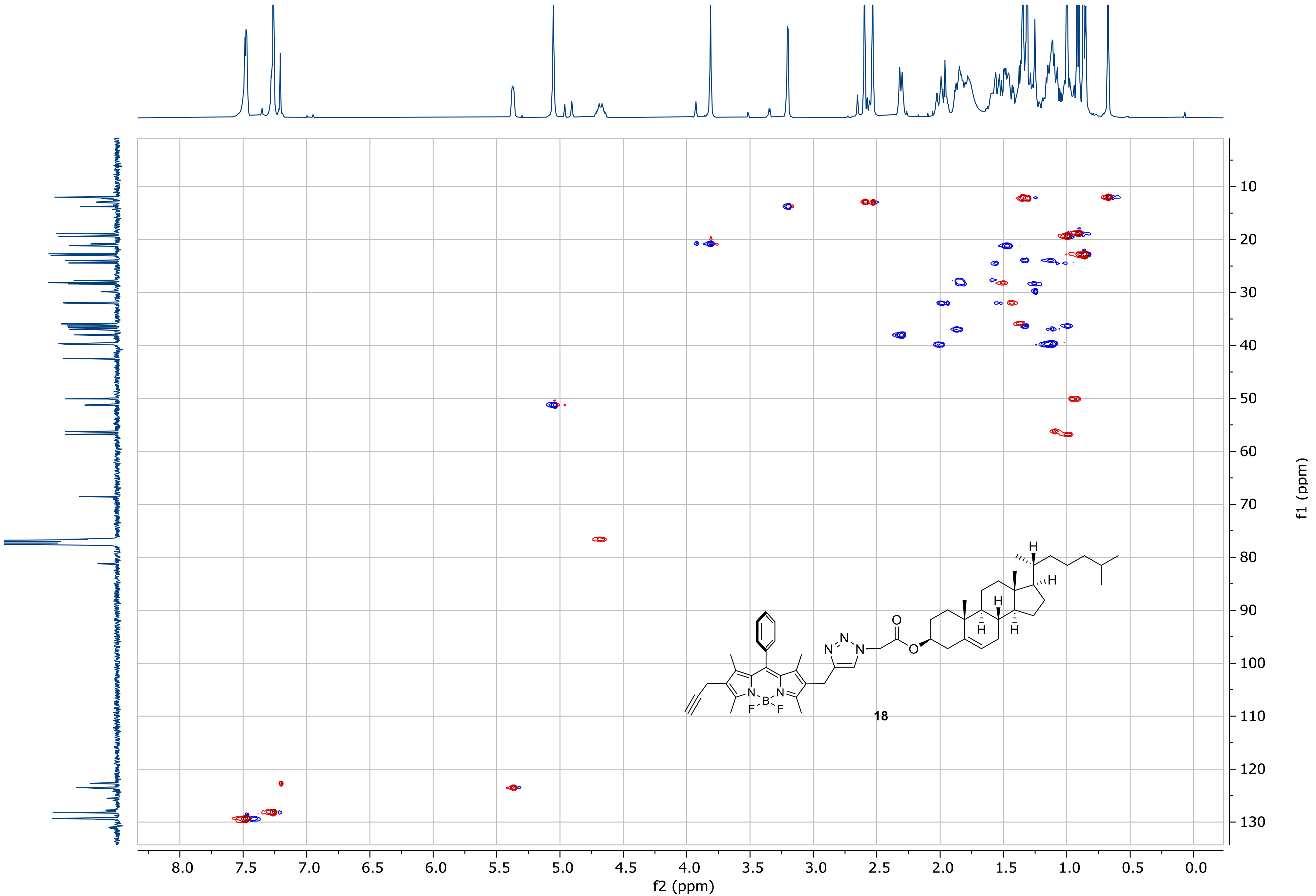

1H- 13C gHSQC, CDCl3

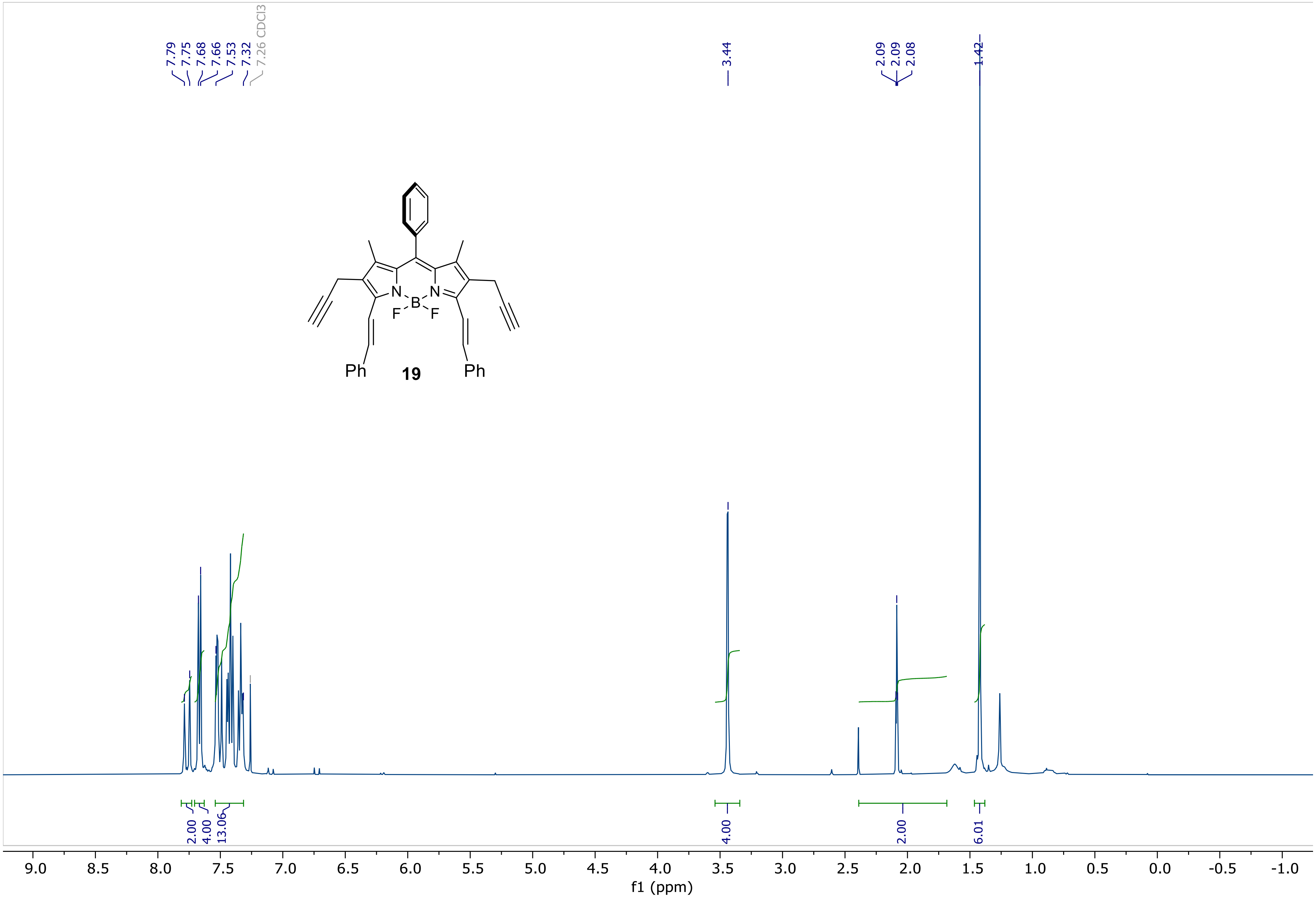

1H NMR (400 MHz, CDCl3)

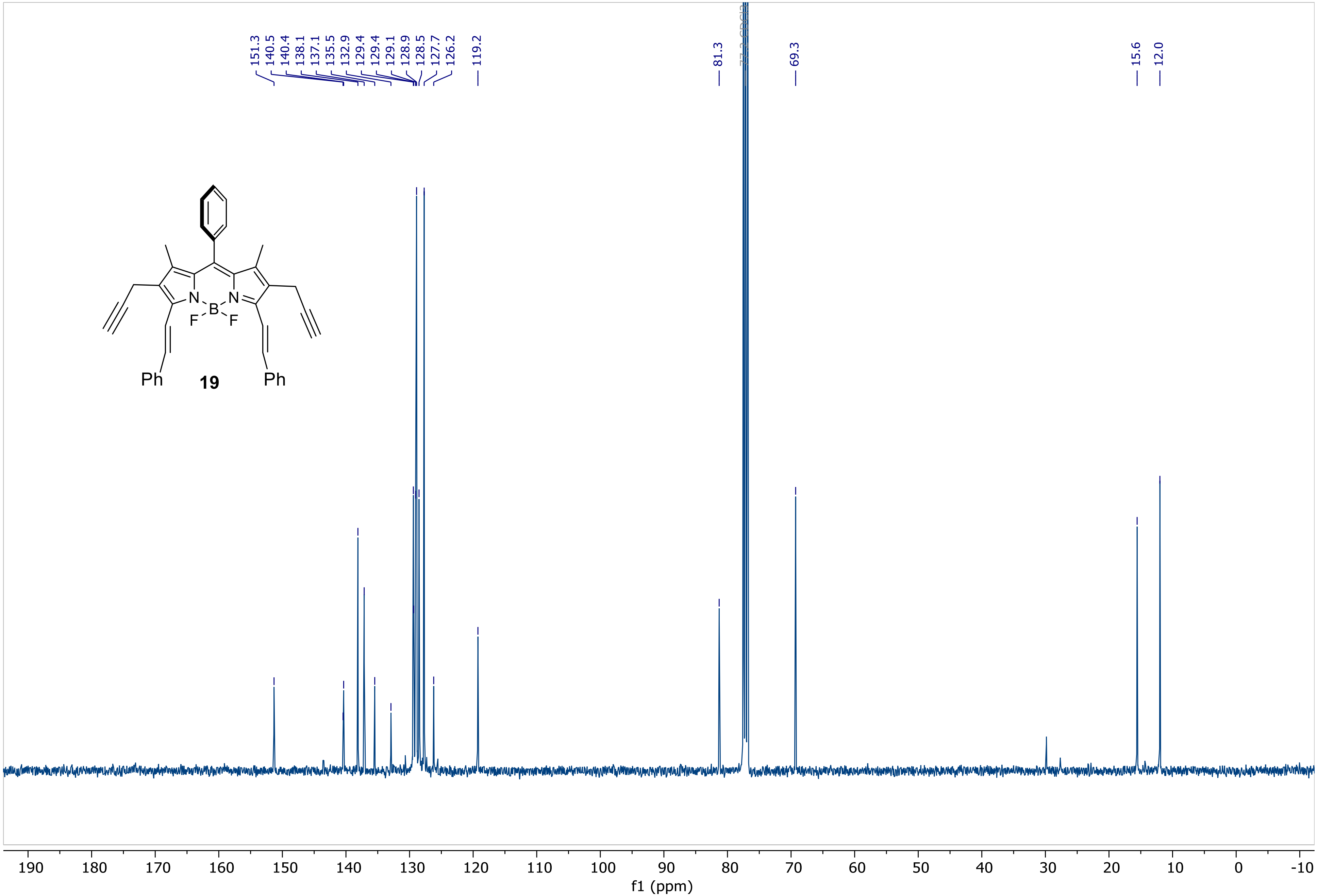

13C {1H} NMR (125 MHz, CDCl3)

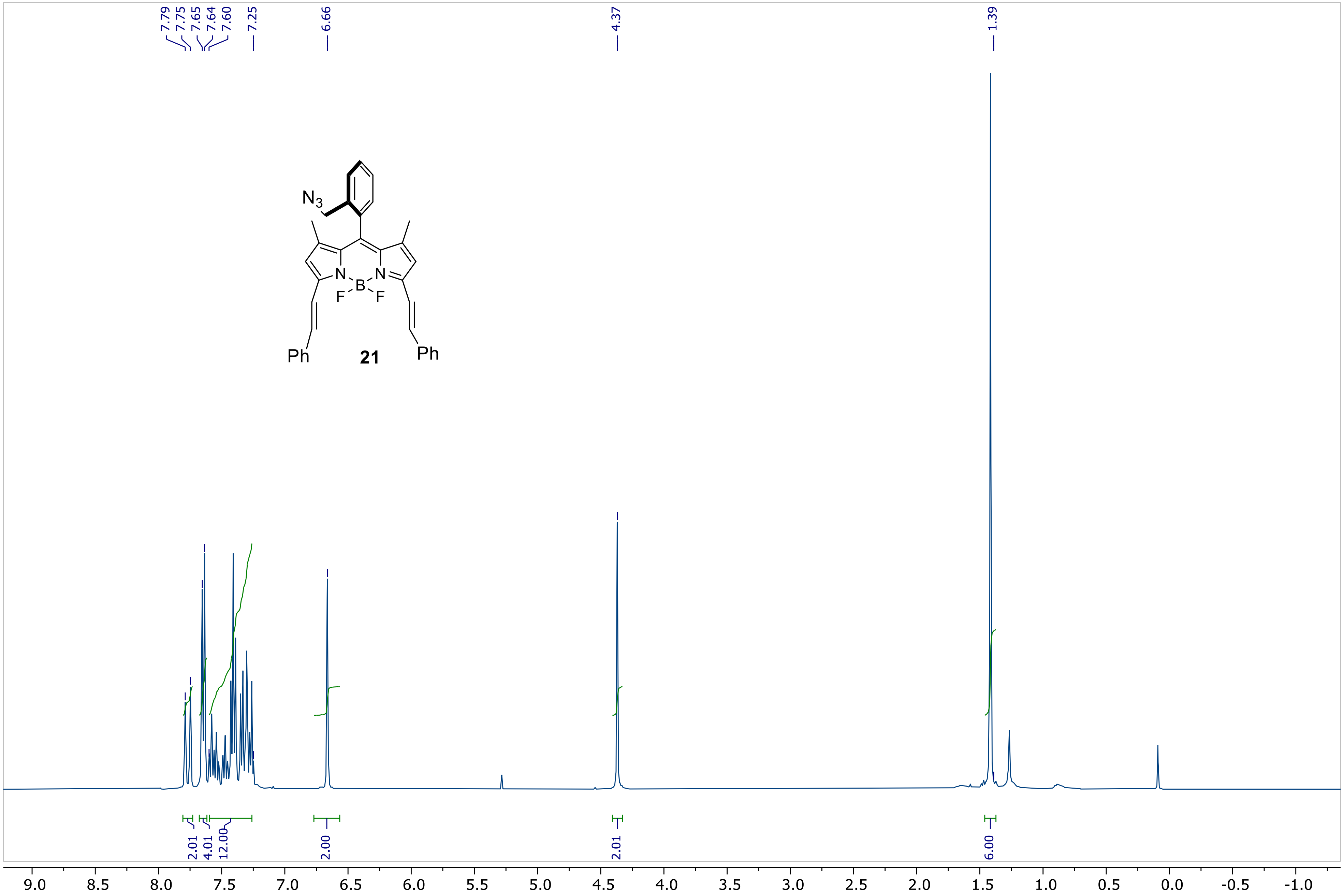

1H- NMR (400 MHz, CDCl3)

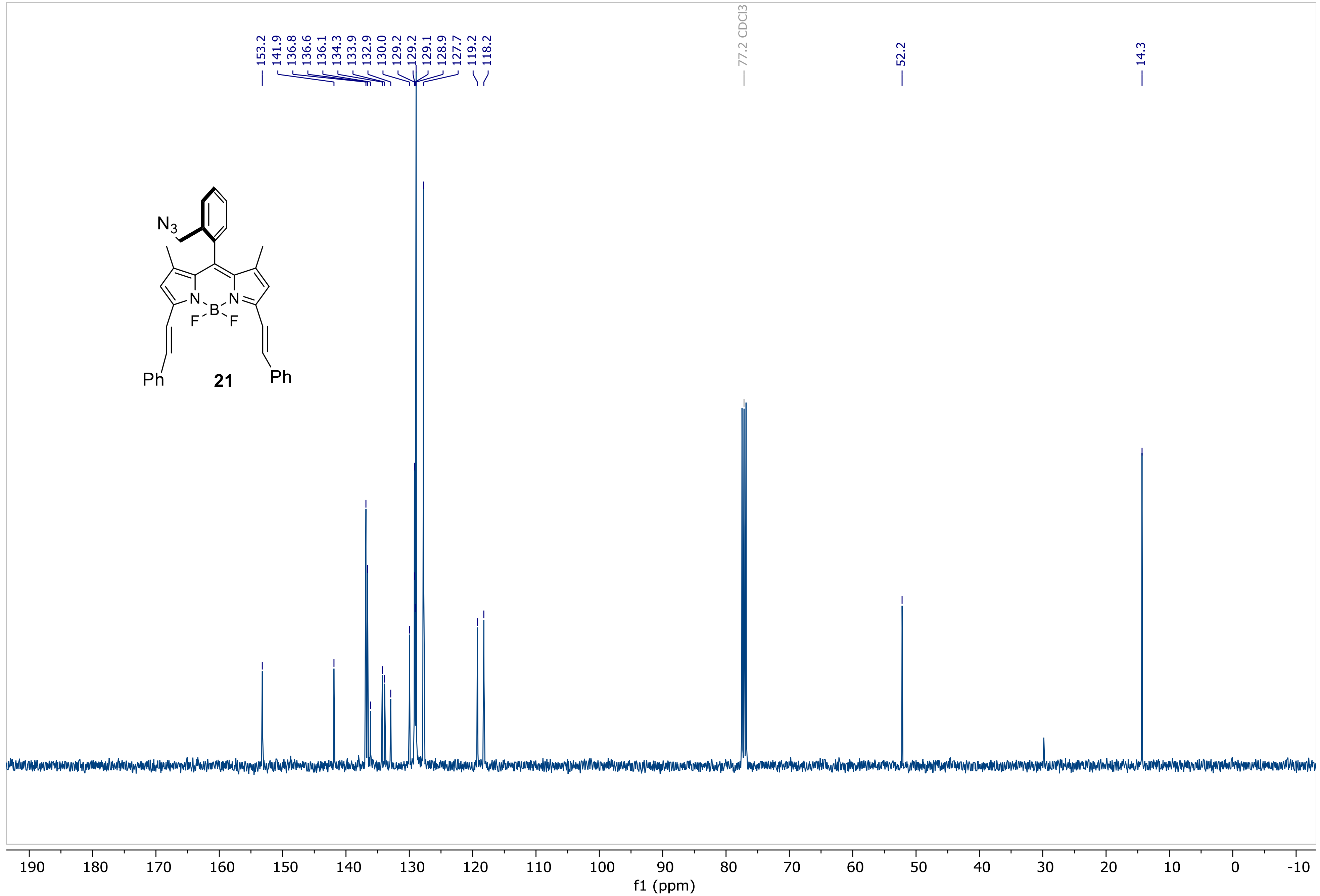

13C {1H} NMR (125 MHz, CDCl3)

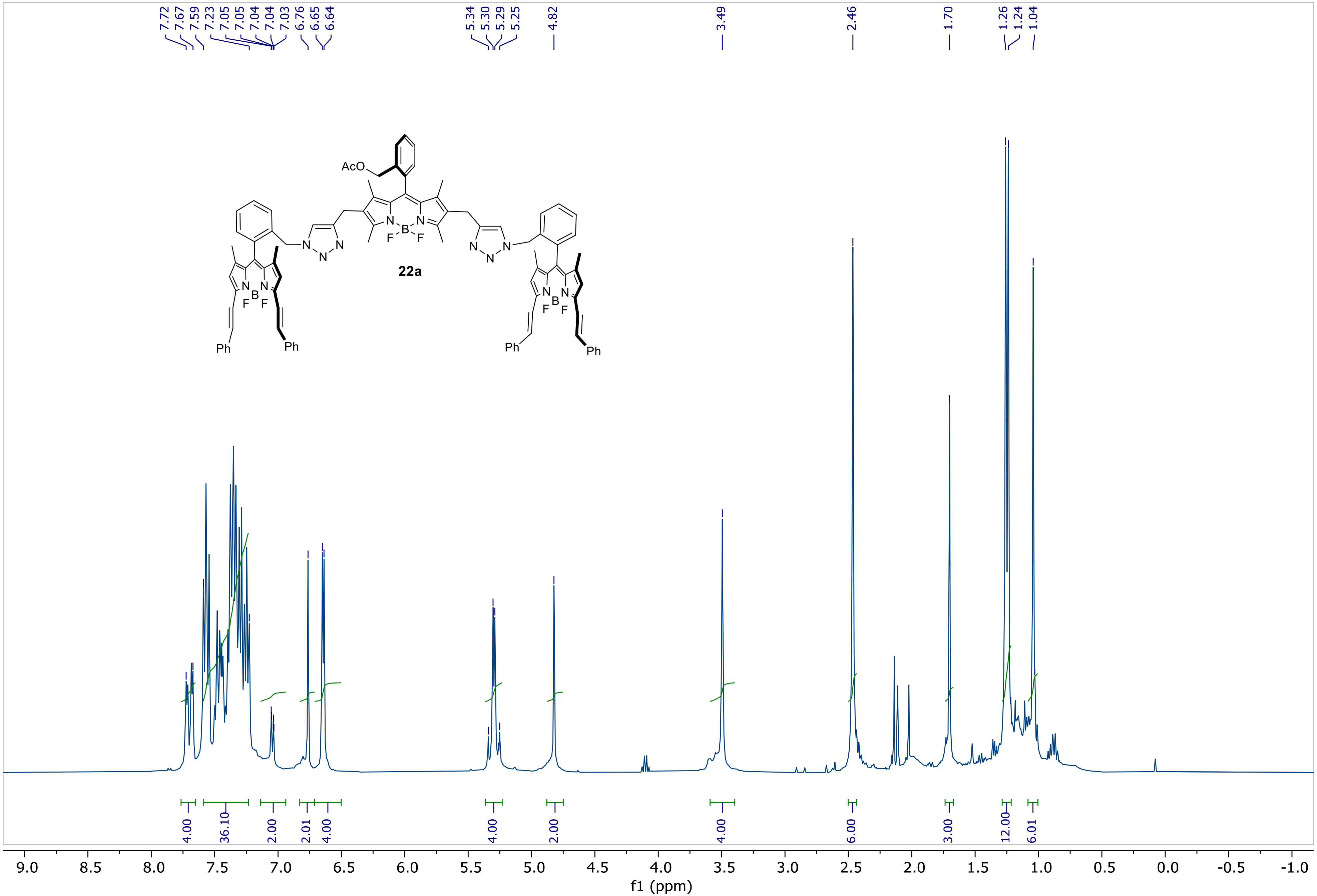

1H NMR (400 MHz, CDCl3)

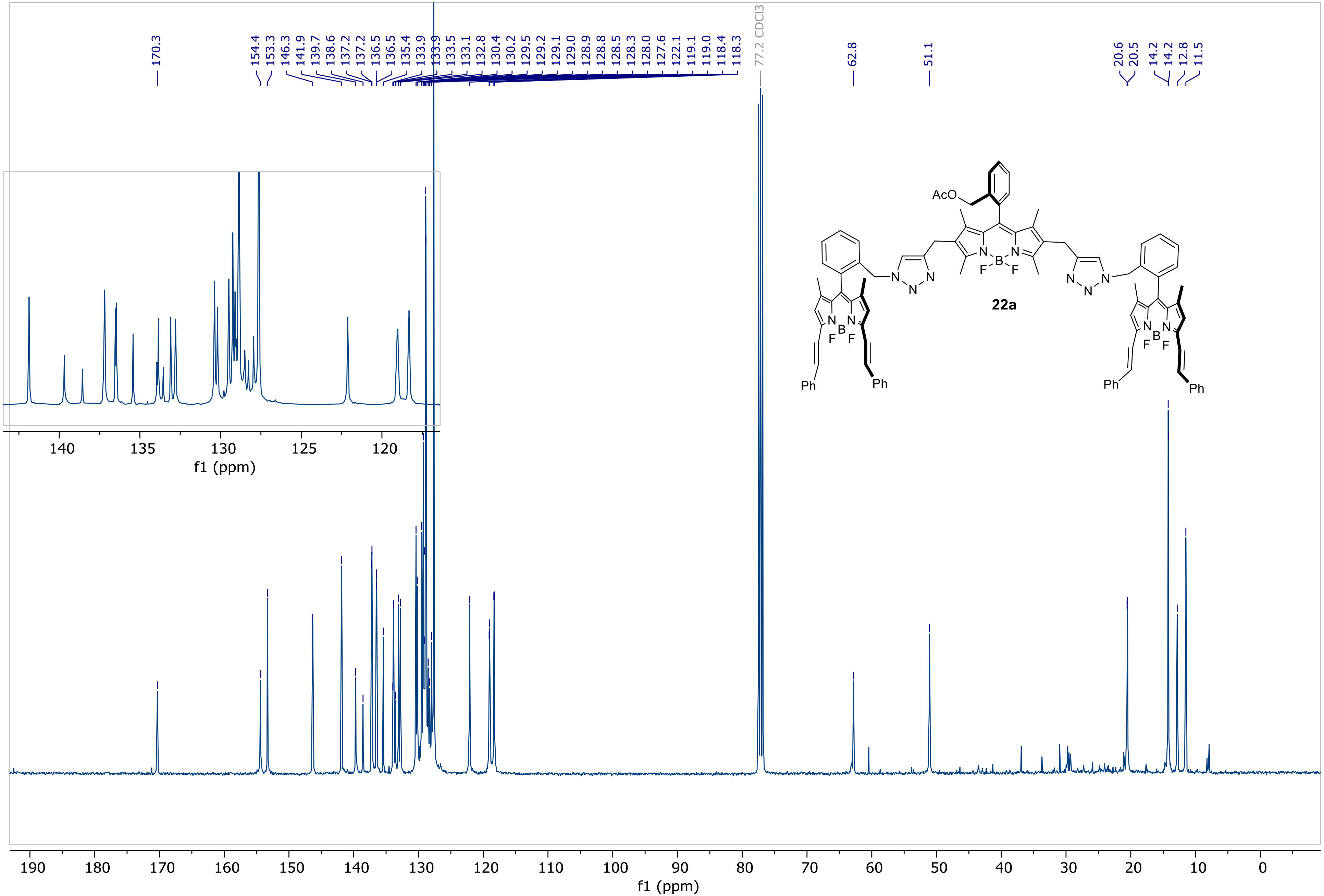

13C {1H} NMR (125 MHz, CDCl3)

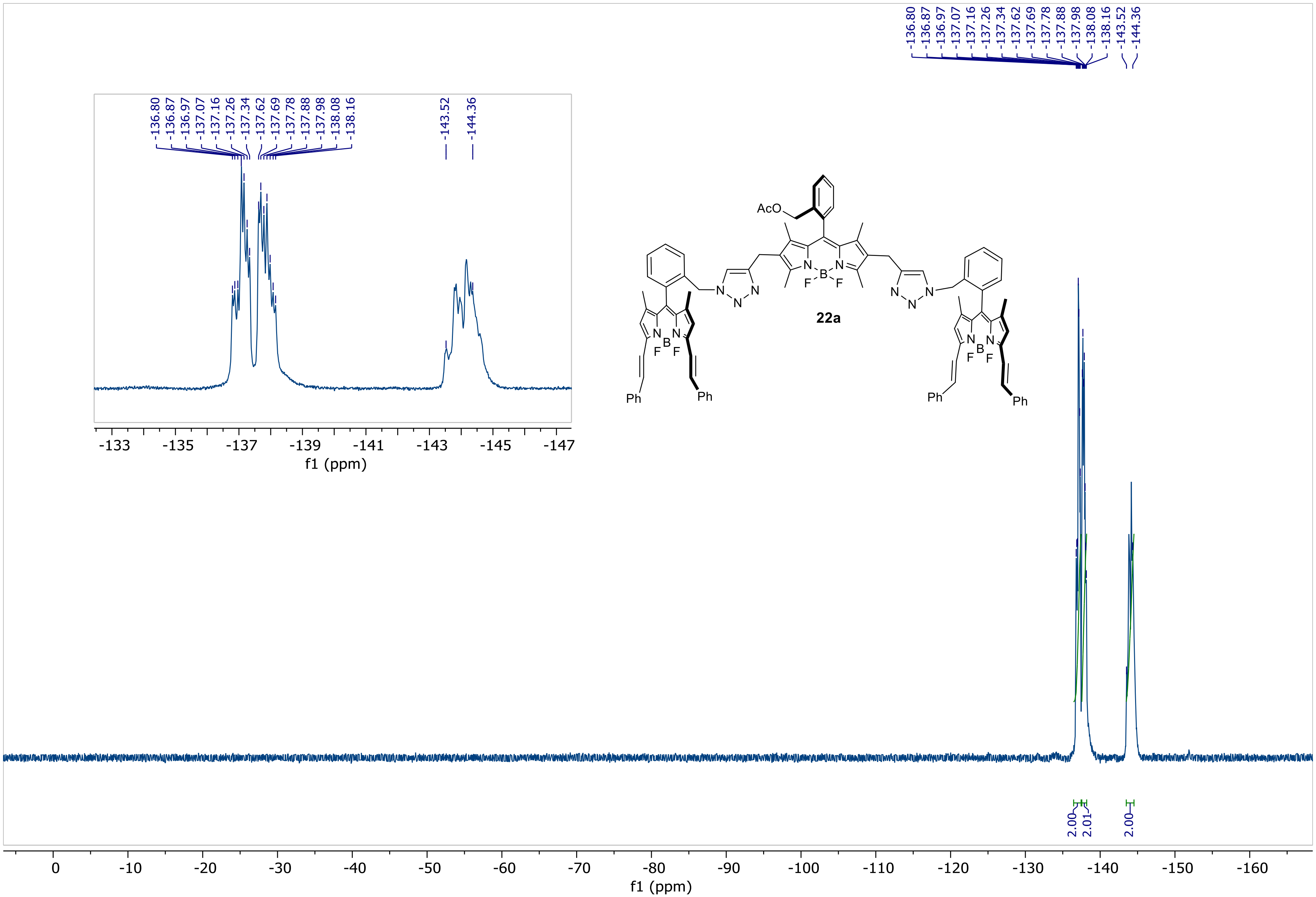

19F NMR (376 MHz, CDCl3)

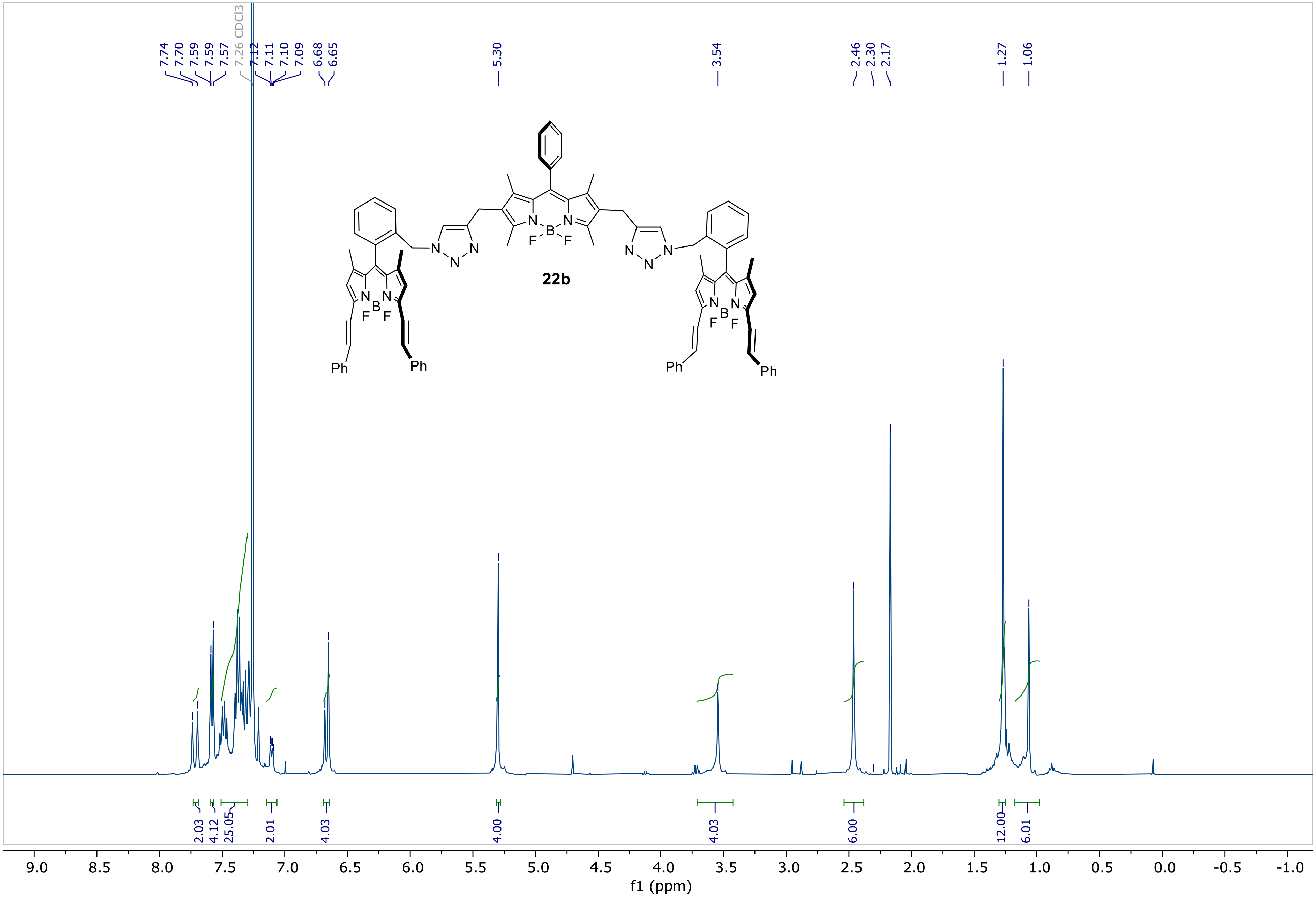

1H NMR (400 MHz, CDCl3)

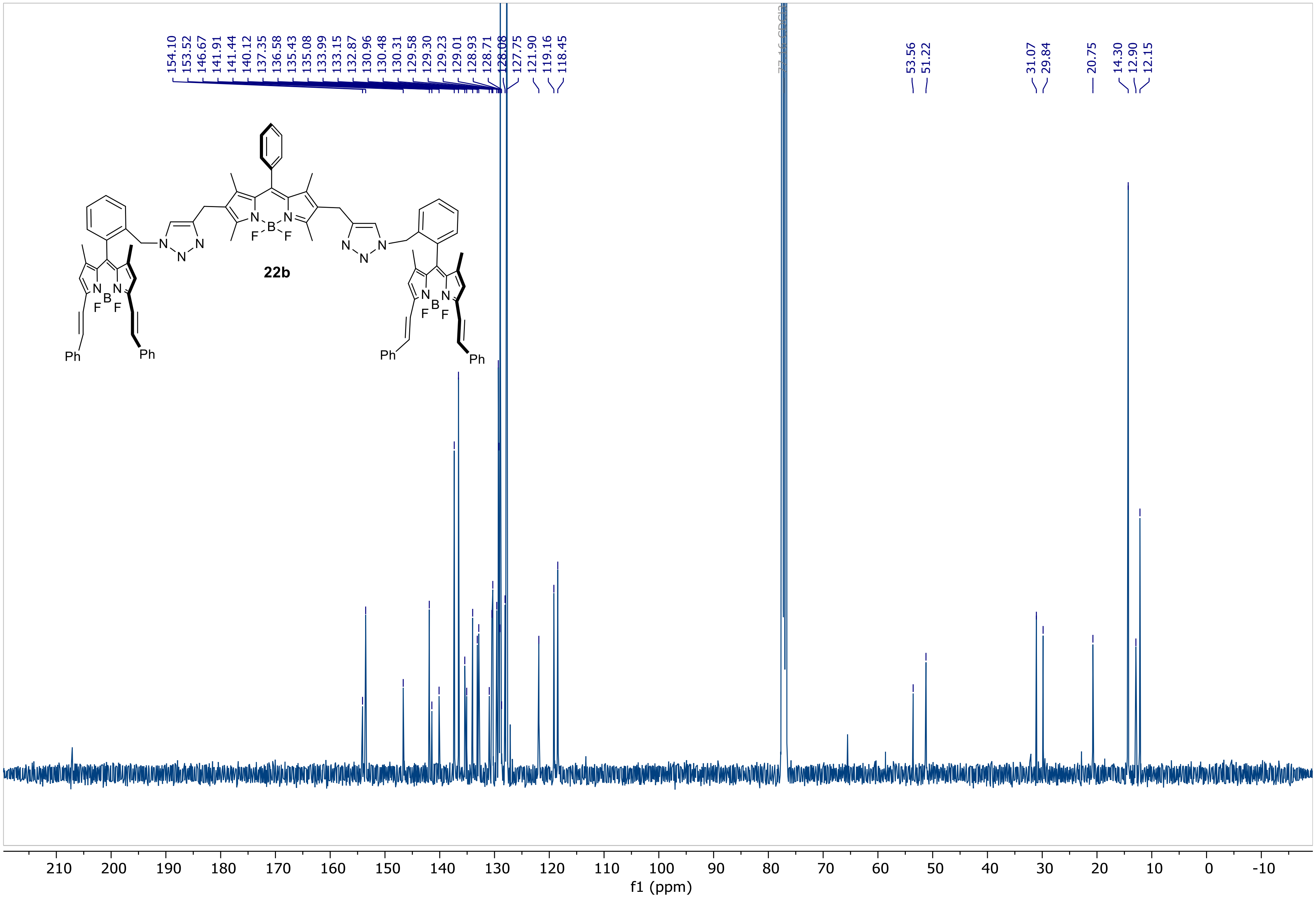

13C {1H} NMR (CDCl3, 125 MHz)

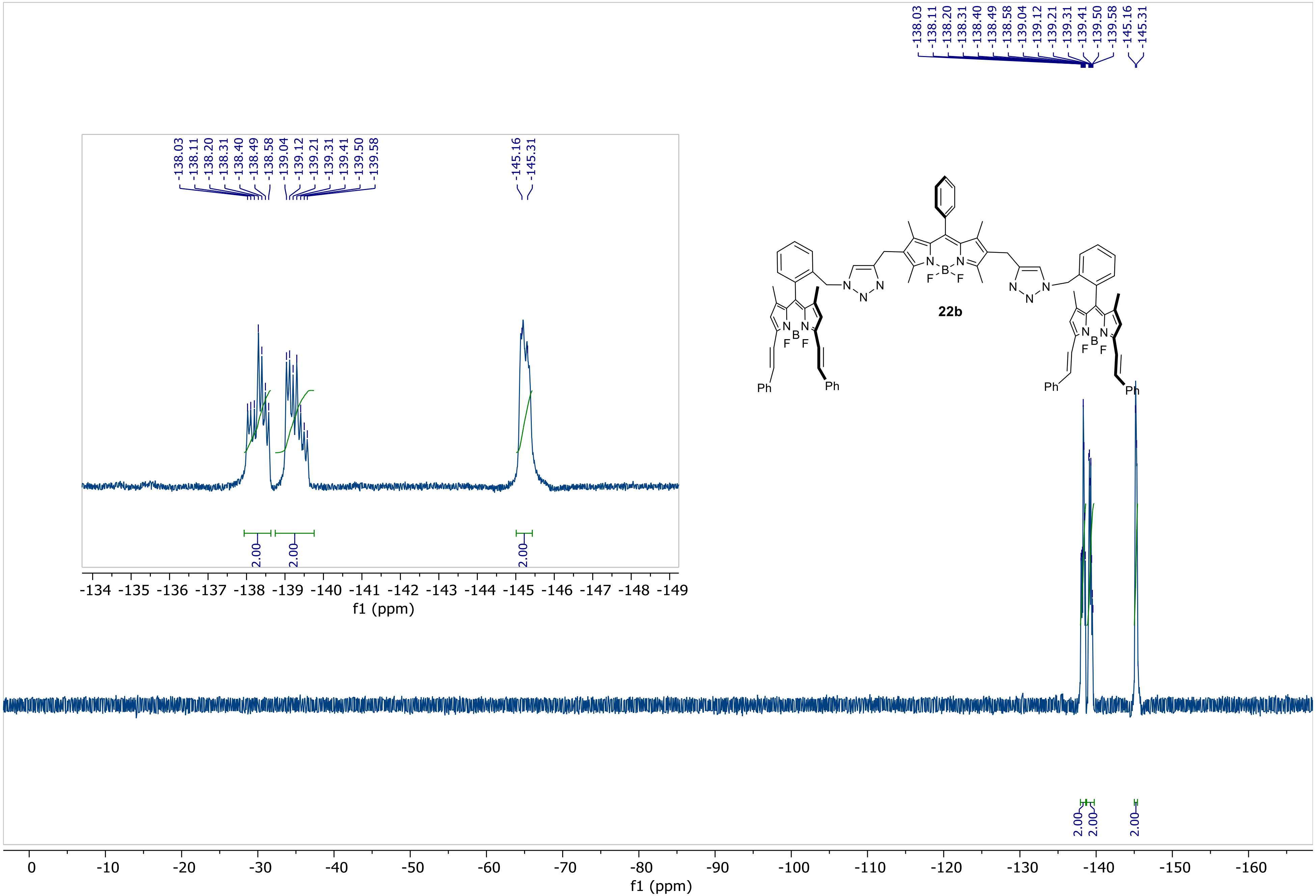

19F NMR (376 MHz, CDCl3)

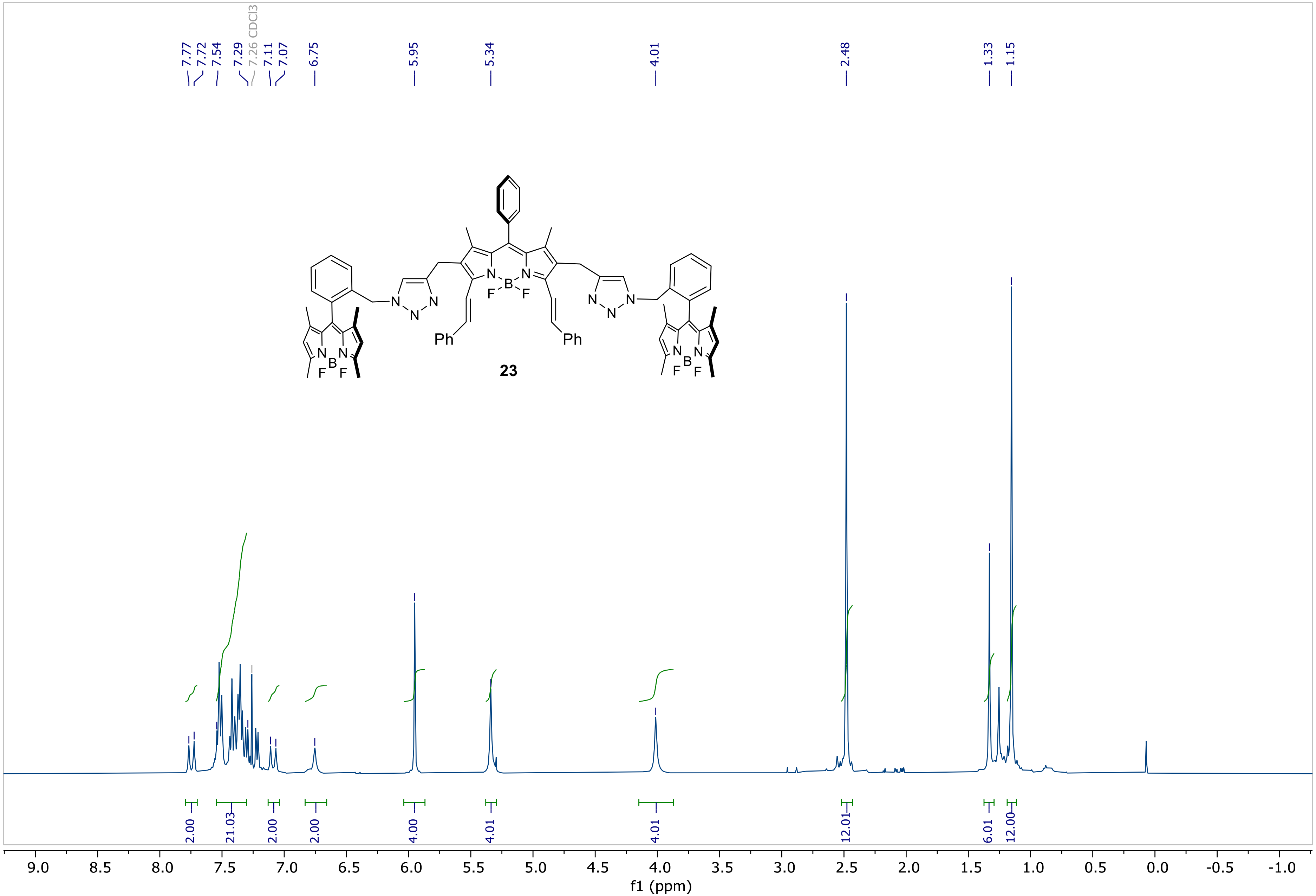

1H NMR (400 MHz, CDCl3)

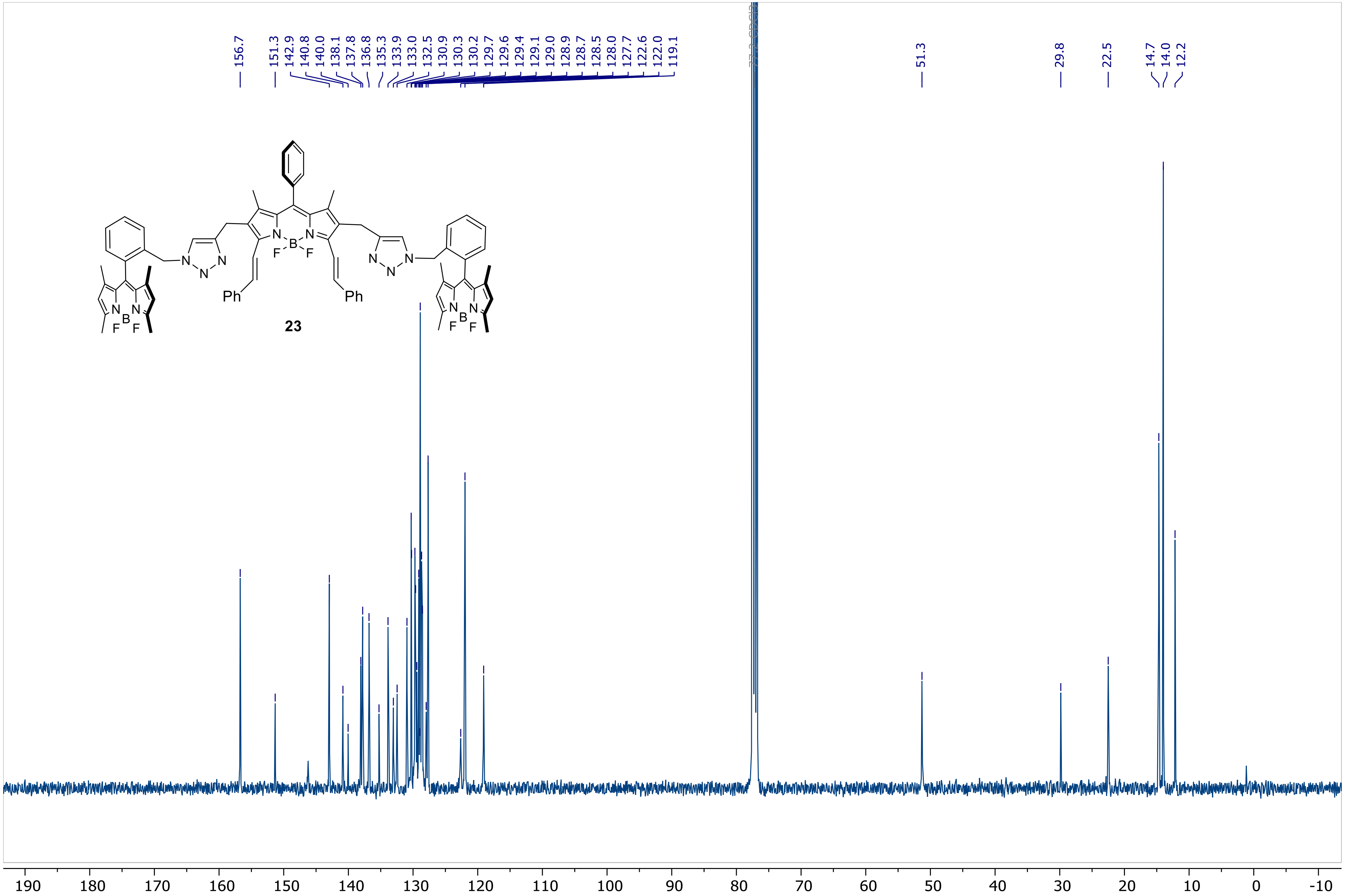

f1 (ppm)

1H NMR (400 MHz, CDCl3)

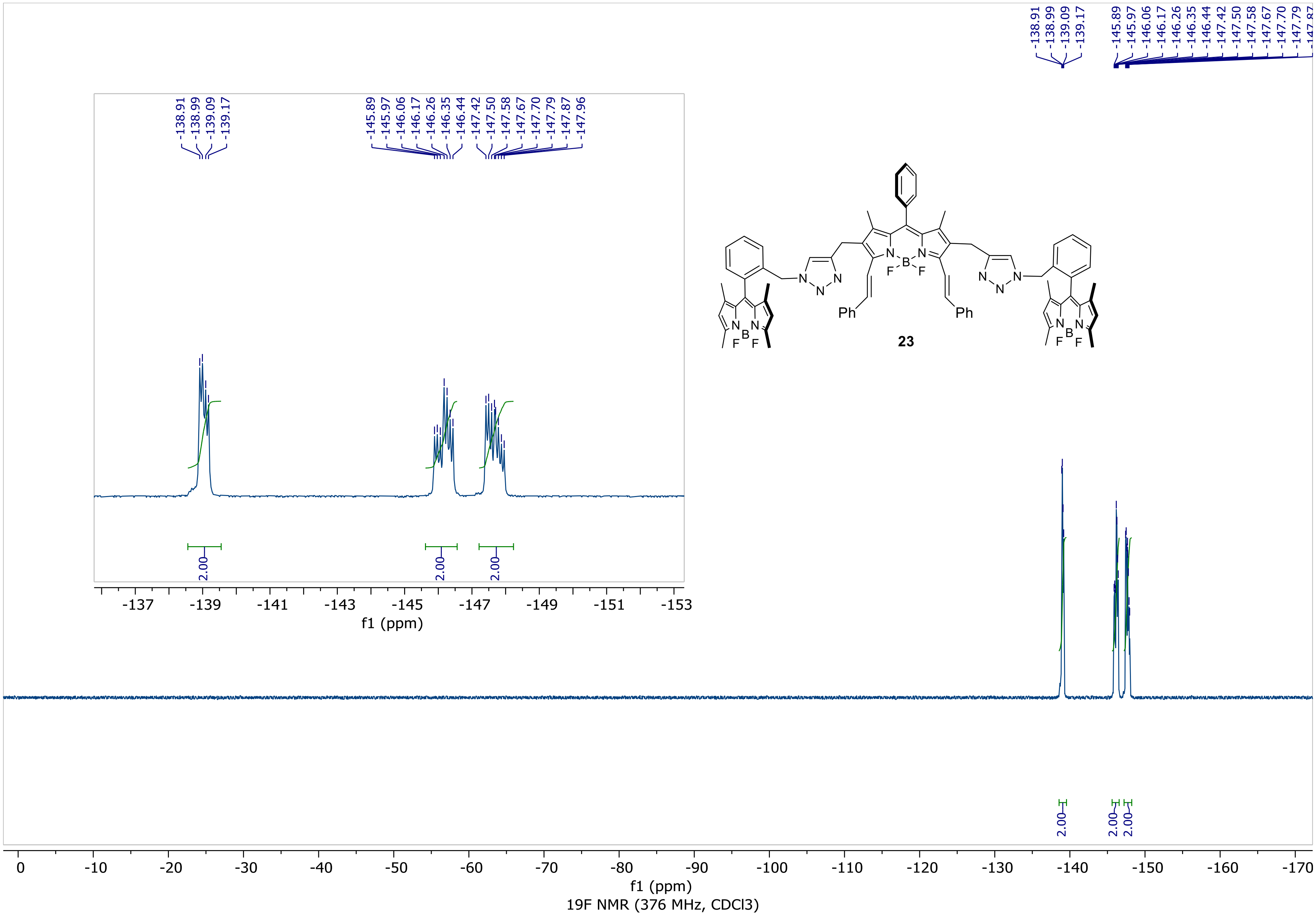
-138.91
-138.99
-139.09
-139.17
-145.89
-145.97
-146.06
-146.17
-146.26
-146.35
-146.44
-147.42
-147.50
-147.58
-147.67
-147.70
-147.79
-147.87
-147.96
2.00
2.00
2.00
f1 (ppm)
23
Ph
Ph
19F NMR (376 MHz, CDCl3)
